# Phenotype switching of the mutation rate facilitates adaptive evolution

**DOI:** 10.1093/genetics/iyad111

**Published:** 2023-06-09

**Authors:** Gabriela Lobinska, Yitzhak Pilpel, Yoav Ram

**Affiliations:** Department of Molecular Genetics, Weizmann Institute of Science, Rehovot 76100, Israel; Department of Molecular Genetics, Weizmann Institute of Science, Rehovot 76100, Israel; School of Zoology, Faculty of Life Sciences, Tel Aviv University, Tel Aviv 6997801, Israel

**Keywords:** mutation rate, non-genetic variation, epigenetics, mutation rate phenotype switching, non-genetic inheritance, evolutionary adaptive process

## Abstract

The mutation rate plays an important role in adaptive evolution. It can be modified by mutator and anti-mutator alleles. Recent empirical evidence hints that the mutation rate may vary among genetically identical individuals: evidence from bacteria suggests that the mutation rate can be affected by expression noise of a DNA repair protein and potentially also by translation errors in various proteins. Importantly, this non-genetic variation may be heritable via a transgenerational epigenetic mode of inheritance, giving rise to a mutator phenotype that is independent from mutator alleles. Here, we investigate mathematically how the rate of adaptive evolution is affected by the rate of mutation rate phenotype switching. We model an asexual population with two mutation rate phenotypes, non-mutator and mutator. An offspring may switch from its parental phenotype to the other phenotype. We find that switching rates that correspond to so-far empirically described non-genetic systems of inheritance of the mutation rate lead to higher rates of adaptation on both artificial and natural fitness landscapes. These switching rates can maintain within the same individuals both a mutator phenotype and intermediary mutations, a combination that facilitates adaptation. Moreover, non-genetic inheritance increases the proportion of mutators in the population, which in turn increases the probability of hitchhiking of the mutator phenotype with adaptive mutations. This in turns facilitates the acquisition of additional adaptive mutations. Our results rationalize recently observed noise in the expression of proteins that affect the mutation rate and suggest that non-genetic inheritance of this phenotype may facilitate evolutionary adaptive processes.

## Introduction

### Non-genetic inheritance of a mutator phenotype

Mutators—individuals in a population with an above-average mutation rate—often arise spontaneously during evolution (Wielgoss *et al*. 2013). Mutators may facilitate adaptation as they allow a faster exploration of the space of mutations. Yet, because there are more deleterious than beneficial mutations (Gordo *et al*. 2012), mutators become associated with a higher mutational load compared to non-mutators (Kimura 1967; Kimura and Maruyama 1966). The mutator state is commonly thought to be stably genetically inherited over generations, as a result of, for example, inactivating mutations in DNA mismatch repair genes (Le Bars *et al*. 2013; Matic 1997; Wielgoss *et al*. 2013) or in DNA polymerase genes (Ramiro *et al*. 2020).

In contrast, the possibility of non-genetic inheritance of the mutation rate has received little attention. It has been suggested that transient mutators could arise, for example, as a result of a translation error in DNA repair proteins that could lead to a very strong mutator phenotype (Ninio 1991). This mutator phenotype could be inherited for a few generations via cytoplasmic inheritance of faulty proteins, despite not being encoded in the genotype. In this scenario, an individual's mutation rate can increase for a few generations, generating beneficial mutations without a long-term genetic commitment to an elevated mutation rate and the accumulation of deleterious mutations it entails. Indeed a quantitative analysis has suggested that most adaptive mutations that appear in evolving populations could be due to transient, rather than genetically inherited mutators (Rosenberg *et al*. 1998).

Accumulating empirical evidence suggests that the mutator phenotype can be non-genetically inherited. Translation errors occur in genes involved in DNA repair and replication (Mordret *et al*. 2019), potentially realizing the scenario proposed by (Ninio 1991). Moreover, a mechanism for epigenetic inheritance of the mutation rate in *Escherichia coli* has been described by (Uphoff *et al*. 2016). This mechanism relies on cytoplasmic inheritance of Ada, a DNA repair protein induced under high pH. Each cell carries, on average, a single Ada molecule (Uphoff *et al*. 2016). Therefore, during cell division a substantial proportion of daughter cells inherit zero Ada molecule, and thus experience an elevated mutation rate. Hence, stochastic fluctuations in Ada copy number can affect the mutation rate. Furthermore, *Ada* is positively auto-regulated through a mechanism that can intensify the difference in its active copy number, and hence mutation rate, among genetically identical cells.

A recent study focused on the extreme case where the inheritance of mutation rates between parents and offspring is completely severed. In this context, there exists no discernible relationship between the mutation rates of the parent generation and those of their offspring (Bonhoeffer *et al*. 2016). These authors found that fluctuations in the mutation rate increase the population mean fitness by producing a subpopulation with below-average mutation rate when the population is well-adapted, and a subpopulation with above-average mutation rate when there is a potential for further adaptation. In a subsequent work, it has been suggested that this variation in mutation rate could increase population evolvability (Vincent and Uphoff 2021).

### The inheritance mode of the mutation rate

We suggest that the mode of inheritance of the mutation rate lies on a spectrum: at one end, mutator alleles that arise as rare mutations in genes involved in DNA repair or replication are inherited with very high fidelity, with little to no stochastic effects; on the other end of the spectrum, frequent and stochastic fluctuations in concentrations of proteins that affect the mutation rate may be of such magnitude that there is effectively no correlation between parent and offspring mutation rates; and in the middle of the spectrum, is a range that we define here to be “non-genetic modes of inheritance”, e.g. of the type that can be attained through cytoplasmic inheritance of proteins, or transgenerational epigenetic inheritance of fluctuations in protein concentrations. Such phenomena may produce a partially heritable mutator phenotype that is transmitted across several cellular generations at an intermediate fidelity. Another potential state is aneuploidy, which arises at rates much higher than genetic mutations, and may produce a significant mutator phenotype (Sheltzer *et al*. 2011). This spectrum of inheritance of the mutation rate is a special case of the adaptation spectrum of inheritance (Yona *et al*. 2015), which ranks modes of inheritance from high fidelity, such as genetic inheritance, to very transient, such as physiological changes.

### Overview

Here, we investigate multi-step adaptive evolution with different modes of inheritance of the mutation rate. We have developed a Wright–Fisher model with explicit inheritance of two mutation rate phenotypes: non-mutator and mutator. The main parameters of our model are the switching rate from the non-mutator to the mutator phenotype and the, potentially different, reverse-switching rate from the mutator to the non-mutator phenotype. We also examine several values of the non-mutator mutation rate and the fold-increase in the mutator's mutation rate and explore several fitness landscapes.

We estimate the switching rates for three empirically described systems for non-genetic inheritance of the mutation rate: aneuploidy, the Ada protein in *E. coli,* and cytoplasmic inheritance of mistranslated proteins, and find that these estimates correspond to high adaptation rates on various landscapes.

We suggest that the combinations of switching rates that lead to high adaptation rates fulfil two conditions. First, both switching rates, and especially the switching rate from non-mutator to mutator need to be high enough to ensure a high frequency of mutators at mutation-selection balance, and second, low enough so that the association within the same individuals between the mutator and the mutations it generates is maintained. In this case, individuals that already acquired a portion of the mutations needed towards a high fitness genotype are more likely to keep the mutator phenotype that is needed for the acquisition of the additional missing mutations. Moreover, additional combinations of values for the two switching rates lead to high adaptation rates on smooth landscapes (i.e. without “valleys” and only a single “peak”). These combinations increase the probability of hitchhiking of the mutator phenotype with beneficial mutations, thus facilitating the acquisition of additional beneficial mutations.

## Models and methods

We consider an asexual haploid population with non-overlapping generations and constant population size. We model the effects of mutation, phenotypic switching, natural selection, and genetic drift using a Wright–Fisher (WF) model (Otto and Day 2019). An introduction to the Wright–Fisher model can be found in Supplementary Section a.

In our models, individuals are fully characterized by their mutation rate phenotype, either non-mutator (*m*) or mutator (*M*), and their genotype, which determines their fitness. The genotype is specified differently in each fitness landscape (see below). The combination of these two characteristics is termed pheno-genotype (Feldman and Cavalli-Sforza 1977) to emphasize that it is defined through a combination of a non-genetic and a genetic component, namely the mutator phenotype and genotypic mutations. The frequency of individuals with mutator phenotype *z* and genotype *g* is denoted by $f_{zg}$. In all cases we assume no recombination and, therefore, complete linkage.

Our model is similar to existing models for the study of genetic mutators, which are modifier loci that determine the mutation rate (Ram and Hadany 2012, 2014; Loh *et al*. 2010). However, it is different from other models in that our switching rates can be orders of magnitude higher compared to the mutation rate that generates genetic mutators (Ninio 1991). Moreover, studies on the evolution of genetic mutators often neglect back-mutations from mutators to non-mutators (Desai and Fisher 2011; Ram and Hadany 2012; Ram *et al*. 2018). However, in our model, we consider non-genetic mechanisms for the inheritance of the mutation rate that may exhibit high rates of reversibility.

### Phenotype switching

In every generation, an individual may switch its mutation rate phenotype from non-mutator to mutator with probability $\gamma_{1}$ or from mutator to non-mutator with probability $\gamma_{2}$. We call these parameters “switching rates”. Thus, the pheno-genotype frequencies after phenotype switching are given by


(1) $$\begin{array}{rl} & {{\;f}^{\prime }}_{mg}=(1-\gamma_{1})\cdot f_{mg}+\gamma_{2}\cdot f_{Mg}\\ & {{\;f}^{\prime }}_{Mg}=\gamma_{1}\cdot f_{mg}+(1-\gamma_{2})\cdot f_{Mg}. \end{array}$$

**Fig. 1.**
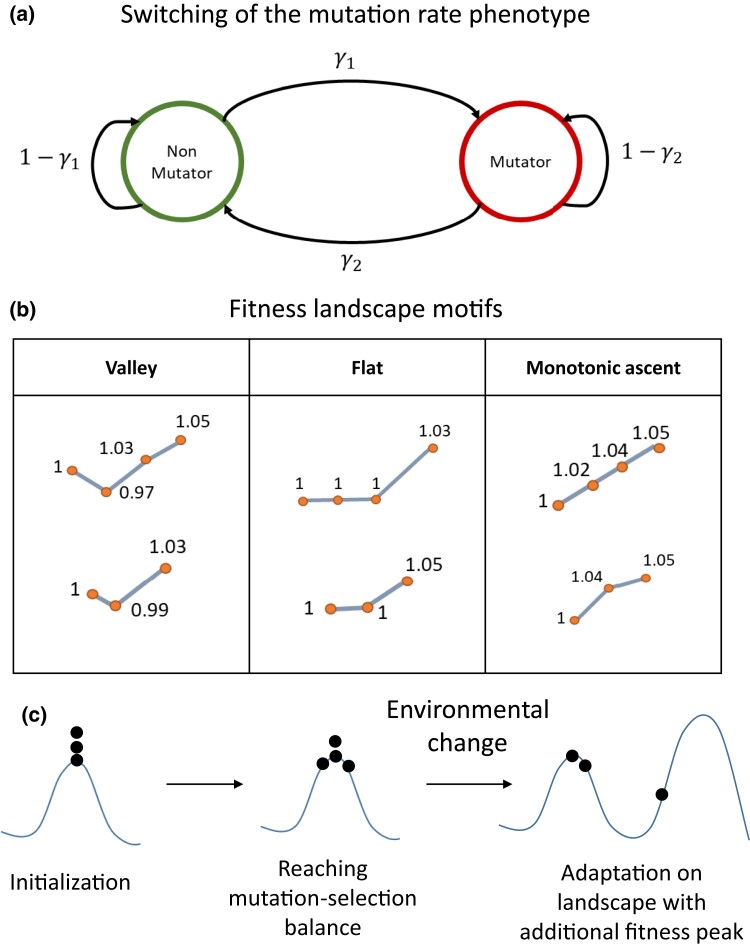
a) Switching between the non-mutator and the mutator phenotypes. The switching rate from non-mutator to the mutator phenotype $\gamma_{1}$ is the probability of an individual having a mutator phenotype given that its parent was a non-mutator. The switching rate from mutator to non-mutator $\gamma_{2}$ is the probability of an individual having a non-mutator phenotype given that its parent was a mutator. b) Fitness landscape motif examples. A fitness landscape motif is a succession of genotypes that are one mutation away from each other. The first genotype is considered the wild-type, and has a relative fitness of 1. The genotype with the highest number of mutations always has the highest fitness and is the adaptive genotype. Depending on the fitness of the intermediate genotypes, a fitness motif can be classified as valley, flat, or monotonic ascent. c) Analysis workflow. The population is initialized at the wild-type genotype. The population is then allowed to reach mutation-selection balance on a fitness landscape where the wild-type genotype has the highest fitness. Next, an environmental change occurs. A genotype that is different from the wild-type genotype now has the highest fitness.

In this equation, $f_{zg}$ is the frequency of a given genotype *g* before switching of the mutator phenotype and *m* or *M* are for non-mutator and mutator phenotypes, respectively. The frequency after switching is ${\;f}_{zg}^{\prime }$. A schematic representation of the switching is shown in Fig. 1a. Later, we also extend our model to include a third transition pheno-genotype with a mutation rate that is intermediate between the non-mutator and the mutator mutation rates (see Supplementary Fig. 1).

### Mutation

The pheno-genotype frequencies after mutation, ${\;f}_{zg}^{\prime \prime }$, are given by


(2) $$\begin{array}{rl} & {{\;f}^{\prime \prime }}_{mg}=\underset{j}{\sum }\;u_{\;j\to g}{{\;f}^{\prime }}_{mj}\\ & {{\;f}^{\prime \prime }}_{Mg}=\underset{j}{\sum }\;{\tilde{u}}_{\;j\to g}{{\;f}^{\prime }}_{Mj}, \end{array}$$

where ${\;f}_{zj}^{\prime }$ is the frequency before mutation, *j* is an index over all possible genotypes, *m* or *M* are for non-mutator and mutator phenotypes, respectively. For non-mutators, $u_{\;j\to g}$ is the mutation transition probability from genotype *j* to genotype *g*. For mutators, ${\tilde{u}}_{\;j\to g}$ is the mutation transition probability from genome *j* to genotype *g*. The specific transition probabilities $u_{\;j\to g}$ and ${\tilde{u}}_{\;j\to g}$ in each fitness landscape are given below.

### Selection

In accordance with previous work on the evolution of modifiers, we assume that the mutator phenotype is neutral (James and Jain 2016; Desai and Fisher 2011; Liberman and Feldman 1986), that is, it does not directly affect fitness. In contrast to most previous work, we do not assume it is a gene, i.e. we do not assume genetic inheritance. Hence, we can focus on the fitness $w_{g}$ of genotype *g*, rather than the fitness of pheno-genotypes. The specific fitness values are determined by the fitness landscape, see below. The pheno-genotype frequencies after selection are given by


(3) $${\;f}_{zg}^{\prime \prime \prime }=\frac{w_{g}}{\bar{w}}\cdot {\;f}_{zg}^{\prime \prime },$$

where $\bar{w}$ is the population mean fitness, $\bar{w}=\underset{g}{\sum }\;w_{g}({{\;f}^{\prime \prime }}_{mg}+{{\;f}^{\prime \prime }}_{Mg})$.

The fitness landscapes determining the fitness assigned to each genotype are detailed in the next section. In a first phase, the population first reaches mutation-selection balance on a fitness landscape where the wild-type genotype has the highest fitness. Then, in a second phase, the fitness landscape, and therefore the fitness $w_{g}$ change, modelling an environmental change. We analyse the effect of non-genetic mutation rate inheritance on the rate of adaptation to the second phase, as populations depart from mutation-selection balance in the first phase.

### Genetic drift

We model the effect of random genetic drift by drawing the number of individuals of each pheno-genotype from a multinomial distribution parameterized by the population size *N* and the pheno-genotype frequencies after mutation, phenotype switching, and selection, ${\;f}_{zg}^{\prime \prime \prime }$.

### Analysis

We study the adaptation of a population following an environmental change. In the first phase, the population is situated in an environment to which it is well-adapted. The wild-type genotype has the highest fitness. All other genotypes are maladapted, and hence the population converges to a mutation-selection balance around the wild-type genotype. We initialize this phase by considering a population composed of only non-mutators with an initial genotype. We neglect the effects of drift. Hence, pheno-genotype frequencies are only affected by mutation, phenotype switching, and selection. We iterate Eqs. (1–3) until the population reaches an equilibrium (that is, the frequencies of the pheno-genotypes do not change from generation to generation, ${\;f}_{zg}^{\prime \prime \prime }=f_{zg}$). The equilibrium frequencies can also be obtained by numerically solving for the eigenvalues of the mutation-selection transition matrix, and normalizing the eigenvector corresponding to the leading eigenvalue (Otto and Day 2019). We observed that the values obtained by solving the eigenvalue problem corresponded closely to the values obtained from a numerical iteration of the model. We thus obtain a vector of frequencies of each pheno-genotype at mutation-selection balance.

In the second phase, the population has been subject to an environmental change. The initial population corresponds to the mutation-selection balance frequencies obtained in the first phase. A different genotype now has the highest fitness. We no longer neglect the effects of drift. The model is now simulated by iteration of Eqs. (1–3) and random sampling at each generation using a multinomial distribution.

The flow of our analysis is presented in Fig. 1c. The definitions of all genotypes' fitness before the environmental change (first phase) and after the environmental change (second phase) are specified in the following section.

## Fitness landscapes

### Simple fitness landscape

To simplify our analysis before moving into biologically realistic fitness landscapes, we first study the rate of adaptation for a range of switching rates for a simple fitness landscape with a single adaptive peak.

#### Genotype

We consider a genome composed of two or three environment-specific loci that affect fitness differently depending on the environment, and a large number of background loci, in which all mutations are deleterious. Thus, the genotype is denoted by the number of mutations in the environment-specific loci and by the number of deleterious mutations accumulated in the background genomic loci. For example, the wild type genotype is *0\0*: it carries zero mutation in the major environment-specific loci and zero mutation in the background loci, while a genotype *2\3* has two mutations in the major environment-specific loci and three mutations in the background loci. The frequency of the genotype *0\0* is noted $f_{m,0\setminus 0}$ for non-mutators and $f_{M,0\setminus 0}$ for mutators (see above).

#### Fitness before the environmental change

During this phase, the wild-type genotype has the highest fitness: $w_{0\setminus 0}=1$. We assume that all mutant alleles in both the major and the background loci are deleterious with a selection coefficient *s* per mutation, such that the multiplicative fitness effect of *k* mutant alleles is $(1-s{)}^{k}$. For example, the fitness of the genotype 2\3 with two mutant alleles in the major loci and three mutant alleles in the background loci is $w_{2\setminus 3}=(1-s{)}^{5}$.

#### Fitness after the environmental change

The double mutant in the environment-specific loci (when considering two environment-specific loci) or the triple mutant in the major loci (when considering three environment-specific loci) has the highest fitness. We still have $w_{0\setminus 0}=1$. We define the vector $\left(w_{1\setminus 0},w_{2\setminus 0}\right)$ that describes the fitness values of individuals with one and two mutant alleles in the environment-specific loci and zero mutant allele in the background loci; when considering three environment-specific loci, we use the vector $\left(w_{1\setminus 0},w_{2\setminus 0},w_{3\setminus 0}\right)$. Mutations in background loci still have a deleterious multiplicative effect. Thus, for example, the fitness of a genotype with two mutant alleles in the major loci and three in the background loci is $w_{2\setminus 3}=w_{2\setminus 0}\cdot (1-s{)}^{3}$. The adaptive genotype is always the genotype with the largest number of mutant alleles in the environment-specific loci. Hence, $w_{2\setminus 0}>w_{1\setminus 0}$ when considering two environment-specific loci and $w_{3\setminus 0}>\max(w_{2\setminus 0},w_{1\setminus 0})$ when considering three environment-specific loci.

#### Fitness landscape motifs

The fitness vectors $\left(w_{1\setminus 0},w_{2\setminus 0}\right)$ and $\left(w_{1\setminus 0},w_{2\setminus 0},w_{3\setminus 0}\right)$ allow us to introduce “fitness landscape motifs”. Note that in the notation $w_{a\setminus b}$, only *a* relates to fitness landscape motifs, since only *a* relates to environment-specific loci. Fitness landscape motifs are a succession of mutant genotypes and their fitness. The wild-type fitness is $w_{0\setminus 0}$. For example, if $w_{1\setminus 0}<w_{0\setminus 0}<w_{2\setminus 0}$ when considering two major loci, the fitness motif will feature a fitness valley. If $w_{0\setminus 0}<w_{1\setminus 0}<w_{2\setminus 0}$, the fitness motif is a monotonic ascent (see Fig. 1b). Biological landscapes are composed of many such fitness landscape motifs, similarly to biological networks that feature network motifs (Shoval and Alon 2010). We explore below how different combinations of switching rates influence adaptation over various fitness landscape motifs (see Fig. 1b). For a discussion of the relationship between models of fitness landscapes and biological fitness landscapes: Supplementary Section b.

#### Mutation

The number of mutations per generation that occur at the background loci is Poisson distributed with expected value *U* or *τU* in individuals with non-mutator or mutator phenotype, respectively. Therefore, a genotype with *k* deleterious mutations will mutate to have *k + l* deleterious mutations with probability $e^{-U}U^{l}/l!$ or $e^{-\tau U}(\tau U{)}^{l}/l!$ in individuals with non-mutator or mutator phenotype, respectively (*l* is assumed to be positive as we assume no back mutations). Mutations occur at the major loci with probability *μ = U/n* or *μ = τU/n*, according to the phenotype, where *n* is the total number of loci in the genome and *μ* is the per-locus mutation rate. Note that *U* represents the mutation rate only at the genomic loci; since we consider few environment-specific loci, this background mutation rate is approximately equal to the total mutation rate.

The number of mutations in the major loci is binomially distributed. The parameters of the binomial distribution are the number of major loci and the per locus mutation rate. We neglect the effects of back mutations, both in the numerical model and the stochastic model. This is because of the probability of a back-mutation following a mutation is negligible, as both of these events are rare. See Supplementary Section c for a formal description of the mutation transition probabilities.

### Complex and empirical fitness landscapes

We explore several fitness landscapes: NK landscapes with various ruggedness, commonly used to model biological fitness landscapes (Kauffman and Weinberger 1989; Obolski *et al*. 2018), and an empirical landscape derived from the fungus *Aspergillus niger*, established by (de Visser *et al*. 2009; de Visser and Krug 2014). A description of these landscapes and the choice of wild-type genotypes can be found in the Supplementary Section d.

## Values for model parameters

### Mutation rates

The size of the *Saccharomyces cerevisiae* genome is $1.2\cdot 10^{7}$ bp (“Saccharomyces Cerevisiae (ID 15)—Genome—NCBI” n.d.), while the size of the *Escherichia coli* genome is about $5\cdot 10^{6}$ bp (“Escherichia Coli (ID 167)—Genome—NCBI” n.d.). The proportion of deleterious mutations, out of all mutations, is about 40% (Gordo *et al*. 2012; Eyre-Walker and Keightley 2007). The mutation rate per bp for *S. cerevisiae* is about $10^{-10}$ per bp (Lang and Murray 2008); for *E. coli*, it is of the order of $10^{-9}-10^{-10}$ per bp (Lee *et al*. 2012; Jee *et al*. 2016). Hence, we explore deleterious mutation rates from $4\cdot 10^{-5}$ to $10^{-3}$ per genome per cell cycle.

### Mutation rate phenotype switching rates

The two main parameters of our model are the switching rate from non-mutator to mutator $\gamma_{1}$ and the reverse switching rate from mutator to non-mutator $\gamma_{2}$. As a special case the two rates may be identical. At least three potential systems for non-genetic inheritance of the mutation rate have been described in the literature: the Ada protein in *E. coli*; the cytoplasmic inheritance of mistranslated faulty proteins that affect mutation rate; and aneuploidy. For each of these, we estimate an approximate value for $\gamma_{1}$ and $\gamma_{2}$. Depending on the considered system, the two switching rates are not independent of each other. We therefore introduce a variable α such that $\gamma_{1}=\alpha \;\gamma_{2}$.

#### Ada protein in *E. coli*

The Ada protein is involved in DNA repair under high pH. The number of Ada molecules per cell is Poisson-distributed with an average of ∼1 (Uphoff *et al*. 2016). Cells with zero Ada molecule cannot efficiently trigger DNA repair under high pH and hence function as potential mutators.

On average, cells with zero Ada molecule produce one Ada molecule per cell cycle or generation (Uphoff *et al*. 2016). We assume that the production of Ada molecule in a population of cells with zero Ada molecule follows a Poisson process with a rate of one molecule per generation. Thus, the probability of transitioning from mutator to non-mutator phenotype is *1-e*∼0.63. We assume that stationary frequency of cells with zero Ada molecule is 25% (reported 20–30% in [Uphoff *et al*. 2016]). Solving for the probability of transition from cells with non-zero Ada molecule to cells with zero Ada molecule, we estimate this probability to be 0.21 (see Supplementary Section e). Hence, for the Ada system of inheritance of the mutation rate, we have $\gamma_{1}=0.21$ and $\gamma_{2}=0.63$. For this system, we have α∼1/3.

#### Aneuploidy

The state of aneuploidy—an abnormal number of chromosomes in the cell—has been shown to be associated with higher mutation rate (Sheltzer *et al*. 2011). Aneuploidy also comes with a fitness disadvantage, although in specific environments, some aneuploidies have a large fitness advantage (Yang *et al*. 2021; Yona *et al*. 2012). In this paper, and for simplicity, we consider aneuploidy to be neutral.

The rate of aneuploidy in yeast has been estimated at $10^{-4}$ for whole-chromosome duplication and at $10^{-5}$ for whole-chromosome loss (Zhu *et al*. 2014; Gilchrist and Stelkens 2019). Other estimates are $6.7\cdot 10^{-6}$ chromosome duplication events per generation and $3\cdot 10^{-5}$ chromosome loss events per generation (Kumaran *et al*. 2013). Whole-chromosome loss in aneuploids is potentially faster than in euploids (Sheltzer *et al*. 2011; Ippolito *et al*. 2021). Hence, we estimate $\gamma_{1}=10^{-4}$ and $\gamma_{2}=10^{-5}$. For this system, we have α∼10.

#### Cytoplasmic inheritance of mistranslated proteins

We consider a hypothetical protein involved in DNA repair. We assume that this protein has the average length of a protein, is present in a single molecule in each cell, and is synthetized only when diluted out. In this section, we will compute the rate of transgenerational loss-of-function of that protein due to mistranslation and cytoplasmic inheritance. The assumption of a single molecule in each cell is made for simplicity, as it avoids the arbitrary decision of determining how many functional protein copies are needed for a non-mutator phenotype.

In *S. cerevisiae*, the rate of mistranslation is $10^{-3}$ per codon (Kramer *et al*. 2010) and the average length of a eukaryotic protein is about 500 aminoacids (Tiessen *et al*. 2012). Following estimations from an empirical fitness landscape (Sarkisyan *et al*. 2016), we assume that about 10% of point mutations abolish the function of the protein. Hence, assuming a mistranslation rate of $10^{-4}$ per codon, we have $\gamma_{1}=1-(1-10^{-3}{)}^{50}=0.05$. The switching rate from mutator to non-mutator is $\gamma_{2}=0.5\cdot (1-10^{-3}{)}^{50}=0.476∼0.5$.

The rate of amino acid substitution in *E. coli* is about $10^{-4}$ to $10^{-3}$ per codon (Kramer and Farabaugh 2007; Mordret *et al*. 2019). The average length of a protein in bacteria is about 300 aminoacids (Tiessen *et al*. 2012). Hence, the switching rate from non-mutator to mutator, assuming a mistranslation rate of $5\cdot 10^{-4}$ (Kramer and Farabaugh 2007; Mordret *et al*. 2019), is $\gamma_{1}=1-(1-5\cdot 10^{-4}{)}^{30}$ = 0.0149. The switching rate from mutator to non-mutator would be the dilution rate, that is 0.5, multiplied by the probability that the new cell will synthetize a functional protein. Hence, $\gamma_{2}\;∼\;0.5$. For this system, we have α∼1/36.

A summary of the estimates for the two switching rates and other model parameters is in Table 1 and Table 2. In this study, we use $10^{-6}$ as a low bound on genetic inheritance of the mutation rate. Because we consider two mutation rate phenotypes, a switching rate of 0.5 indicates that the mutation phenotype of the offspring is independent from the mutation rate phenotype of its parent. Switching rates higher than 0.5 signify that the offspring is more likely to have the mutation rate phenotype opposite of that of its parent than to have the same mutation rate phenotype.

**Table 1.** Summary of model parameters.

Symbol	Name	Estimate	References
N	Population size	10^5^–10^8^	Berg (1996)
U	Genome-wide mutation rate	0.0004–0.003	Wielgoss *et al*. (2011)
*n*	Number of loci	4,000–5,000	Serres *et al*. (2001)
μ	Per-locus mutation rate	U/n	
τ	Fold-Increase in mutator mutation rate	10–1,000	Hall and Henderson-Begg (2006), Tenaillon *et al*. (1999), Desai and Fisher (2011)
s	Selection coefficient against deleterious mutations	0.001–0.03	Gordo *et al*. (2012)
*γ*	Mutator phenotype switching rate	10^−6^−0.5	Ninio (1991), Desai and Fisher (2011)
*δ*	Switching rate from transition mutation rate phenotype	0–1	

Adapted from (Ram and Hadany 2014).

**Table 2.** Estimates of switching rates $\gamma_{1}$ and $\gamma_{2}$ for three considered mechanisms for non-genetic inheritance of the mutation rate.

Switching rate	Estimate	References
***Ada* protein**		
Non-mutator to mutator $\gamma_{1}$	0.21	Uphoff *et al*. (2016)
Mutator to non-mutator $\gamma_{2}$	0.63	
**Aneuploidy**		
Non-mutator to mutator $\gamma_{1}$	$10^{-4}$	Sheltzer *et al*. (2011), Zhu *et al*. (2014), Gilchrist and Stelkens (2019), Kumaran, Yang, and Leu (2013), Ippolito *et al*. (2021)
Mutator to non-mutator $\gamma_{2}$	$10^{-5}$	
**Cytoplasmic inheritance of mistranslated proteins**		
Non-mutator to mutator $\gamma_{1}$	0.014	Kramer and Farabaugh (2007), Mordret *et al*. (2019), Tiessen *et al*. (2012), Sarkisyan *et al*. (2016), Kramer *et al*. (2010)
Mutator to non-mutator $\gamma_{2}$	0.5	

### Fold-increase in the mutation rate in mutators

Most deletion mutants for genes involved in DNA repair in *S. cerevisiae* exhibit increase in mutation rate from 2- to 200-fold (Serero *et al*. 2014). Mutator strains in *E. coli* exhibit a 50- to 1000-fold increase in mutation rate (Kang *et al*. 2019). In this paper, we explore a fold increase in mutation rate for a mutator, τ=10 and τ=100, which is in the range of empirically observed values for the two microbes. We also estimate the fold-increase in mutator mutation rate for each of the three empirically described systems of non-genetic inheritance of the mutation rate.

#### Ada protein in *E. coli*

The fold-increase in a cell with no Ada molecule can be approximated by the fold-increase in the mismatch rate in Δada cells with regards to cells with intact Ada. According to (Uphoff 2018), we have τ≈3.

#### Aneuploidy

The fold-increase in mutation in a *S. cerevisiae* cell with additional chromosomes ranges from 0.7 (for ChrXVI) to 4.1 (for ChrXIV) (Sheltzer *et al*. 2011). We consider τ≈2.

#### Mistranslation

The fold-increase in the mutation rate due to the synthesis of defective DNA polymerases can be approximated by the fold-increase in the mutation rate of mutants in the DNA polymerase gene. According to (Loh *et al*. 2010), this fold-increase can reach up to a 1000-fold. However, most mutants exhibit increases in the range of 10–100-fold. We consider τ≈100.

## Results

### The adaptation rate is mostly determined by the frequency of the mutator mutant at mutation-selection balance

We first consider a population at mutation-selection balance around the wild-type genotype. That is, at mutation-selection balance the population is well-adapted to its environment and the frequencies of the pheno-genotypes do not change from generation to generation $\left({{\;f}^{\prime \prime \prime }}_{zg}=f_{zg}\right)$ due to a balance between mutation and phenotype switching, which generate genetic and phenotypic variation, and selection and drift, which eliminate variation.

We focus on four pheno-genotypes (their frequencies appear in parentheses): non-mutators with wild type genotype (*m_0_*); non-mutators mutants (*m_1_*); mutators with wild type genotypes (*M_0_*) and mutators mutants (*M_1_*). Note that $f_{m,0\setminus 0}=m_{0}$, $f_{m,1\setminus 0}=m_{1}$, $f_{M,0\setminus 0}=M_{0}$, and $f_{M,1\setminus 0}=M_{1}$. Note that we consider the genotype 2\0 to be adaptive. Hence, the mutants m,1∖0 and M,1∖0 correspond to mutants that already have one out of the two mutations needed to become well-adapted. These four frequencies are plotted for s=0.1, $U=4\cdot 10^{-5}$ and τ=100 in Supplementary Fig. 2. Note that since the population is well-adapted to its environment, all mutants are deleterious. It is sufficient to focus on the four major genotypes due to our assumption on the population size (see Supplementary Section f), and following (Dawson 1998). In short, we assume that the population is large enough to contain single mutant, but small enough so it does not contain the double, or triple mutant genotype that will become adaptive to the new environment. We also define $p_{M}$ as the frequency of mutators (as a sum of mutators with and without the mutation) in the population and $p_{S}$ the frequency of mutants (sum of mutator and non mutator single mutants in the environment-specific loci) in the population.

Adaptation is the appearance and subsequent fixation of the 2\0 genotype. We denote by *q* the probability of appearance and by π the probability of fixation conditional on appearance.

The probability that in the next generation no 2\0 mutant appears and is destined for future fixation in a population of size N is $(1-q\pi {)}^{N}$. Assuming the number of generations until the appearance of a double mutant that goes to fixation is geometrically distributed, the adaptation rate ν is defined as the probability that a single double mutant appears and escapes extinction by genetic drift,


(4) $$\nu =1-(1-q\pi {)}^{N}\approx Nq\pi$$

where the approximation holds when Nqπ is small. Note that the switching rates $\gamma_{1}$ and $\gamma_{2}$ affect the adaptation rate only indirectly due to their role in the mutation-selection balance (also referred to as: MSB) frequencies $m_{0}$, $m_{1}$, $M_{0}$, and $M_{1}$. We show in Supplementary Section g, that the probability of fixation of the double mutant given that it appeared, π, does not depend on the switching rate (see Supplementary Fig. 3). Indeed, it is only dependent on its fitness, equal to 1+sH, where *H* is the adaptation coefficient. Also, the time to fixation does not depend on the switching rate (see Supplementary Fig. 4). Hence, in order to understand how the switching rates affect the rate of adaptation, we can focus on the probability of appearance of the double mutant. Indeed, given that the mutation rates are low, one does not expect fixation probabilities and times to be significantly affected by recurrent mutation.

The probability of appearance of the adaptive double mutant is derived from the frequencies $m_{0}$, $m_{1}$, $M_{0}$, and $M_{1}$. We neglect the frequency of a double mutant at the MSB (i.e. we disregard terms of order *μ*^2^) due to our assumption on the population size and the mutation rate (that is, $\frac{s}{\mu }e^{U/s}<N<{\left(\frac{s}{\tau \mu }\right)}^{2}e^{\frac{\tau U}{s}}$, see Supplementary Section f). The probability *q* of appearance of double mutants as a result of a mutation in an existing single mutant in a population that currently does not have any double mutants is the sum of the probabilities of its appearance either from a mutator or a non-mutator


(5) $$q=\mu \;m_{1}\exp\;(-U)+\;\tau \mu \;M_{1}\;\exp(-\tau U).$$

Here, *q* is dominated by the term $\tau \mu \;M_{1}$, i.e. appearance from a mutator and therefore maximization of $M_{1}$ will also lead to maximization of the adaptation rate. Hence, the frequency $M_{1}$ of the mutator mutant is of crucial importance to the rate of adaptation.

In this section, we have shown that there exists a linear relationship between the probability of appearance of the adaptive genotype and the rate of adaptation. Therefore, the switching rates that maximize the probability of appearance of the adaptive genotype also maximize the rate of adaptation, and hence the probability of appearance of the adaptive genotype can be used as a proxy for the rate of adaptation. Moreover, the probability of appearance of the adaptive genotype is dominated by the frequency of mutators with pre-existing variation at the environment-specific loci.

### The adaptation rate is maximized for intermediate switching rates

**Fig. 2.**
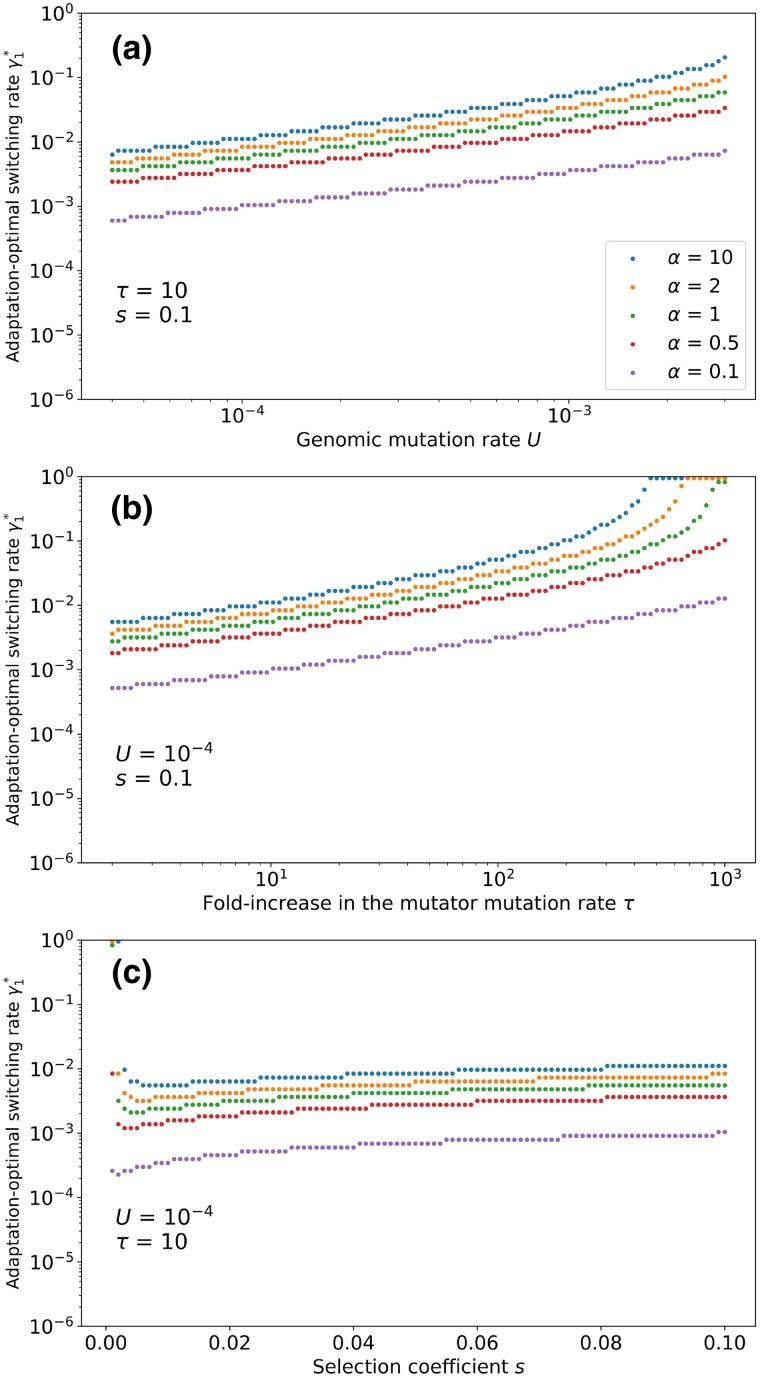
Adaptation-optimal switching rate $\gamma_{1}^{*}$ for varying values of U, τ, s. For each combination of parameters, we calculate the probability of appearance of the adaptive mutant for $\gamma_{1}$ ranging from $\gamma_{1}=\;10^{-6}$ to $\gamma_{1}=0.5$, and the value resulting in the higher rate of adaptation is recorded. Other parameters: $n=5000;\;\gamma_{2}=\alpha \;\gamma_{1}$.

What are the switching rates that maximize the probability of appearance of the adaptive genotype, and hence the rate of adaptation? The three major parameters of our model are the genomic mutation rate *U*, the fold-increase in the mutator mutation rate τ and the selection coefficient *s*. We define the adaptation-optimal switching rate, denoted by $\gamma_{1}^{*}$, as the switching rate from non-mutator to mutator that maximises the rate of appearance of the adaptive genotype. For each of the three parameters, we computed numerically the adaptation-optimal switching rate for a range of values for each parameter while the other two were held constant. We iterated our model until reaching mutation-selection balance, then used the frequency of non-mutator mutant $m_{1}$ and the frequency of mutator mutant $M_{1}$ to compute the probability of appearance of the adaptive genotype according to Eq. 5 for a range of switching rates. The switching rate that maximizes the probability of appearance of the adaptive genotype is the adaptation-optimal switching rate. The switching rate from mutator to non-mutator $\gamma_{2}$ was set such that $\gamma_{1}=\alpha \;\gamma_{2}$, and values of α ranging from 0.1 to 10 were explored. The results are shown in Fig. 2.

We found that the adaptation-optimal switching rate increases with the genomic mutation rate and the fold-increase in the mutator mutation rate. It also increases with the value of α. Increasing α means that the switching rate from non-mutator to mutator becomes higher compared to the switching rate from mutator to non-mutator. The selection coefficient *s* has a minor effect on the adaptation-optimal switching rate. For all considered parameters, the adaptation-optimal switching rates are higher than $5\cdot 10^{-4}$. For high values of τU, the adaptation-optimal switching rates could exceed $\gamma_{1}=0.5$, which corresponds to no inheritance of the mutation rate phenotype, that is equal probability of inheriting or not the parental phenotype. Note that $\gamma_{1}>0.5$, corresponds to a theoretical situation where offspring are less likely to follow their parental phenotype than to follow it. We term this situation “contrarian inheritance”. We see on Fig. 2 that contrarian inheritance maximizes the probability of appearance for τ>500.

We then sought to determine what properties of the population at mutation-selection balance result in high probability of appearance of the adaptive genotype. As mentioned in the previous section, the frequency of $M_{1}$ dominates the expression for *q*. Hence, maximizing the frequency of mutator mutants at mutation-selection balance will also maximize the probability of appearance *q*, and therefore the rate of adaptation.

### The advantage of intermediate switching rates stems from high frequency of mutators and high association between the mutator and mutant

**Fig. 3.**
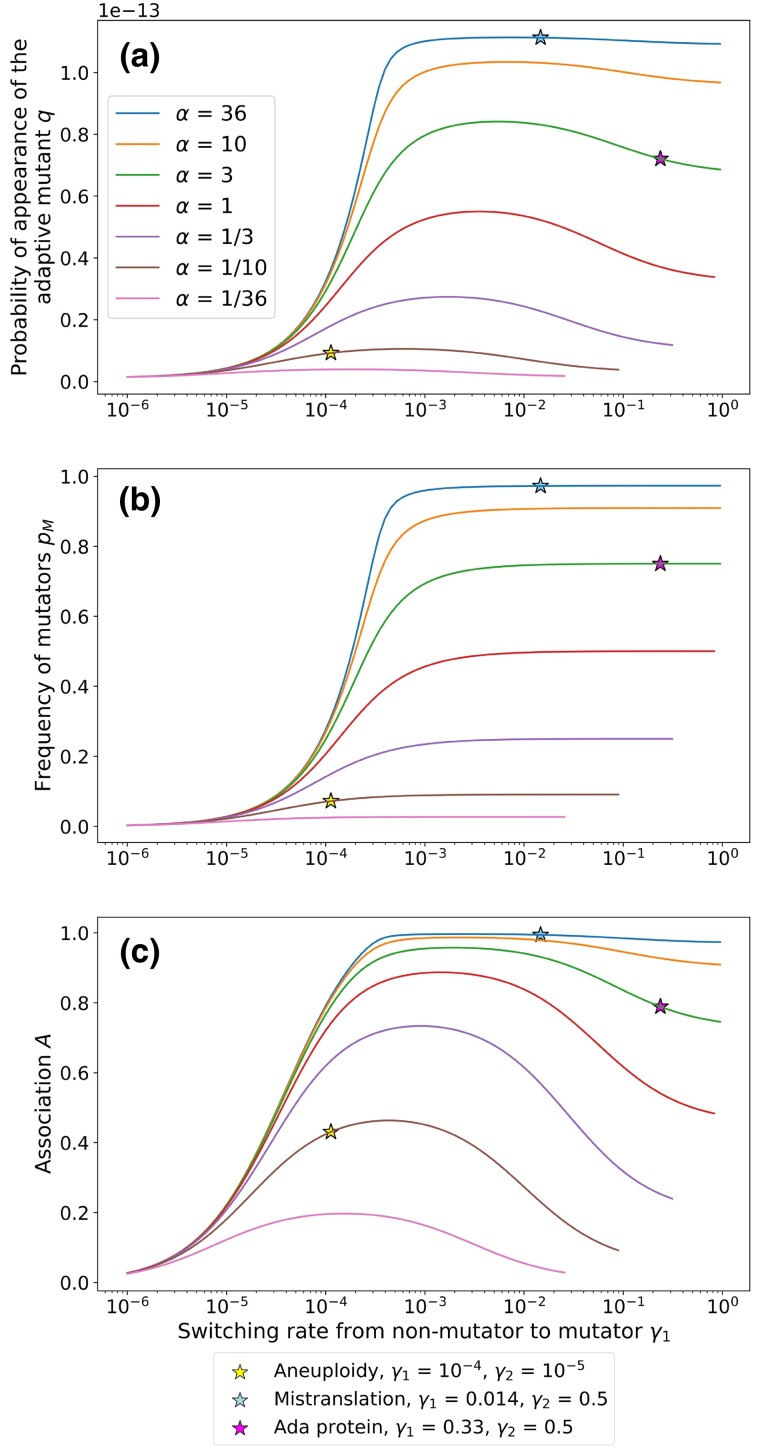
The probability of appearance of the adaptive genotype is maximized for intermediate switching rates. The frequency of mutators $p_{M}$ increases with the switching rate, while the association between mutants and the mutator phenotype is maximized for intermediate switching rates. The probability of appearance of the adaptive genotype *q* is computed with Eq. 5 and MSB frequencies obtained numerically for each value of $\gamma_{1}$ and other parameters. The frequency of mutators $p_{M}$ is obtained directly from the MSB frequencies. The association *A* is computed from the MSB frequencies using Eq. 8. Parameters: n=5000, $\gamma_{2}=\alpha \;\gamma_{1}$.

We plotted the probability of appearance of the adaptive mutant *q* along the switching rate from non-mutator to mutator $\gamma_{1}$ in Fig. 3. We also indicated, with coloured stars, the location in the parameter space of the three empirically described systems for non-genetic inheritance of the mutation rate. For each of the three systems for non-genetic inheritance of the mutation rate, we also plotted the individual curves along $\gamma_{1}$ in Supplementary Fig. 5. We notice that the switching rate from non-mutator to mutator that we estimated for each system is also the switching rate that leads to high probability of appearance of the adaptive mutant.

We postulated two factors that can increase the frequency of $M_{1}$ at mutation-selection balance, and therefore the adaptation rate: (1) because mutators are more likely than non-mutators to generate mutations, any effect that will increase the proportion of mutators $p_{M}$ at mutation-selection balance will increase the frequency of mutant mutators $M_{1}$; and (2) maintaining the association between mutators and the mutants it generates, after they are generated. Below we explore each of these two effects.

### Frequency of mutators at mutation-selection balance

We approximate the frequency of mutators at mutation-selection balance (MSB). We denote by $p_{M}^{-}$ the frequency of mutators at MSB when $\gamma_{2}$ is low and by $p_{M}^{+}$ the frequency of mutators at MSB when $\gamma_{2}$ is high.

Here, we follow (Desai and Fisher 2011), which studies the frequency of genetic mutators at mutation-selection balance. Therefore, they assumed low $\gamma_{1}$ and neglected $\gamma_{2}$ altogether. They found that


(6) $$p_{M}^{-}=\;\frac{\gamma_{1}}{\;(\tau -1)U}\approx \frac{\gamma_{1}}{\tau \;U}\;.$$

Equation 4 provides a good approximation of the frequency of mutators $p_{M}$ at mutation-selection balance when $\gamma_{2}$ is low. When $\gamma_{2}$ increases, the approximation becomes worse (Supplementary Fig. 6). Above $\gamma_{2}>\tau U/2$, the balance between the two mutation rate phenotypes is no longer expressed by Eq. 4.

When both switching rates are high, $\gamma_{1},\gamma_{2}>\tau U/2$, the indirect selection against the mutator phenotype becomes inefficient. Thus, the mutation-selection balance between the non-mutator and the mutator phenotypes can be approximated by the stationary distribution of a Markov chain, such that $p_{M}^{+}$, the frequency of mutators with high switching, is


(7) $$p_{M}^{+}=\;\frac{\gamma_{1}}{\gamma_{1}+\gamma_{2}}.$$

From Fig. 3, we observe a correlation between the proportion of mutators $p_{M}$ and the rate of adaptation. This suggests that the rate of adaptation will be always higher for $\gamma_{1}>5\cdot 10^{-4}$. This is also in agreement with our results from Fig. 2. The frequency of mutators at MSB explains why low switching rates, corresponding to genetic inheritance of the mutation rate, result in low rates of adaptation.

Therefore, in the first regime (Eq. 6), the frequency of mutators at MSB depends on both mutation and selection. The numerator of Eq. 6 is the rate at which mutators are generated, whereas the denominator is the fitness disadvantage, i.e. the increase in load caused by the mutator. In the second regime (Eq. 7), the switching rates are stronger than selection against mutators, and selection can be neglected.

### Association between mutator and mutant phenotypes

Maximising the frequency of mutators is not sufficient to maximise the frequency of mutator mutants. The frequency of the mutator phenotype is not independent from the mutations it generates: a large fraction of mutations are generated by mutators (Desai and Fisher 2011; Ninio 1991), and inheritance of the mutator phenotype preserves the association between mutators with mutants.

The association between the mutator and the mutant phenotypes can be quantified by


(8) $$A=M_{1}-p_{M}\cdot p_{S},$$

which is defined as the association between the mutator phenotype and the single mutant genotypes minus their expected frequency that would be expected if they were independent, that is given by their frequency product. Thus, if the mutator and mutant state were independent then A=0. When $\gamma_{1}>5\cdot 10^{-2}$, the probability of appearance decreases from its maximum value, proportionately to the decrease in association (see Fig. 3c). The decreasing association between mutator and mutant for higher switching $\gamma_{1}$ explains the corresponding decrease in the probability of appearance of the adaptive genotype. The association between mutator and mutant is crucially dependent on the switching rate from mutator to non-mutator $\gamma_{2}$, which increases with $\gamma_{1}$ for a fixed α.

Therefore, we conclude that two properties of the MSB population lead to high probability of appearance of the adaptive genotype, and therefore to a high rate of adaptation. The first is a high frequency of mutators, which is achieved for $\gamma_{1}>\tau U/2$ (see above). This is an intuitive result: a higher frequency of mutators increases the probability of appearance of the adaptive genotype, and it is achieved when the switching rate from non-mutator to mutator is high. However, this property is not sufficient to maximize the probability of adaptation. The second property of the population at MSB to maximize the probability of adaptation is a high association between the mutator and mutant, which decreases when $\gamma_{1}>5\cdot 10^{-2}$.

Lastly, we wanted to explore the probability of appearance of the adaptive genotype for extreme values of our major parameters: the genomic mutation rate *U* ranging from $4\cdot 10^{-5}$ to $3\cdot 10^{-3}$; the fold-increase in mutator mutation rate τ ranging from 2 to 1,000; and the selection coefficient *s* ranging from 0.001 to 0.1. In Supplementary Fig. 7, we observe that the probability of appearance of the adaptive genotype was always very low for $\gamma_{1}<5\cdot 10^{-4}$, which confirmed our conclusions from Fig. 2. For high τ and low *s*, as well as high *U*, high τ and high *s*, switching rates that are higher than 0.5 result in a much lower probability of appearance of the adaptive genotype than the maximum. For all other parameter combinations, the maximum probability of appearance of the adaptive genotype *q* is roughly to its value at $\gamma_{1}=0.95$. A sensitivity analysis revealed that the ratio of the mutation rate in the environment-specific to the mutation rate in the background loci has little influence on the rate of adaptation (see Supplementary Fig. 8). Next, we examined the influence of the deleterious mutation rate on the adaptation-optimal switching rate. We vary the deleterious mutation rate without changing the mutation rate at environment-specific loci. We explore scenarios where the deleterious mutation rate is 50-fold lower or 50-fold higher than in the main model. We found that the adaptation-optimal switching rate increases with the deleterious mutation rate, but drops sharply beyond a critical threshold for very high switching rates (see Supplementary Fig. 9).

### Adaptation rate for different fitness landscape motifs

In the previous section, we computed the rate of adaptation assuming two environment-specific loci, and the fitness of each of the two single mutants in an environment-specific locus is lower than that of the double mutant and wild-type. However, other fitness landscape motifs are possible. In this section, we use simulations to estimate the rate of adaptation for several other motifs with two or three environment-specific loci.

We consider three main categories of fitness landscape motifs: (1) the “fitness valley”, where one of the intermediate genotypes has lower fitness than both wild-type and the adaptive genotype; (2) the “flat” landscape where all intermediate genotypes have the same fitness as the wild-type; and (3) the “monotonic ascent” where each additional mutation results in an increase in fitness. Within each category, the relative values of the fitness of the intermediate genotypes are possible. The motifs considered in this paper are shown in Fig. 1b, along with their names.

**Fig. 4.**
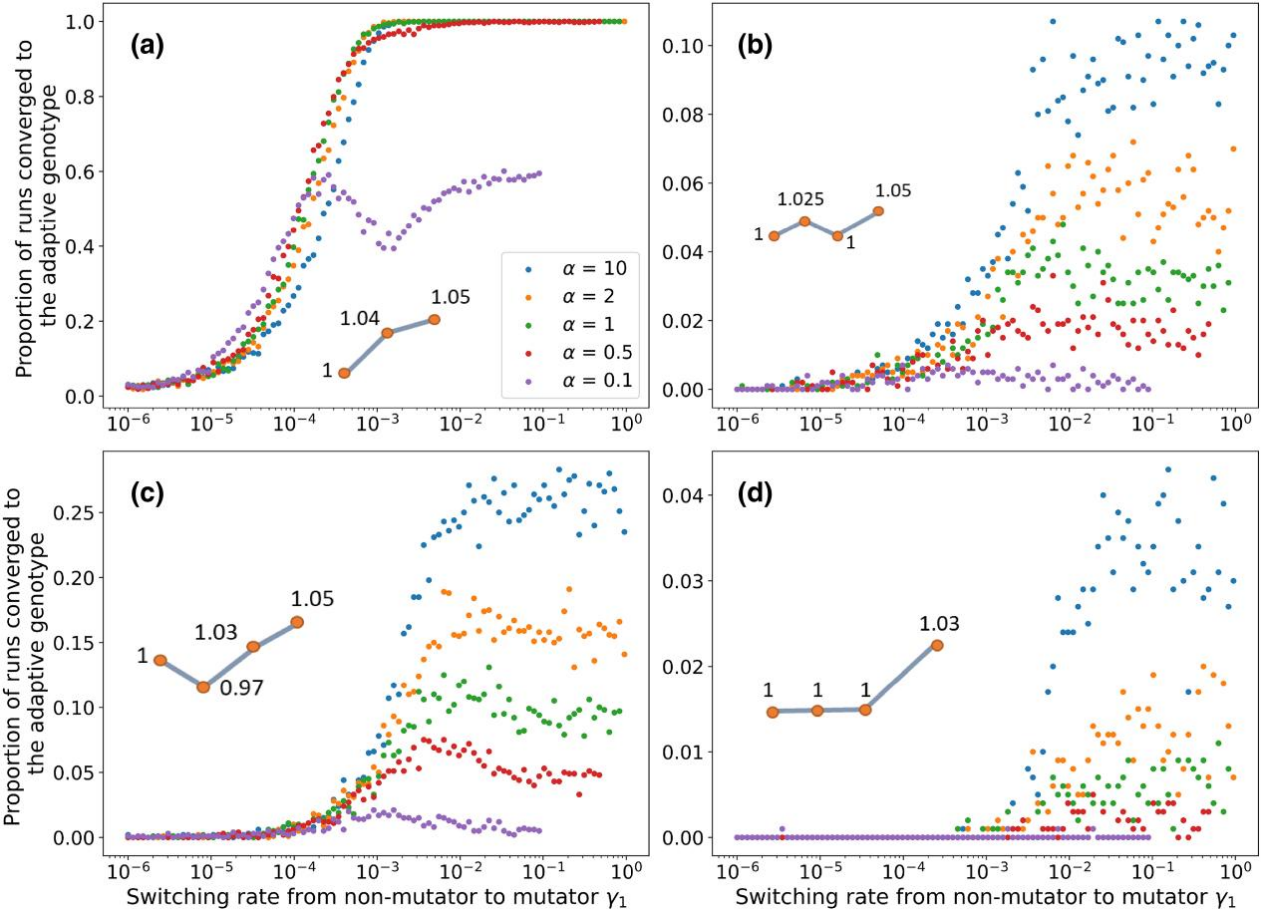
Adaptation rate for several fitness motifs. For each parameter set, 1,000 runs of the stochastic simulation were performed. The proportion of runs that converged to the adaptive genotype were recorded after 1,000 generations. Parameters: $U=4\cdot 10^{-5}$, τ=100, s=0.1, $N=10^{7}$.

For each fitness landscape, we ran 1,000 simulations of the stochastic model for 2,000 generations. The frequencies of each pheno-genotype at generation 0 are the frequencies corresponding to the mutation-selection balance frequencies. In Fig. 4, we plot the proportion of simulations in which the adaptive genotype has reached >99% in frequency after a given number of generations. We use this quantity as a proxy for the rate of adaptation. We also plot additional results for additional fitness motifs in Supplementary Fig. 10.

The rate of adaptation along the switching rate $\gamma_{1}$ is correlated with the predicted probability of appearance from MSB, even when considering three environment-specific loci. However, we do observe that the rate of adaptation does not decrease for high values of $\gamma_{1}$ for the “monotonic ascent” fitness landscape motif plotted in Fig. 4a. For landscapes featuring an increase in fitness with each subsequent mutation, high rates of adaptation were also observed when both $\gamma_{1}<\tau U/2$ and $\gamma_{2}<\tau U/2$. This cannot be explained by a high frequency of mutator mutants (see Fig. 3). Rather, the high adaptation rate must have been due to the dynamics of the mutator frequency during evolution from the wild-type to the adaptive double or triple mutant.

We hypothesized the following mechanism: the mutator, being more likely to generate the genotypes that are intermediate between the wild-type and the adaptive genotype, could hitchhike with one of the intermediate genotypes (either single mutants when considering two environment-specific loci, or double mutants when considering three environment-specific loci) that have higher fitness compared to the wild-type and increase in frequency. This increase in the frequency of intermediate genotypes with a mutator phenotype facilitates the appearance of genotypes with additional mutations, leading to a high rate of adaptation even when both $\gamma_{1}<\tau U/2$ and $\gamma_{2}<\tau U/2$.

**Fig. 5.**
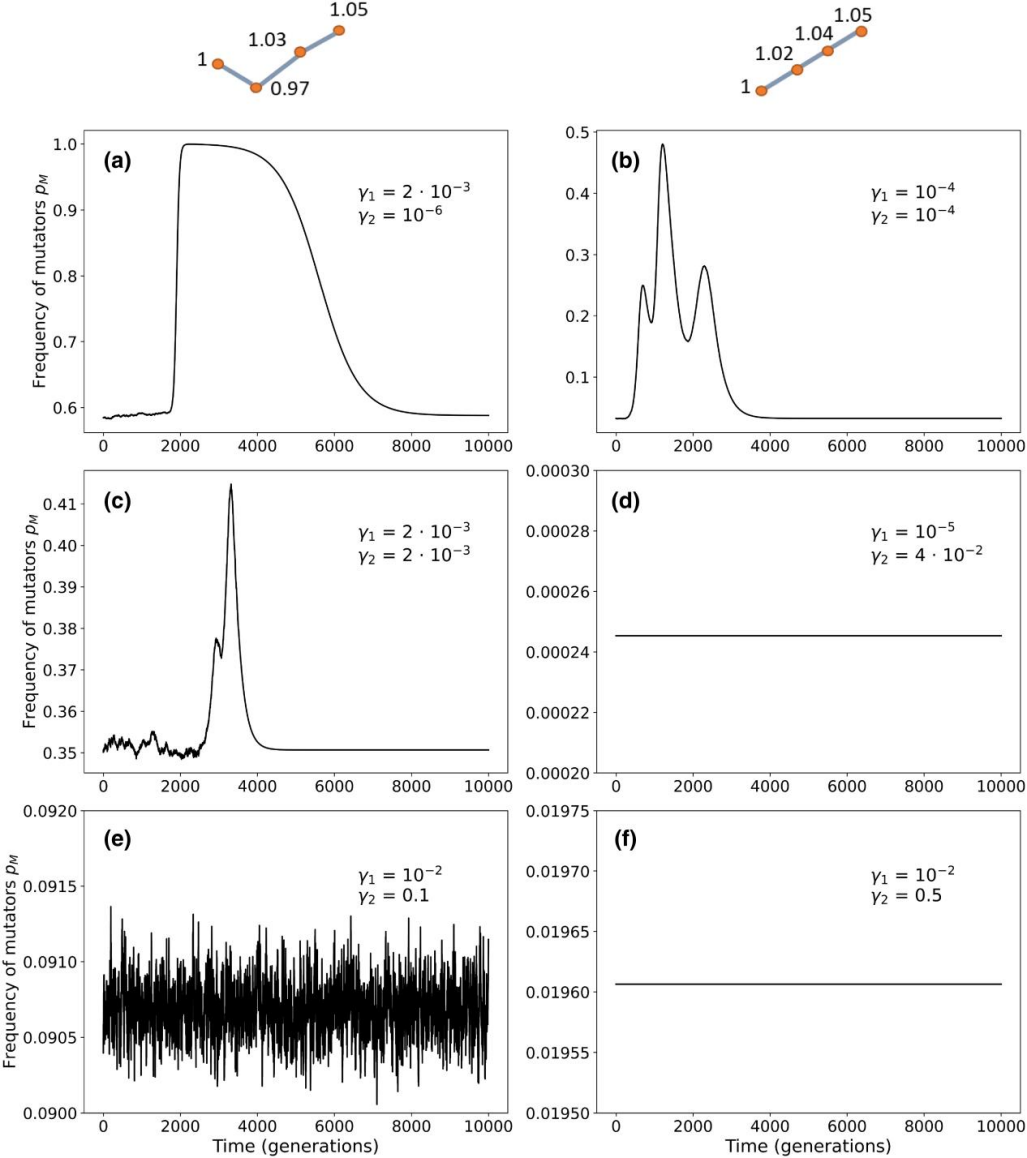
Dynamics of mutator frequency during an adaptive evolution. The frequency of mutators was recorded over 10,000 simulations during adaptation (that is, the appearance and fixation of a fitter genotype). When either of the switching rates is higher than $10^{-3}$, the mutator frequency barely changes from its mutation-selection balance value. However, when switching rates are lower, the mutator hitchhikes with the adaptive genotype, leading to a transient increase in mutator frequency. For a monotone ascent fitness motifs (right column), each appearance and increase in frequency of an adaptive mutation results in an increase of the mutator frequency, thus facilitating the appearance of the next mutation. We suggest that this phenomenon is responsible for the high adaptation rates observed for $\gamma_{1}<\tau U/2$ and $\gamma_{2}<\tau U/2$. Parameters: $U=4\cdot 10^{-5}$, τ=100, s=0.03, n=5000, $N=\;10^{9}$. Values of α in each panel: a) α=2000; b) α=1; c) α=1; d) $\alpha =2.5\cdot 10^{-4}$; e) α=0.1; and (F) α=0.02.

To test this hypothesis, we measured the mutator frequency during adaptive evolution (Fig. 5). In the “monotonic ascent” fitness landscape motif, the three mutations tend to occur and fix very closely together in time, and each fixation leads to an increase in mutator frequency (see bottom line, panel B with $\gamma_{1}=\gamma_{2}=10^{-4}$). The mutator frequency does not return to its mutation-selection balance frequency before the appearance of another adaptive mutant, hence the mutator frequency remains high, facilitating the generation of additional mutations. For $\gamma_{1},\gamma_{2}>10^{-3}$, we observed that the frequency of mutators $p_{M}$ was barely disturbed, if at all, by the appearance of beneficial mutations. In Fig. 5, we can also appreciate that the higher the switching rates, the faster the population returns to mutation-selection balance after the increase in mutator frequency that occurs due to hitchhiking. When either $\gamma_{1}>10^{-2}$ or $\gamma_{2}>10^{-2}$, the mutator frequency at mutation-selection balance is undisturbed. However, when $\gamma_{2}=10^{-6}$, the mutator frequency returns to mutation-selection balance after more than 6,000 generations (defined as being within less than $10^{-3}$ of the mutation-selection frequency). The rate of adaptation decreases with the ratio α between the switching rate from non-mutator to mutator $\gamma_{1}$ to the switching rate from mutator to non-mutator $\gamma_{2}$.

If the speed of return of the mutator frequency to the mutation-selection balance is low, the increased frequency of the mutator phenotype associated with genotypes that are intermediate between the wild-type and the adaptive mutant increases the rate of adaptation.

In summary, stochastic simulations of adaptive evolution have confirmed the analytic results based on mutation-selection balance: the highest adaptation rates are observed for $\frac{\tau U}{2}<\gamma_{1}<10^{-2}$.

Moreover, our stochastic simulations have demonstrated an additional mechanism through which non-genetic systems of mutation-rate inheritance can lead to higher rates of adaptation: hitchhiking of the mutator phenotype with the beneficial mutations it generates causes a transient increase in the population-wide mutation rate, facilitating the accumulation of additional beneficial mutations. This phenomenon is observed when the switching rate from mutator to non-mutator is low enough $\left(\gamma_{2}<\tau U/2\right)$ and leads to higher rates of adaptation than would be expected from the mutation-selection balance analysis, see Fig. 3.

We have thus studied which combinations of the switching rates from the non-mutator to mutator and vice-versa result in high rates of adaptation. We have identified two mechanisms. The first is a “association between mutators and mutants” mechanism: some combinations of the parameter space lead to high frequencies of mutator mutants at mutation-selection balance. This situation occurs due to a high frequency of mutators, while maintaining a non-negligible degree of mutator phenotype inheritance that conserves the association between the mutator phenotype and the mutations it generated. We identified this mechanism through the study of mutation–selection balance frequencies of non-mutator and mutator mutants. The second is a “hitchhiking” mechanism. The hitchhiking of the mutator with the intermediate adaptive mutations facilitates the acquisition of further adaptive mutations. We observed this mechanism through the iteration of our model over what we define as fitness landscape motifs, some of which featured ascending fitness with each subsequent mutation.

## Adaptation on realistic landscapes

In this section, we will determine the relevance of the “association between mutators and mutants” and “hitchhiking” mechanisms to adaptation on biologically realistic fitness landscapes.

We explore several fitness landscapes: NK landscapes of various ruggedness, which can be used to model adaptive evolution (Kauffman 1969), and an empirical landscape derived from the fungus *A. niger* (de Visser *et al*. 2009; de Visser and Krug 2014). A description of these landscapes and the choice of the wild-type genotype can be found in the Supplementary section d (see also Supplementary Fig. 11).

We first estimated the mutation-selection balance frequencies of the non-mutator with the initial genotype ($m_{0}$), the non-mutator with mutations ($m_{1}$), the mutator with the initial genotype ($M_{0}$), and the mutator with mutations ($M_{1}$) for the NK landscapes and for the *A. niger* landscape (Supplementary Fig. 12).

In this section, $m_{0}$ is the frequency of non-mutators with the predominant genotype (the “wild-type”), $m_{1}$ is the frequency of non-mutators with genotypes other than the predominant genotype (“mutants” of the “wild-type”), $M_{0}$ is the frequency of mutators with the predominant genotype (the “wild-type”) and $M_{1}$ is the frequency of non-mutators with genotypes other than the predominant genotype (“mutants” of the “wild-type”). We have $p_{S}=M_{1}+m_{1}$ and $p_{M}=M_{0}+M_{1}$.

We observe similar patterns to those observed with the simple landscapes (see Supplementary Fig. 2): the frequency of $M_{1}$ is highest for $\tau U/2<\gamma_{1}<10^{-2}$. We also estimate the frequency of $p_{M}$ for several parameters sets and the association between mutator and mutant phenotypes in Supplementary Fig. 13. As for the simple landscape, the frequency of $p_{M}$ increases monotonously with the switching rate from non-mutator to mutator $\gamma_{1}$. The association increases to an optimum, before dropping sharply for $\gamma_{1}>10^{-2}$.

**Fig. 6.**
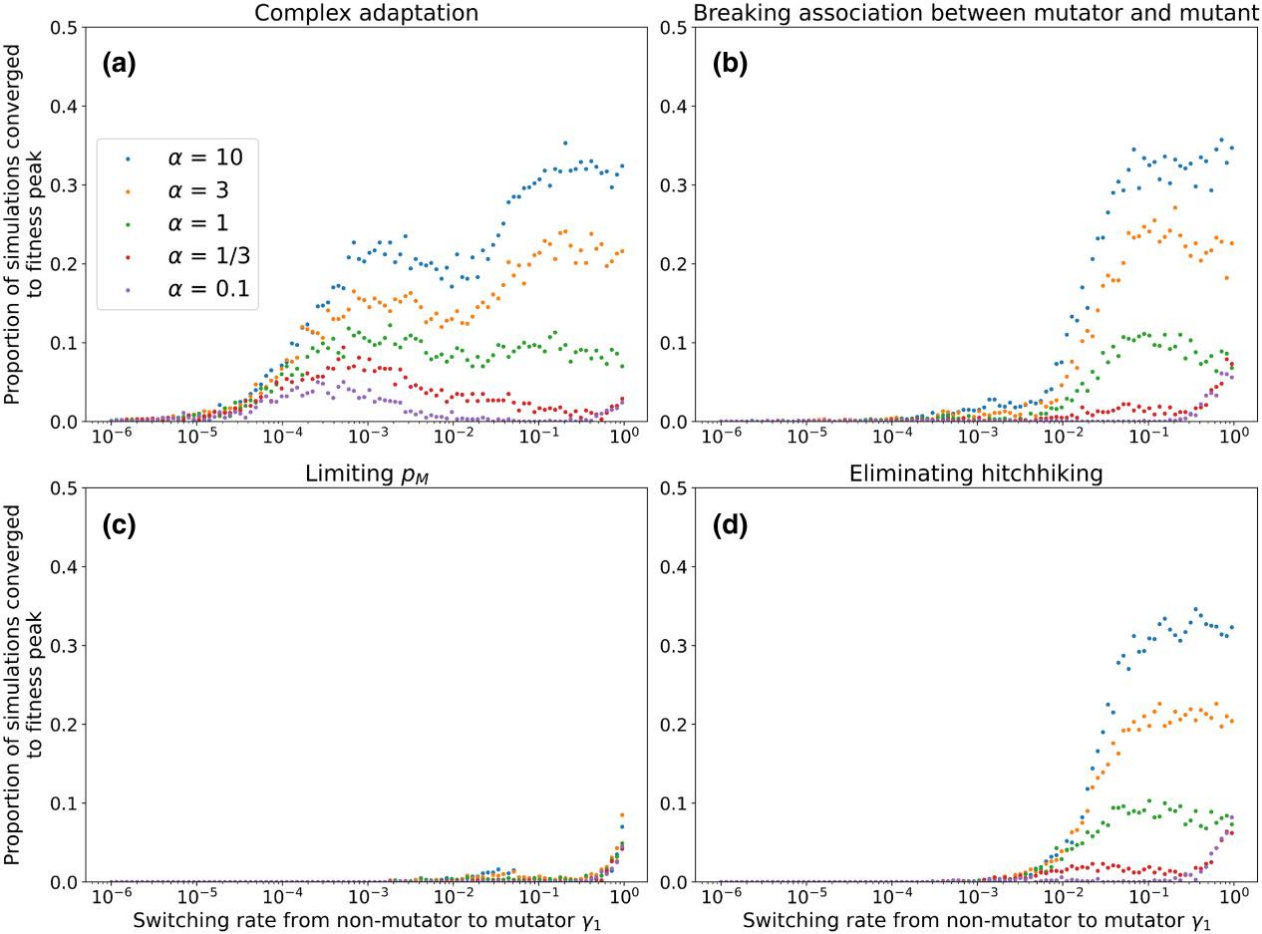
Complex adaptation on *Aspergillus Niger* landscape. The proportion of runs out of 1,000 that converged upon the fittest genotype in the landscape was recorded after 500 generations. In order to disentangle the different effects of the non-genetic inheritance of the mutation rate on the rate of adaptation, we then rerun the simulation while removing the association of mutator and mutant, limiting the frequency of mutators during the evolution, and eliminating hitchhiking. Limiting the frequency $p_{M}$ reduces adaptation overall. Parameters: $U=4\cdot 10^{-5}$, τ=100, N=1000.

We then performed stochastic simulations, initializing the population at MSB frequencies. We observed similar patterns to the stochastic simulations for the simple landscape (see Fig. 6): high rates of adaptation for $\gamma_{1}>\tau U/2$, with sometimes a decrease in the rate of adaptation when $\gamma_{1}$ become large. These observations indicated to us that the “association between mutators and mutants” mechanism, that is, the combination of a high frequency of mutators with a high association between the mutator and mutant phenotypes, could be at work also in the case of complex adaptation.

We found that the highest rates of adaptation correspond to the region expected from the frequencies of mutator mutants at mutation-selection balance (Supplementary Fig. 12). We also found an additional region with high adaptation rate for $\gamma_{1}<\tau U$ and $\gamma_{2}<\tau U$ for NK landscapes with k=1 (that is, low ruggedness, see Supplementary section d, Description of realistic landscapes) and k=3 (that is, intermediate ruggedness, see Supplementary section d), analogous to the region observed for these parameters for populations adapting over smooth landscapes in Fig. 4. This is a manifestation of the “association between mutators and mutants” mechanism. We observe that $\gamma_{1}<\tau U$ and $\gamma_{2}<\tau U/2$ correspond to high rates of adaptation for NK landscapes with low ruggedness, but not in NK landscapes with high ruggedness. The “hitchhiking” mechanism explains why the rate of adaptation is higher for these combinations of $\gamma_{1}$ and $\gamma_{2}$ on smoother landscapes. Hence, we find examples of both of the “association between mutators and mutants” and “hitchhiking” mechanisms at play in complex adaptation over biologically realistic landscapes.

Next, we determined the relative contribution of the “association between mutators and mutants” and “hitchhiking” mechanisms to the rate of adaptation. We simulated the stochastic model while artificially modifying the frequency of the mutator phenotype to eliminate the influence of one of the three conditions that lead to a high adaptation rate: (1) high association between the mutator and mutant frequency—we set the association (see Eq. 6) to equal 0 by redistributing mutant genotypes among mutator and non-mutators; (2) for the hitchhiking mechanism of the mutator phenotype with an adaptive mutant—we set the mutator frequency to always be the mutator frequency at mutation-selection balance; and (3) a high mutator frequency—we set the mutator frequency to be half the mutator frequency at mutation-selection balance.

### Breaking the association between the mutator and the mutant

High association between mutator and mutant translates into a high frequency of mutator mutants, and in turn increases the rate of adaptation. Hence, we decided to artificially decrease the association between the mutator and the mutant, while keeping the frequency of mutator and the frequency of mutants, thus eliminating this effect from the evolutionary dynamics. At each generation, we artificially break the association (A=0) by setting $m_{0}=(1-p_{S})\cdot (1-p_{M})$, $m_{1}=p_{S}\cdot (1-p_{M})$, $M_{0}=(1-p_{S})\cdot p_{M}$, and $M_{1}=p_{S}\cdot p_{M}$ (see Supplementary Section h). The relative frequencies of each mutant within $m_{1}$ and $M_{1}$ is not modified. Note that when association between the mutator and mutant is broken, hitchhiking is also abolished since the mutator cannot hitchhike on a mutant to increase in frequency.

We observe that the rate of adaptation is mostly unaffected except for the regions of the parameter space that correspond to hitchhiking, that is $\gamma_{1}<\tau U$ and $\gamma_{2}<\tau U/2$. Hence, the association between mutator and mutant has less influence for complex adaptation (see Fig. 6 and Supplementary Figs. 14–17).

### Eliminating hitchhiking

To eliminate hitchhiking, we reset the proportion of mutators $p_{M}$ at each generation to correspond to the value of $p_{M}$ at mutation-selection balance. Note that in this case, the association between mutator and mutant is also at its mutation-selection balance, that is, it can be different than 0. The relative frequencies of wild-type and mutant genotypes were not modified (see Supplementary Section i).

As described in the previous section, the hitchhiking mechanism only manifests for both $\gamma_{1}<\tau U/2$ and $\;\gamma_{2}<\tau$. Indeed, we observed a significantly reduced rate of adaptation for the parameter region $\gamma_{1}<\tau U$ and $\gamma_{2}<\tau U\;$ (Fig. 6 and Supplementary Fig. 18). Without hitchhiking, only the parameter region exhibiting high frequencies of mutator mutants at mutation-selection balance showed high rates of adaptation, consistent with the expectation that only the “association between mutators and mutants” mechanism affects adaptation. On NK landscapes, eliminating hitchhiking reduces the adaptation rate for $\gamma_{1}<\tau U$ and $\gamma_{2}<\tau U$ for low and intermediate ruggedness, but we observe no effect for high ruggedness, further supporting our hypothesis that hitchhiking only occurs on smooth landscapes where successive adaptations occur without the population returning to a mutation-selection balance (Supplementary Figs. 14–17).

### Reducing the mutator frequency

In the previous section, we observed that high adaptation rates are associated with high mutator frequency, which in turn is associated with high mutator mutant frequency. To reduce the mutator frequency, we reset the proportion of mutators $p_{M}$ to half of its mutation-selection balance value at each generation. The relative frequencies of wild-type and mutant genotypes were not modified (see Supplementary Section j). Note that in this simulation, hitchhiking is also abolished since the mutator cannot hitchhike on a mutant to increase in frequency. The rate of adaptation is reduced, mostly for the areas where $p_{M}>0.5$ at mutation-selection balance (Fig. 6 and Supplementary Fig. 18). However, we still observe relatively high adaptation rates with $\gamma_{1}=\tau U$ when $\gamma_{2}<\tau U$ and $\gamma_{1}=\gamma_{2}$ when $\gamma_{2}>\tau U$. Results of simulation on NK landscapes are similar (Supplementary Figs. 14–17).

In summary, we studied how the “association between mutators and mutants” and “hitchhiking” mechanisms affect the evolution over realistic landscapes. The “association between mutators and mutants” mechanism refers to the advantage in adaptation stemming from the frequency of coupled mutator mutants at mutation-selection balance. The frequency of mutator mutants is high when the frequency of mutators is high. The association between mutator and mutants seems to play a smaller role on complex landscapes than on the simple landscape. The “hitchhiking” mechanism manifests on landscapes with low ruggedness and $\gamma_{1},\;\gamma_{2}<\tau U$. When removing the effect of hitchhiking, we observe low adaptation for specifically $\gamma_{1},\;\gamma_{2}<\tau U$.

## Discussion

### The potential advantage of non-genetic inheritance of the mutation rate

We have shown that the combination of the switching rates that lead to high adaptation rates on simple landscapes also result in high adaptation rates on complex, more biologically realistic landscapes. The result is maintained also when considering gradual transition between the non-mutator and mutator phenotypes (see Supplementary Fig. 1). The advantageous combination of switching rates corresponding to non-genetic inheritance of the mutation rate in the rate of adaptation seems to come from two mechanisms, a association between mutators and mutants mechanism relying on a high frequency of mutator mutants that exists even at mutation-selection balance, and a hitchhiking mechanism that manifests itself while hill climbing of fitness landscape.

The association between mutators and mutants mechanism leads to high rates of adaptation for $\gamma_{1}=\tau U/2$ when $\gamma_{2}<\tau U/2$ and $\gamma_{1}=\gamma_{2}$ when $\gamma_{2}>\tau U/2$. For highest rates of adaptation to be achieved, the inheritance of the mutation rate phenotype needs to be non-negligible, hence $\gamma_{1}<10^{-2}$ and $\gamma_{2}<10^{-2}$. For smooth landscapes with potential hill climbing, the hitchhiking mechanism also leads to high adaptation rates for $\gamma_{2}<\tau U/2$ and $10^{-2}<\gamma_{1}<\tau U/2$.

After performing simulations attempting to eliminate each of these mechanisms to assess their relative contributions in complex adaptation we deduce that the association between mutators and mutants mechanism seems to be determining, since reducing the proportion of $p_{M}$ reduces the rate of adaptation. The hitchhiking mechanism is only important for a subset of the parameter space, for $\gamma_{1}<\tau U/2$ and $\gamma_{2}<\tau U/2$, and for smooth landscapes.

### Evolution of intermediate switching rates

Although some combinations of switching rates maximize the adaptation rate, it is not clear that they can be selected for when competing against other combinations of switching rates. First, the proportion of mutants for the adaptation-optimal pairs of switching rates is high, higher than for subpopulations with adaptation-suboptimal combinations of switching rates (see Fig. 3). Second, the advantage of a subpopulation with an adaptation-optimal switching rate depends on the mutation supply. The adaptation rate of a subpopulation of size $N_{1}$ with a switching rate $\gamma_{1}$ resulting in an appearance rate $q_{1}$ will be higher than the adaptation rate in a subpopulation of size $N_{2}$ with a given switching rate $\gamma_{2}$ resulting in an appearance rate $q_{2}$ if $\frac{N_{2}}{N_{1}}>\frac{q_{1}}{q_{2}}$ (Eq. 5). Therefore, selection for an allele that induces an intermediate switching rates will depend on its frequency in the population, similar to the case of mutator alleles (Chao and Cox 1983).

### The definition of the mutator phenotype in biology

Mutators are traditionally thought of genetically inherited. Most theoretical and experimental literature focuses on mutators phenotypes resulting from the loss of function of proteins involved in DNA replication and repair. Yet, in our analysis, the switching rates corresponding to genetic inheritance consistently resulted in very low rates of adaptation compared to switching rates corresponding to non-genetic inheritance. This finding reshapes the picture of mutators in biology. Non-genetic mutators are likely to be of higher evolutionary importance than genetic mutators.

### The potential advantage of contrarian inheritance

For specific parameter sets, the switching rates that maximized adaptation were higher than 0.5. Moreover, one of the three systems for non-genetic inheritance of the mutation rate, the Ada protein, has an estimated $\gamma_{2}$ that is higher than 0.5. Hence, for specific parameter sets, it is more advantageous to switch to the phenotype that is opposite to the parental phenotype rather than inherit it. Note that this is different than no inheritance of the parental phenotype. For contrarian inheritance there must be a memory of the parental phenotype. But, instead of being perpetuated, as is usually the case of inheritance, the phenotype is inversed.

### Mutation rate inheritance and phenotype switching

Switching between a mutator and a non-mutator phenotype is a type of phenotype switching (Levien *et al*. 2020). The increased adaptability of populations with adaptation-optimal pairs of the two switching rates, compared to other pairs, is in part due to the high rate of mutator generation, which balances the strength of selection against mutators at mutation-selection balance. Generation of a phenotype that is disadvantageous in the short term but potentially advantageous in the long term is known as bet-hedging (Levien *et al*. 2020) and has been implicated as driving the evolution of phenotype switching mechanisms (Carja *et al*. 2014; Stajic *et al*. 2021; Levien *et al*. 2020; Gómez-Schiavon and Buchler 2019).

We suggest that the mechanisms we identified to be implicated in high rates of adaptation for some pairs of switching rates could be at play also in other systems in which a specific phenotype has a short-term disadvantage but long-term advantage. The mutator state could be one such phenotype. High switching rate from wild-type to phenotype (in our case, the switching from non-mutator to mutator) counters negative selection, and high association between the phenotype allows for the manifestation of the long-term advantage. In such cases, the same phenotype may also be advantageous if it occurs in consecutive generations of the same lineage, and therefore a not too high switching rate (in our model, less than $10^{-2}$) is beneficial in maintaining a correlation between the phenotype of parent and offspring.

### Experimental future directions

Our results show theoretically that non-genetic inheritance of the mutation rate could result in higher rates of adaptation as a general rule. Interestingly, current methods for estimating the mutation rate, such as mutation accumulation (Lynch *et al*. 2008) and fluctuation assays (Lang and Murray 2008), require multiple generations along which mutation rate is measured. Hence, they implicitly assume that the mutation rate is constant across generations and individuals. This is why diversity and non-genetic inheritance of the mutation rate has not been observed until recently (Uphoff *et al*. 2016; Robert *et al*. 2018; Woo *et al*. 2018). Our study provides an evolutionary rationale for these observed mutation rate dynamics and it calls for a new focus on non-genetic factors influencing the mutation rate, and its potential impact on adaptative evolution.

## Supplementary Material

iyad111_Supplementary_Data

iyad111_Supplementary_Data

## Data Availability

The code necessary to reproduce the results and the plots presented in this paper is available at https://github.com/gabriela3001/revision_phenotypic_mrate. Supplemental material available at GENETICS online.

## Appendix to: Phenotype switching of the mutation rate facilitates adaptive evolution

### A. The Wright-Fisher model

The Wright-Fisher model is commonly used to model evolutionary biology. In this model, a population is described by a probability vector that contains the frequency of each genotype that appears in the population. This population vector is modified by iterating mutation-selection-drift steps. In the mutation step, the population vector is multiplied by a transition matrix that gives the probability of mutation between each pair of genotypes. In the selection step, the frequency of each genotype is multiplied by its relative fitness, and then the population vector is normalized so that it sums to one by dividing with the population mean fitness. In the drift step, stochasticity is introduced by randomly sampling the population vector of the next generation from a multinomial distribution characterize by the population vector of the current generation after the mutation and selection steps. One mutation-selection-drift cycle corresponds to a single generation of the population (Otto and Day 2019).

### B. Discussion about fitness landscapes

Biological fitness landscapes are notoriously difficult to investigate due to their high dimensionality. Indeed, a DNA sequence of length *N* corresponds to $4^{N}$ genotypes, and a protein sequence of length *N* corresponds to $20^{N}$ possible proteins. Considering the average length of a gene, or that of a protein, it quickly becomes obvious that all possible sequences cannot be possibly surveyed empirically. Existing experimental studies of fitness landscapes have focused either on local landscapes around proteins (Sarkisyan et al. 2016; Li et al. 2016) or short DNA sequences (Aguilar-Rodríguez, Payne, and Wagner 2017). Traditionally, fitness landscapes were described as containing many “valleys”, corresponding to low fitness genotypes, and “peaks”, corresponding to high fitness genotypes. Therefore, a central problem in evolutionary biology was to explain how fitness valleys can be crossed when natural selection acts to eliminate low-fitness intermediate genotypes (Wright 1931). A few examples of fitness valley crossing have been suggested (Serrano et al. 1997; Levin, Perrot, and Walker 2000), along with strategies for their crossing such as capacitance (Trotter et al. 2014; Griswold and Masel 2009), partial robustness (Kim 2007), stress-induced mutagenesis (Ram and Hadany 2014), or phenotypic variation (Whitehead et al. 2008). Yet, due to the hyper-dimensionality of biological fitness landscapes, fitness valleys could actually rarely exist, and fitness landscapes may thus be more highly navigable than often appreciated (Forrest 2022). Hence, evolutionary adaptation over rugged landscapes would be more akin to a diffusion problem in which adaptation is dependent on the time needed to find a mutational path that does not contain a fitness valley (van Nimwegen and Crutchfield 2000). The empirical evidence for either an abundance or lack of fitness valleys is scarce, as observing fitness valley crossings poses technical difficulties due to the very low frequency of the low fitness intermediate genotype.

### C. Simple landscape

The mutation transition probability is $u_{\;j\to g}$, where *j* and *g* are genotypes, determines the effect of mutation on the genotype probabilities (Eq. 1). The construction of the mutation transition probability is described in the main text. Here, we provide a formal description. Note that we assume no back mutations occur. In the following we use the mutator phenotype mutation rate τU; for the non-mutator, we set τ=1. The per-locus mutation rate is μ=U/n.

The number of pre-existing background mutations in the source genotype is denoted by *k*. The number of background mutations acquired during the mutation step is denoted by *l*. Hence, the probability of transition from genotype 0∖1 to genotype 0∖3 corresponds to k=1 and l=2.

Thus, the probability to transition from genotype *j* to genotype *g* for the mutator genotype, $u_{\;j\to g}^{\prime }$, is described by Table A1, where the source genotype *j* is given in the row, and the target genotype *g* is given in the column.

**Table A1.** Probabilities to transition from genotype *j* to genotype *g*, $u_{j\to g}^{\prime }$.

$u_{\;j\to g}^{\prime }$	g=0∖(k+l)	g=1∖(k+l)	g=2∖(k+l)
j=0∖k	$(1-\tau \mu {)}^{2}\cdot \frac{e^{\tau U}{\left(\tau U\right)}^{l}}{l!}$	$2\tau \mu (1-\tau \mu )\cdot \frac{e^{\tau U}{\left(\tau U\right)}^{l}}{l!}$	$(\tau \mu {)}^{2}\cdot \frac{e^{\tau U}{\left(\tau U\right)}^{l}}{l!}$
j=1∖k	0	$(1-\tau \mu )\cdot \frac{e^{\tau U}{\left(\tau U\right)}^{l}}{l!}$	$\tau \mu \cdot \frac{e^{\tau U}{\left(\tau U\right)}^{l}}{l!}$
j=2∖k	0	0	$\frac{e^{\tau U}{\left(\tau U\right)}^{l}}{l!}$

### D. Description of complex landscapes

#### NK landscapes

The NK landscape is commonly used in the study of epistatic interactions (Obolski, Ram, and Hadany 2018). It has two parameters: *n* for the number of bi-allelic loci in the genotype and *k* for the number of loci each locus interacts with. The main advantage of the NK landscape is its biological interpretation and its tunable ruggedness (S. Kauffman and Levin 1987). Here, the genotype consists of n=6 bi-allelic loci, which results in $2^{6}=64$ genotypes.

We chose n=6 in order to allow for multidimensionality, while maintaining reasonable computation times.

To construct an NK landscape, we first generate all possible *k*-bit strings (bit strings of length *k*) and assign to each of them a random fitness value between 0 and 1, sampled from a continuous uniform distribution. Genotype *g* is a *n*-bit string, and its fitness *w_g_* is the sum of the fitness effects of the *k-*bit strings that *g* contains. A locus thus influences the genotype fitness according to the number of *k-*bit strings that contain it, which increases with *k*. Hence, the ruggedness of the NK landscape increases with *k* (De Visser, Park, and Krug 2009). We consider three NK landscapes with low, intermediate, and high ruggedness corresponding respectively to *k* = 1, *k* = 3, and *k* = 5. See Figure S11 for properties of the constructed landscapes.

#### Empirical fitness landscape from *Aspergillus niger*

We examine the insights gained on two-peak and NK landscapes with an empirical fitness landscape (Obolski, Ram, and Hadany 2018) measured with mutants in 8 genes of *Aspergillus niger* (De Visser, Park, and Krug 2009). We chose this landscape because: (i) it is complete with fitness measurements of all possible 256 genotype combinations, so we can avoid the interpolation of fitness values of missing genotypes; and (ii) fitness was measured as growth rate relative to the wild type, as opposed to other studies that quantify some proxy phenotype for fitness such as fluorescence or DNA binding (Aguilar-Rodríguez, Payne, and Wagner 2017; Aguilar-Rodríguez et al. 2018; Banerjee et al. 2017).

The genotype consists of eight bi-allelic loci, each with either the wild type or the mutant allele. de Visser *et al.* (De Visser, Park, and Krug 2009) engineered 256 genotypes to bear all possible combinations of wild type/mutant alleles in these eight loci. The loci are: *fwnA1* (fawn-colored conidiospores); five auxotrophic markers, *argH12* (arginine deficiency), *pyrA5* (pyrimidine deficiency), *leuA1* (leucine deficiency), *pheA1* (phenyl-alanine deficiency), and *lysD25* (lysine deficiency); and two resistances, *oliC2* (oligomycin resistance) and *crnB12* (chlorate resistance).

de Visser *et al.* (De Visser, Park, and Krug 2009) estimated fitness by measuring the growth rate of each strain relative to the growth rate of the wild type strain (i.e., with eight wild type alleles). Out of 256 genotypes, 70 genotypes were lethal (fitness is zero). The landscape is rugged: it contains 15 local maxima (including the global maximum). See Figure S11 for properties of the landscape.

### E. Calculating the switching rate from mutator to non-mutator $\gamma_{2}$ for the Ada protein

We define a Markov chain with two states: cells with zero Ada molecule denoted by *x* and cells with non-zero Ada molecule denoted by *y*.

The transition matrix *T* is given by

$$T=\left(\begin{array}{cc} \;p_{x\to x} & \;p_{x\to y}\\ \;p_{y\to x} & \;p_{y\to y} \end{array}\right)$$

where $p_{x\to x}=1-p_{x\to y}$ and $p_{y\to y}=1-p_{y\to x}$. In (Uphoff et al. 2016), the probability of transitioning from zero to one (or more) Ada molecules is fitted to a Poisson distribution with average equal to 1. Hence, we have $p_{x\to x}=0.37$ and $p_{x\to y}=1-p_{x\to x}=0.63$.

The stationary distribution of this Markov chain is denoted by $\left(x^{*},\;y^{*}\right)$ with $y^{*}=1-x^{*}$. Empirical evidence (Uphoff et al. 2016) suggests that $\left(x^{*},y^{*})\approx (0.25,0.75\right)$. To estimate $p_{y\to y}$, we solve

$$x^{*}=p_{x\to x}\;x^{*}+p_{y\to x\;}y^{*}$$

$$y^{*}=p_{x\to y}\;x^{*}+p_{y\to y\;}y^{*}$$

to obtain

$$p_{y\to y}=1-\frac{\;p_{x\to y}\;x^{*}}{y^{*}}$$

Hence, the complete transition matrix is

$$T=\left(\begin{array}{cc} 0.37 & 0.63\\ 0.21 & 0.79 \end{array}\right)$$

### F. Bounds on the population size

We consider *N*, the population size, to be large enough so that individuals with a single mutation in the major loci are present, but small enough so that individuals with two mutations in the major loci (hence already adapted to the new environment) are absent.

Let us first consider the first condition. The lowest frequency of mutants is achieved when the whole population is non-mutator. Therefore, the upper bound for the first condition is set by considering a case where all individuals have the non-mutator phenotype. According to (Gordo and Dionisio 2005), the frequency of wild type individuals at mutation-selection balance is $e^{-U/s}$. We then multiply by the mutation-selection balance of single mutants at the major loci, $\frac{\mu }{s}$. Thus, we find that the expected number of single mutant individuals without deleterious mutations in the background loci is $N\frac{\mu }{s}e^{-U/s}$. Setting this to be less than one and rearranging, we obtain $\frac{s}{\mu }e^{U/s}<N$.

Now let us consider the second condition. The highest frequency of double mutants would be achieved when the whole population is mutator. Therefore, the lower bound for the second condition is set by considering a case where all individuals have the mutator phenotype. By the same argument as for the first condition, we find $N<{\left(\frac{s}{\tau \mu }\right)}^{2}e^{\frac{\tau U}{s}}$ with $e^{\frac{-\tau U}{s}}$ the proportion of wildtype individuals in an all-mutator population and ${\left(\frac{\tau \mu }{s}\right)}^{2}$ the frequency of double mutants in the major loci, both at a mutation selection balance.

Combining these conditions, we have

(A1)
$$\frac{s}{\mu }e^{U/s}<N<{\left(\frac{s}{\tau \mu }\right)}^{2}e^{\frac{\tau U}{s}}$$

### G. Fixation of an adaptive genotype

We set the fitness of the double mutant to be 1+sH, where *H* is the adaptation coefficient. According to Eshel (Eshel 1981), in a large population with weak selection, the fixation probability $\rho_{F}\;$of a double mutant once it appears in a single copy depends only on the selection coefficient *s* and the double mutant advantage *H*, namely

(A2a)
$${\tilde{\rho }}_{F}=\frac{2sH}{1+sH}$$

(A2b)
≈2sH,

where Eq. A2b applies when *sH* is much smaller than 1.

Figure S6 shows a comparison of the two approximations in eq. A2 to results from stochastic simulations obtained by counting the number of fixation events after the appearance of an adaptive genotype.

We notice that Eq. A2b is independent from the switching rate γ. Hence, the adaptation rate will be proportional to the appearance rate (see Figure S3). We therefore consider the appearance rate as a proxy for the adaptation rate in the main text.

### H. Complex landscapes: breaking the association between mutators and mutants

The stochastic model was run following the usual mutation—phenotypic switching—selection—drift scheme. Right before the drift step, the population vector was recalculated as follows:

$$M_{1}^{\prime }=\max(\;p_{S}\cdot p_{M},0)$$

$$m_{1}^{\prime }=\max(\;p_{S}\cdot (1-p_{M}),0)$$

$$M_{0}^{\prime }=\max((1-p_{S})\cdot p_{M},0)$$

$$m_{0}^{\prime }=\max(1-M_{1}^{\prime }-m_{1}^{\prime }-M_{0}^{\prime },0)$$

The genotype 0 is the most abundant genotype at that generation. All the mutants’ (mutants are all genotypes that are not the genotype 0) frequencies are kept identical relative to each other, but their sum is set to $M_{1}^{\prime }$ (for mutator mutants) or $m_{1}^{\prime }$ (for non-mutator mutants).

### I. Complex landscapes: eliminating hitchhiking

The stochastic model was run following the usual mutation—phenotypic switching—selection—drift scheme. Right before the drift step, the population vector was recalculated as follows:

$$M_{1}^{\prime }=\max(A+p_{M}\cdot p_{S},0)$$

$$m_{1}^{\prime }=\max(\;p_{S}-M_{1}^{\prime },0)$$

$$M_{0}^{\prime }=\max(\;p_{M}-M_{1}^{\prime },0)$$

$$m_{0}^{\prime }=\max(1-M_{1}^{\prime }-m_{1}^{\prime }-M_{0}^{\prime },0)$$

The genotype 0 is the most abundant genotype at that generation. All the mutants’ (mutants are all genotypes that are not the genotype 0) frequencies are kept identical relative to each other, but their sum is set to $M_{1}^{\prime }$ (for mutator mutants) or $m_{1}^{\prime }$ (for non-mutator mutants).

### J. Complex landscapes: limiting $p_{M}$

The stochastic model was run following the usual mutation—phenotypic switching—selection—drift scheme. The desired frequency of mutators is equal to *M*. Right before the drift step, the population vector was recalculated as follows:

$$M_{1}^{\prime }=\max(A+p_{M}\cdot p_{S},0)$$

$$m_{1}^{\prime }=\max(\;p_{S}-M_{1}^{\prime },0)$$

$$M_{0}^{\prime }=\max(M-M_{1}^{\prime },0)$$

$$m_{0}^{\prime }=\max(1-M_{1}^{\prime }-m_{1}^{\prime }-M_{0}^{\prime },0)$$

The genotype 0 is the most abundant genotype at that generation. All the mutants’ (mutants are all genotypes that are not the genotype 0) frequencies are kept identical relative to each other, but their sum is set to $M_{1}^{\prime }$ (for mutator mutants) or $m_{1}^{\prime }$ (for non-mutator mutants).
