# Supplementary material for: Phenotype switching of the mutation rate facilitates adaptive evolution
Source: Genetics. 2023 Jun 9;225(1):iyad111. doi: 10.1093/genetics/iyad111 (PMC10471227; doi:10.1093/genetics/iyad111)
Supplement: iyad111_Supplementary_Data [file iyad111_supplementary_data.docx]

**Supplementary Figures for: Phenotype switching of the mutation rate facilitates adaptive evolution**

Gabriela Lobinska^1^, Yitzhak Pilpel^1*^, Yoav Ram^2*^

^1^ Department of Molecular Genetics, Weizmann Institute of Science, Rehovot 76100 Israel

^2^ School of Zoology, Faculty of Life Sciences, Tel Aviv University, Tel Aviv 6997801 Israel

* Correspondence: [pilpel@weizmann.ac.il](mailto:pilpel@weizmann.ac.il), [yoav@yoavram.com](mailto:yoav@yoavram.com)


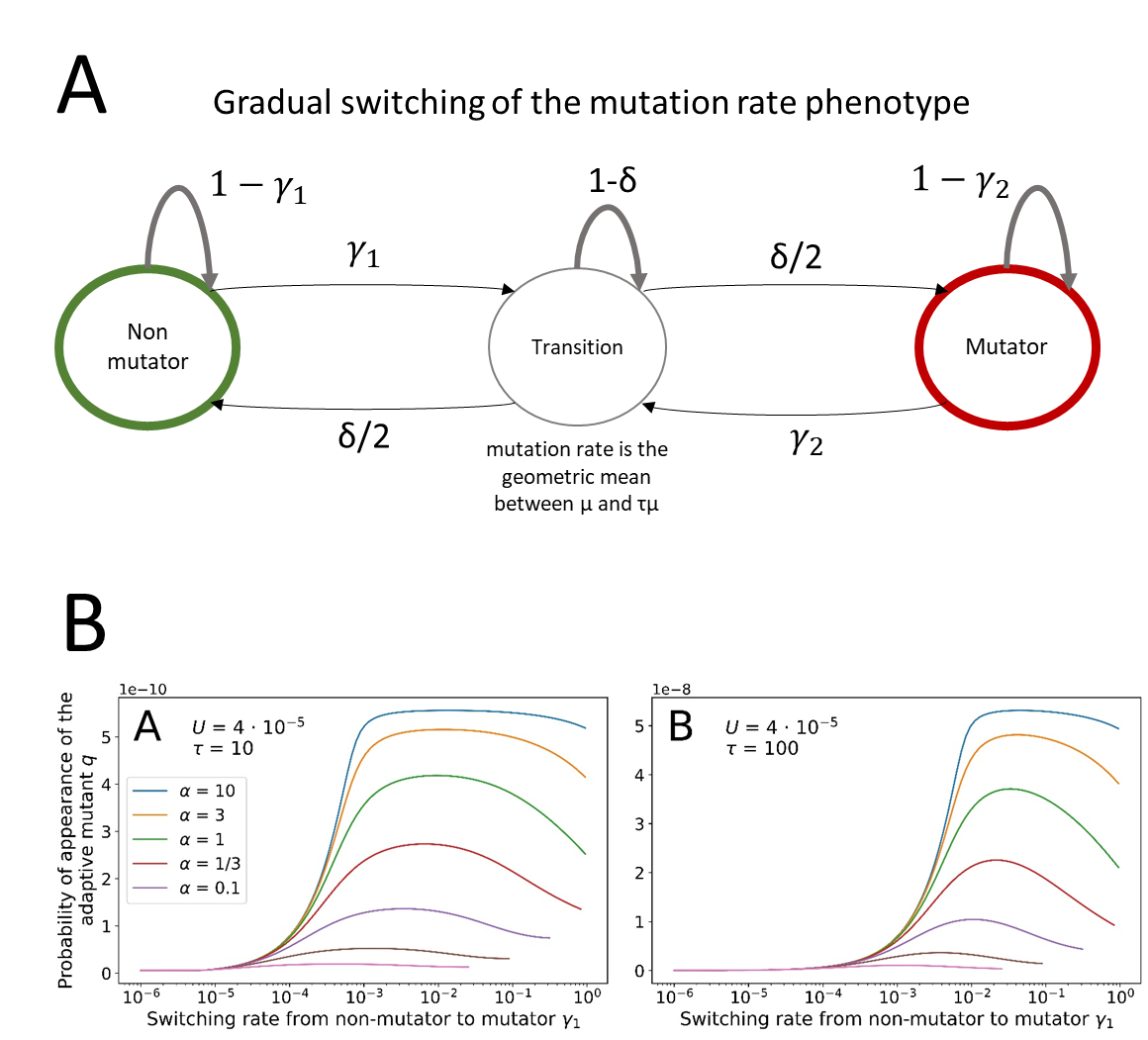


**Figure S1. (A)** **Model extension for gradual switching of the mutation rate phenotype.** In this model extension, we introduce an additional, transitional genotype with mutation rate equal to the geometric mean between the non-mutator and the mutator mutation rate. The switching rate $\gamma_{1}$ indicates the switching from the non-mutator to this transition phenotype; a new parameter, $\delta$/2, governs the switching from the transition to the mutator phenotype. By symmetry, we also have the switching rate $\gamma_{2}$ from the mutator to the transition phenotype and $\delta$/2 the probability of switching from the transition phenotype to the non-mutator phenotype. **(B) Predicted probability of appearance of adaptive genotype for model with gradual transition between the non-mutator to the mutator phenotype and vice-versa.** Parameters: $s=0.1$.


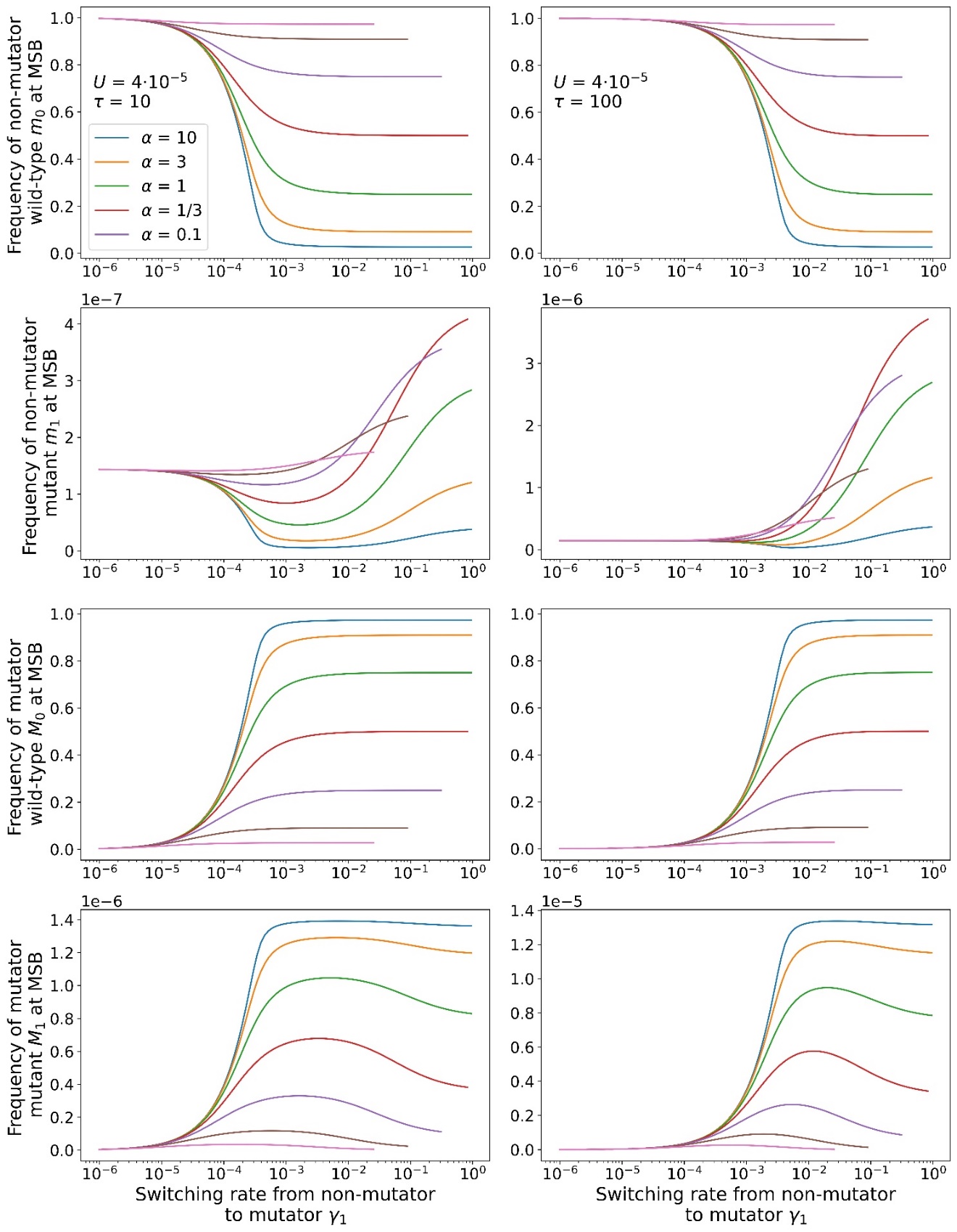


**Figure S2. Frequencies of non-mutator wild-type, non-mutator mutant, mutator wild-type, and mutator mutant at MSB for two values of the mutation rate** $\boldsymbol{U}$ **and two value of the fold-increase in mutator mutation rate** $\boldsymbol{\tau}$**.** Parameters: $s=0.1$.


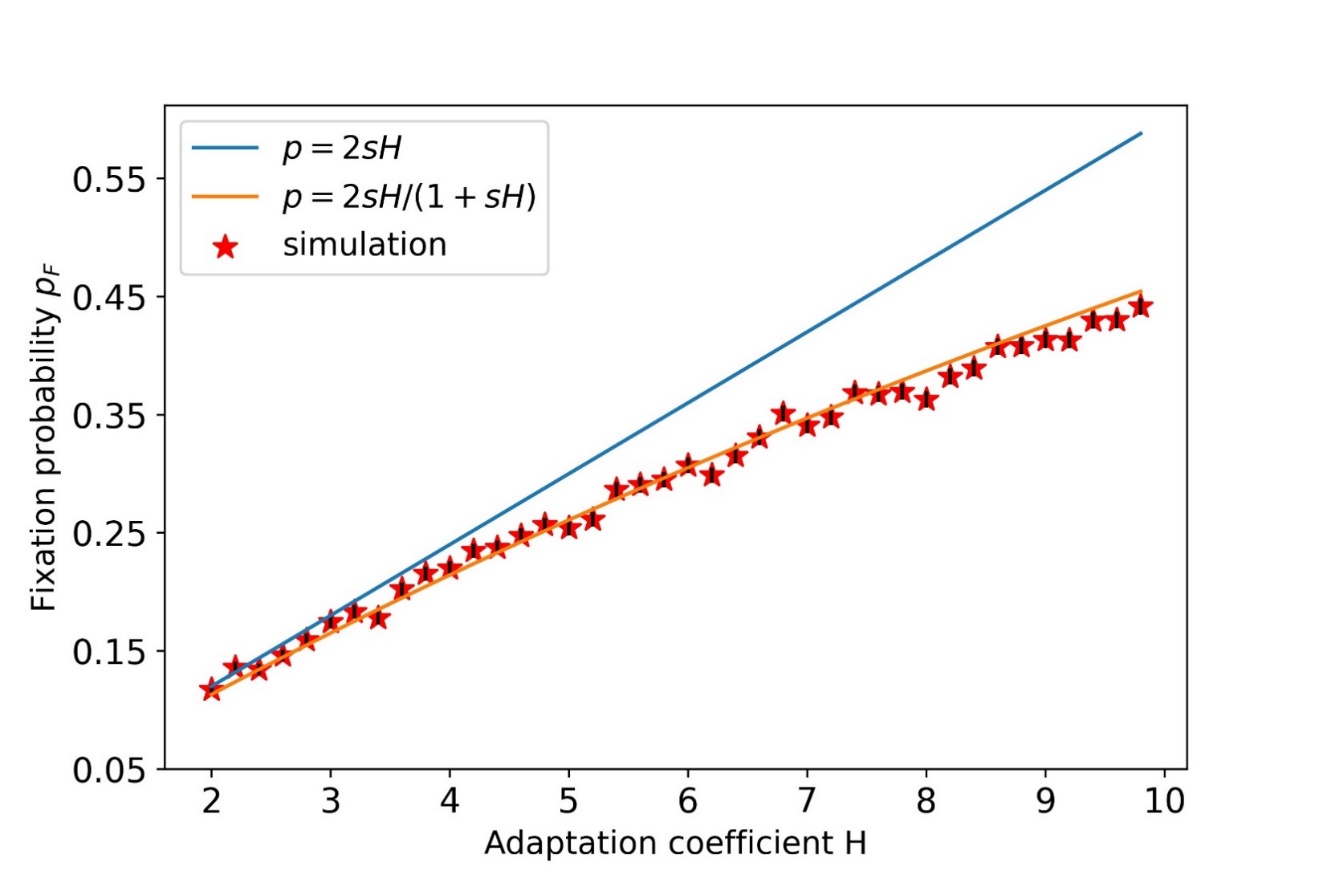


Figure S3. Fixation probability of a rare beneficial genotype. Red stars represent the frequency of fixations in n = 5000 events of adaptive genotype appearance. Error bars (too small to see) show the estimated error $\sqrt{{\boldsymbol{p}_{\boldsymbol{F}}\mathbf{(1-}\boldsymbol{p}_{\boldsymbol{F}}\mathbf{)}}/\boldsymbol{n}}$. The analytic approximation in Eq. A2a (orange) explains $\boldsymbol{R}^{\mathbf{2}}\mathbf{=0.9968}$ of the variance in the simulation results, while the approximation Eq. A2b (blue) explains $\boldsymbol{R}^{\mathbf{2}}\mathbf{=0.9919}$. Here, s = 0.03.


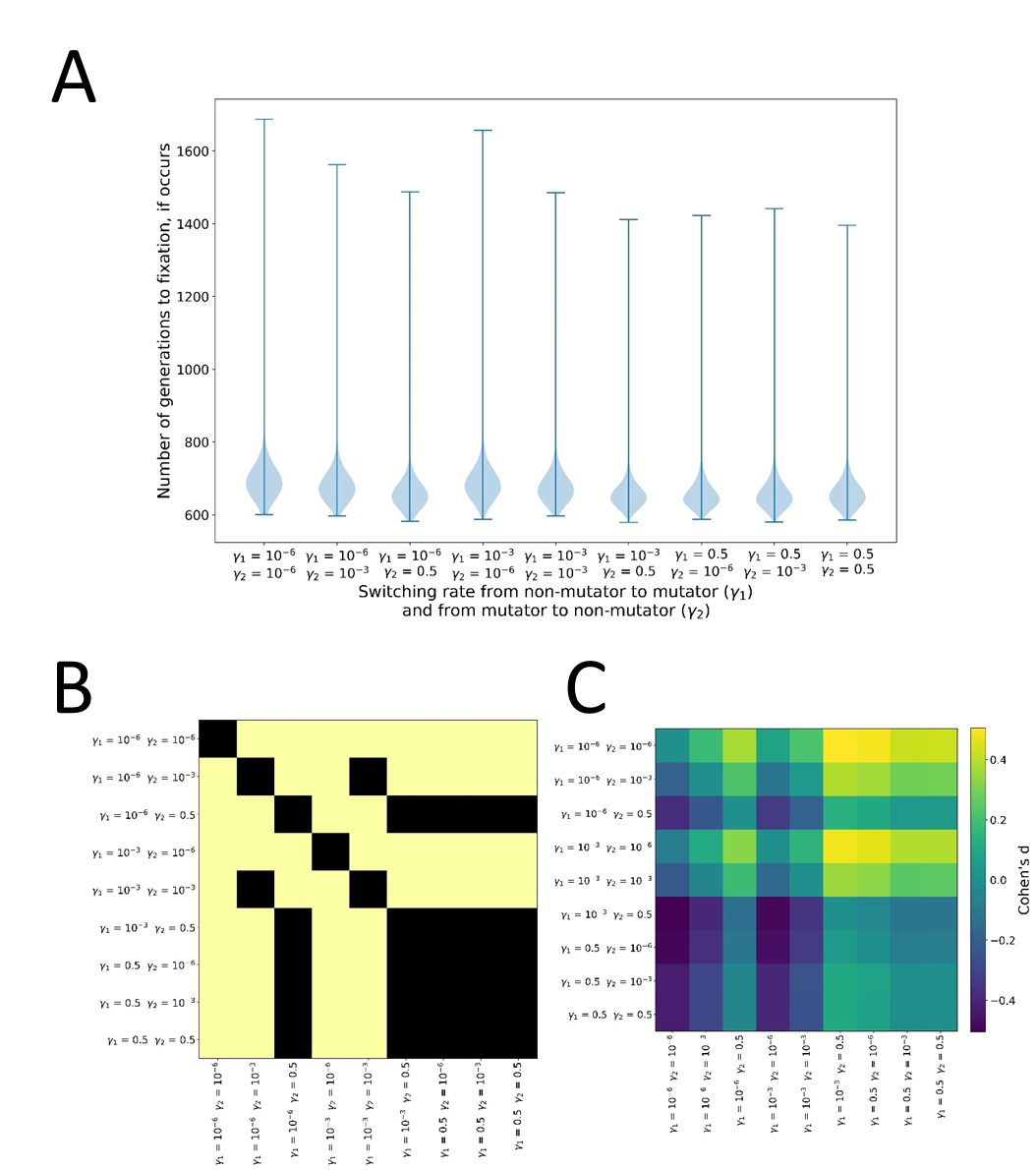


**Figure S4. (A) Time to fixation for pairs of** $\boldsymbol{\gamma}_{\mathbf{1}}$ **and** $\boldsymbol{\gamma}_{\mathbf{2}}$. The time to fixation follows a right-tailed distribution that is not strongly dependent on the values of $\gamma_{1}$ and $\gamma_{2}$. The adaptive mutant had a 6% advantage over the wild-type. **(B) Significance of all pairwise comparisons of time distributions.** A Mann-Whitney test was performed with significance threshold set at 0.01, and a Bonferonni correction was applied to correct for multiple comparisons. Distributions where one switching rate equals 0.5 seem to not be significantly different from one another. Yellow represents statistical significance, black represents lack of statistical significance. **(C) Effect size of the difference between the pairs of the time distributions**. Calculated with Cohen’s $d$. Maximal effect size is less than 0.5, which is quite small.


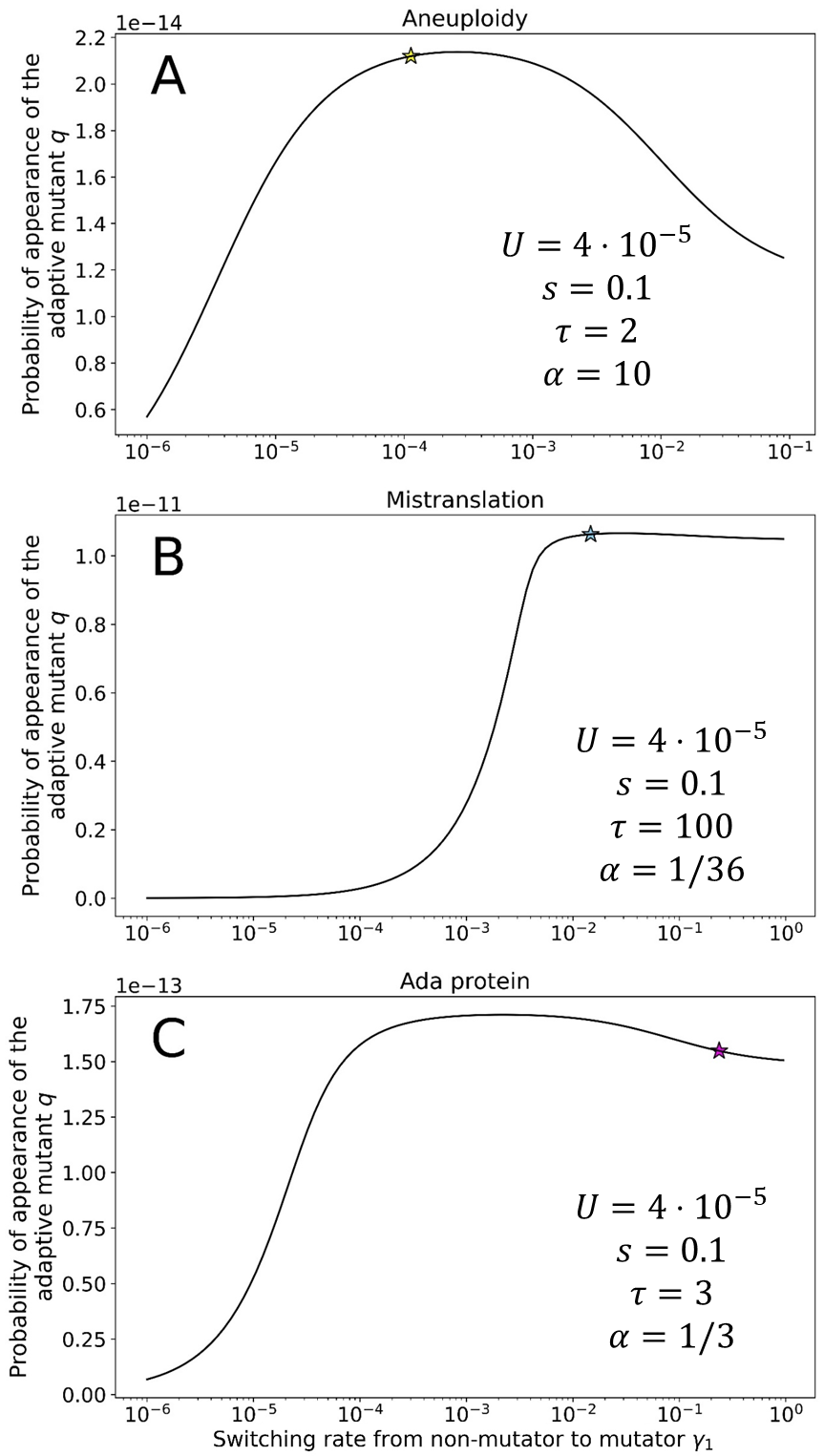


**Figure S5. Probability of appearance of the adaptive mutant** $\boldsymbol{q}$ **for the three empirically described systems for non-genetic inheritance of the mutation rate.** We estimated the specific values of the $\alpha$ and $\tau$ parameter for each of the three systems. The coloured star was plotted at its estimated value of $\gamma_{1}$ on the x-axis.


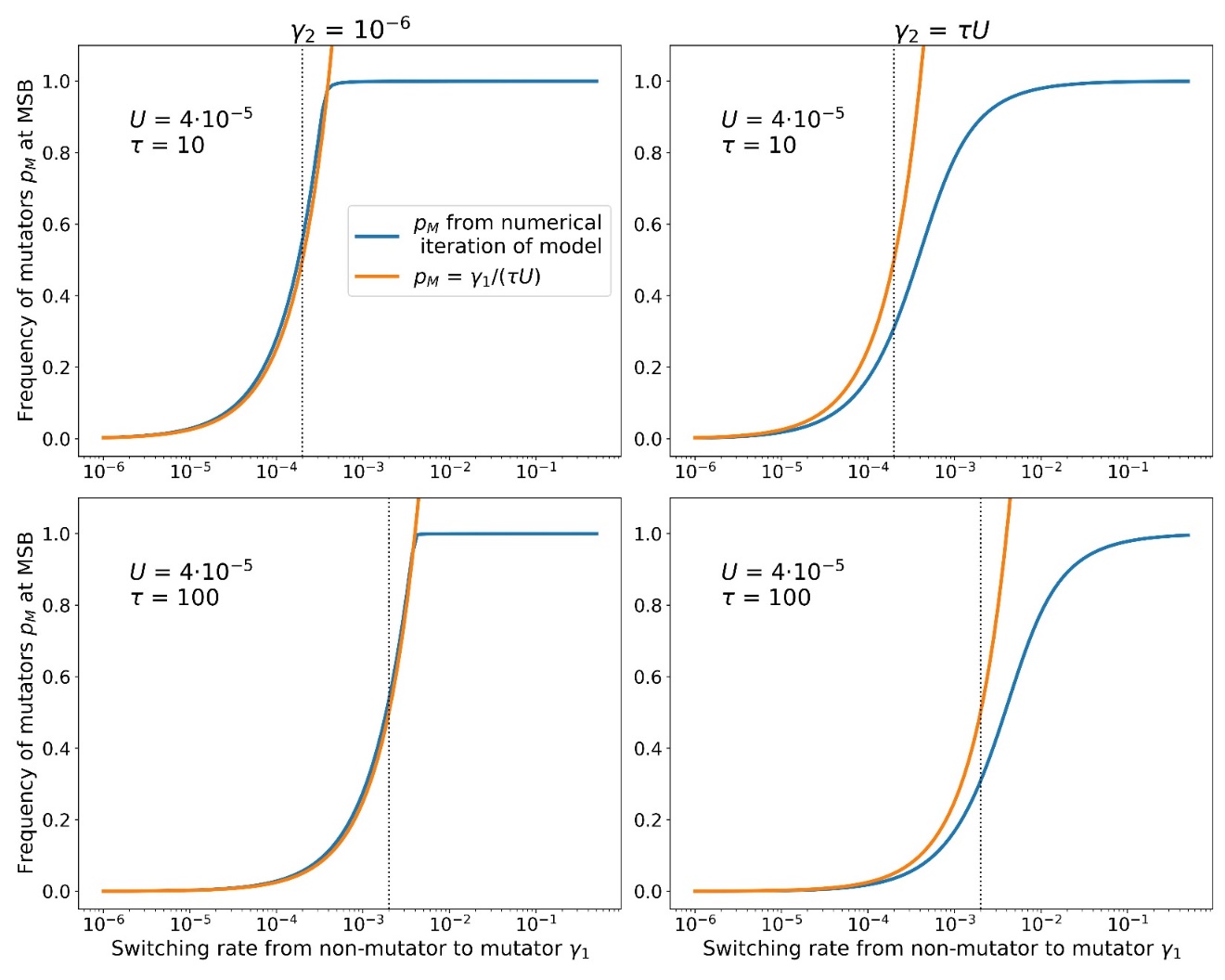


**Figure S6. Comparison of the proportion of mutators from the numerical simulation with the analytical prediction derived by** [23]**.** The two considered switching rates from mutator to non-mutator $\gamma_{2}$ correspond to genetic inheritance ($\gamma_{2}={10}^{-6}$) and the highest value of $\gamma_{2}$ for which we use Eq. 4 ($\gamma_{2}=\tau U/2$). The dashed line corresponds to $\gamma_{1}=\tau U/2$. For $\gamma_{2}={10}^{-6}$, we observe an excellent fit for all considered parameter sets. For $\gamma_{2}=\tau U/2$, the fit worsens progressively with increasing switching rate from non-mutator to mutator $\gamma_{1}$. Parameters: $s=0.03$, $n=5000$.

**
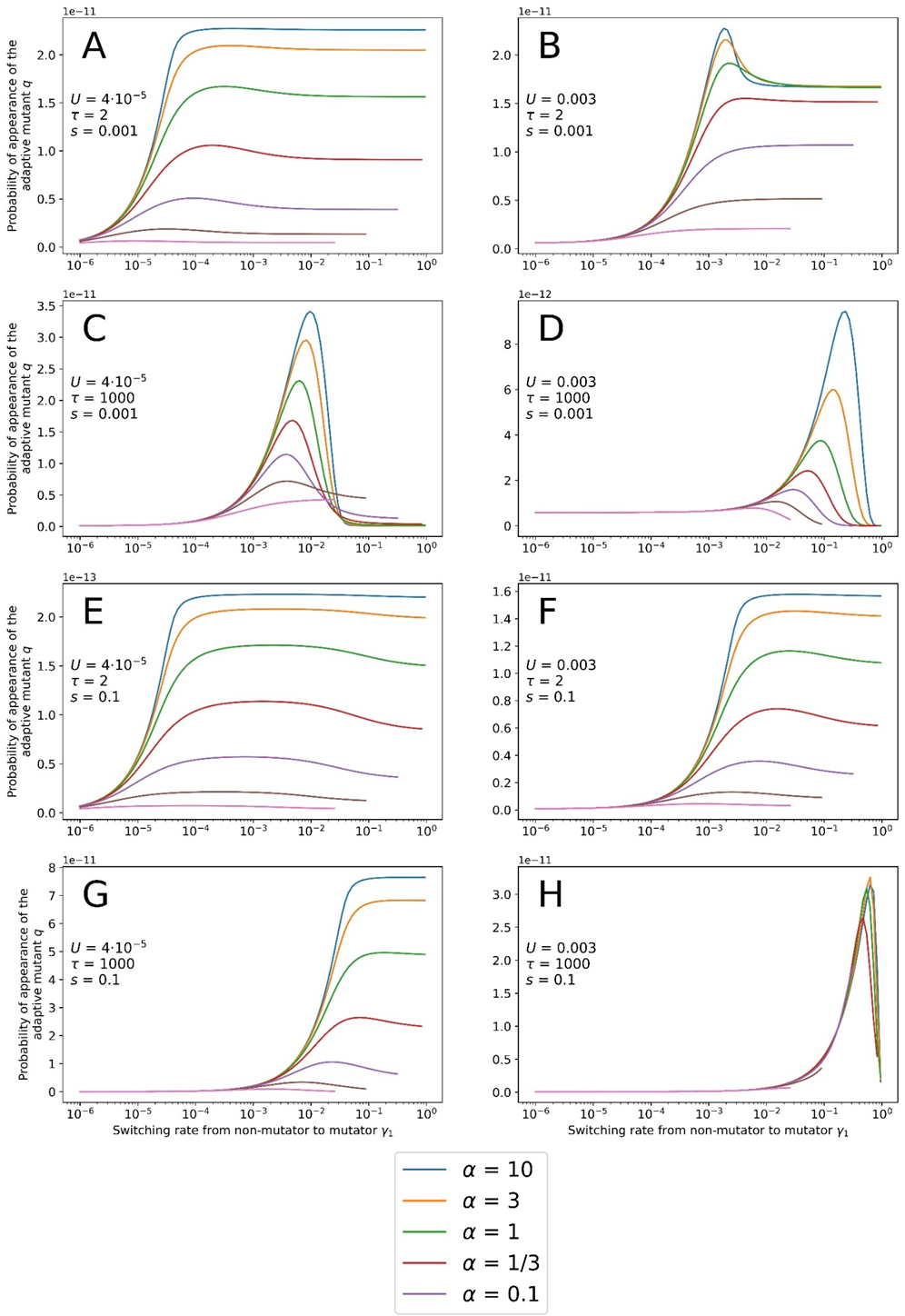
**

**Figure S7. Probability of appearance of the adaptive genotype** $\boldsymbol{q}$ **for extreme values of** $\boldsymbol{U}$**,** $\boldsymbol{\tau}$**, and** $\boldsymbol{s}$**.**  The probability of appearance was calculated with Eq, 5 and the MSB frequencies obtained for each parameter set.

**
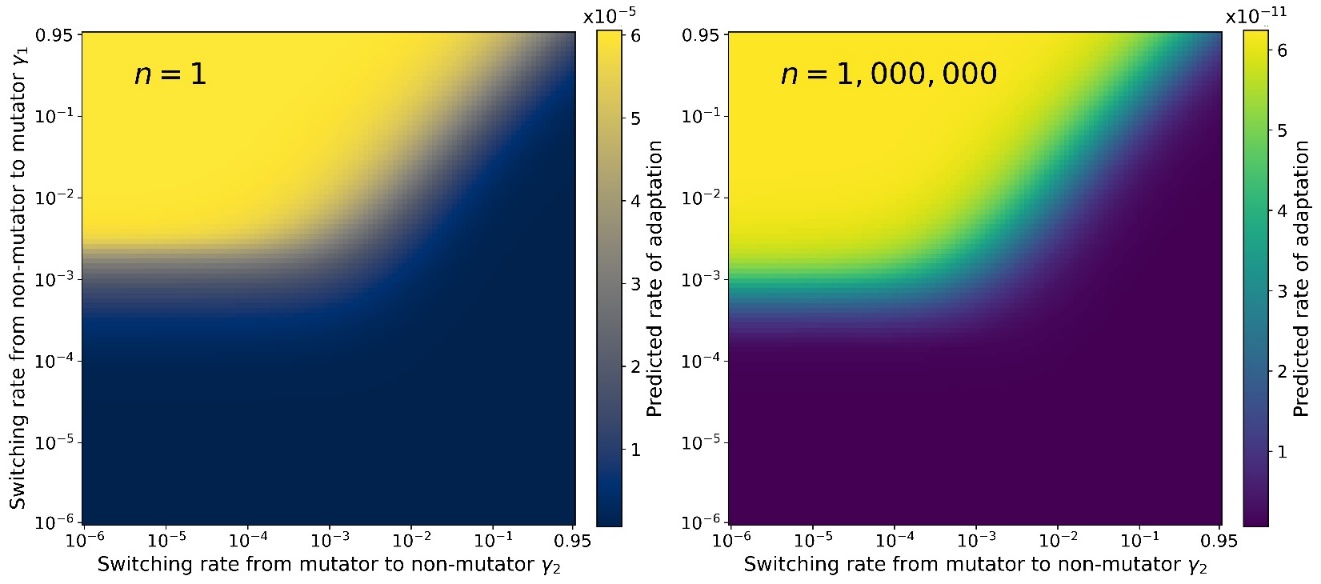
**

**Figure S8. Sensitivity analysis for the number of loci** $\boldsymbol{n}$**.** Although the rate of adaptation is 6 orders of magnitude lower for $n=1,000,000$, the relative rate of adaptation is similar for the two values of $n$. Indeed, the highest rates of adaptation are observed for $\gamma_{1}>\frac{\tau U}{2}$ when $\gamma_{2}<\frac{\tau U}{2}$ and for $\gamma_{1}>\gamma_{2}$ when $\gamma_{2}>\frac{\tau U}{2}$. $U=0.0001$, $s=0.03$, $\tau=10$.


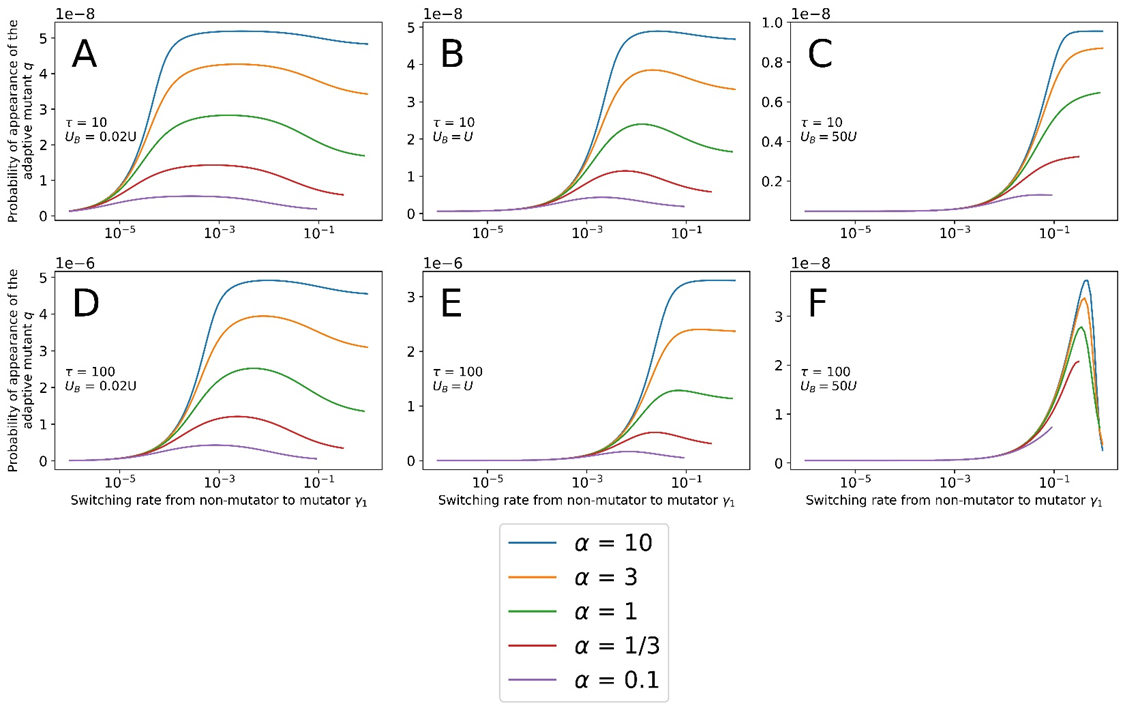


**Figure S9. The adaptation-optimal switching rate increases with the deleterious mutation rate, but drops sharply above a critical threshold.** Each plot shows the probability of appearance rate of the adaptive mutant $q$ along the switching rate from non-mutator to mutator $\gamma_{1}$. Parameters: $U=4\cdot{10}^{-4}$, $s=0.1$, $n=5,000$.


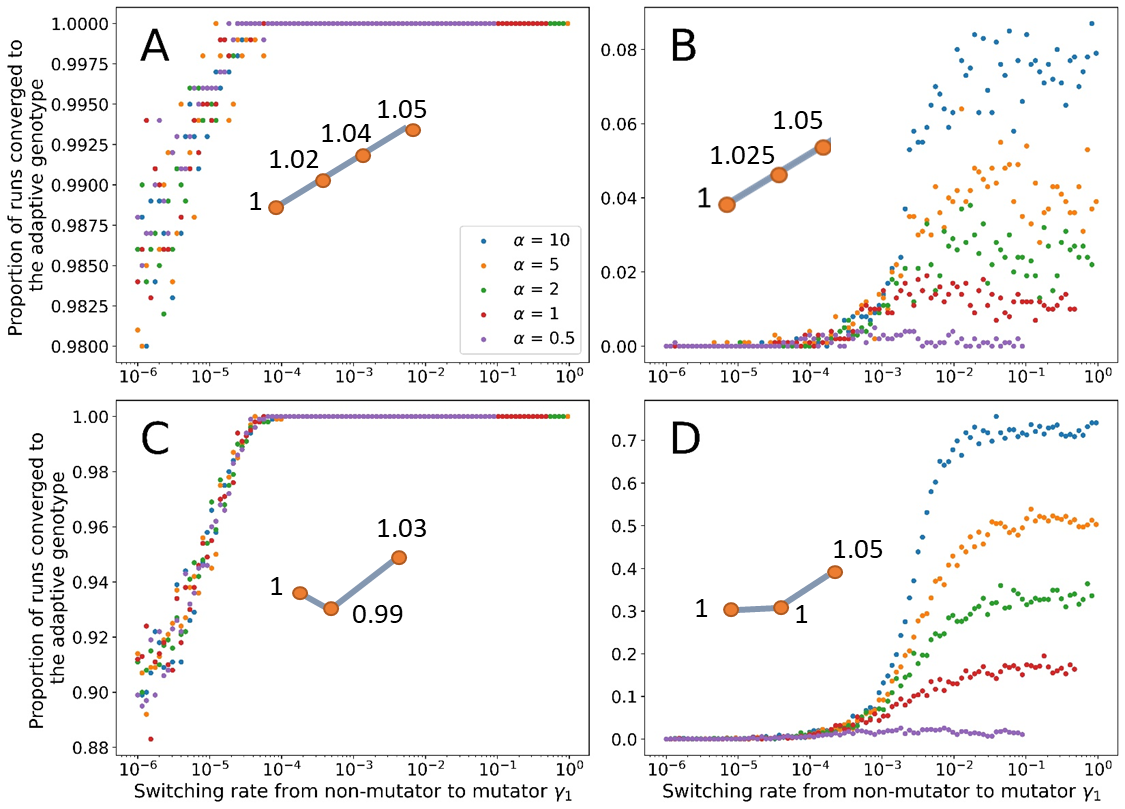


**Figure S10: Adaptation rate for several fitness motifs**. Same as Figure 5, but for additional fitness landscape motifs.


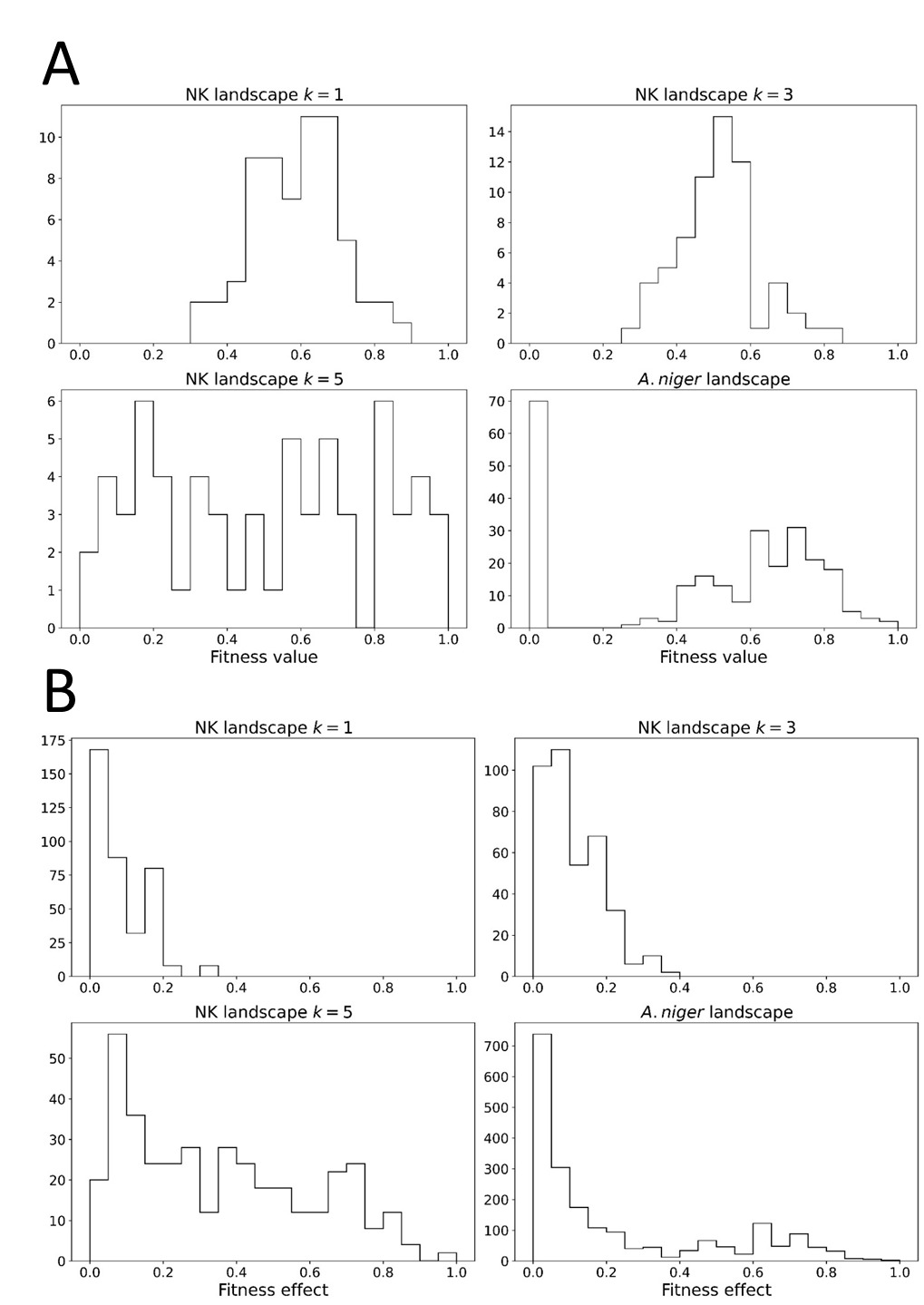


Figure S11: (A) Distribution of fitness values in the NK and *Aspergillus niger* landscapes. In the NK landscapes, the higher the $\boldsymbol{k}$ (representing the ruggedness), the wider the distribution of fitness values. (B) Distribution of fitness effects in the NK and *Aspergillus niger* landscapes. A histogram of the mean difference between each genotype and its single mutants for different values of the ruggedness parameter $\boldsymbol{k}$. As expected, as $\boldsymbol{k}$ increases, the distribution is wider.


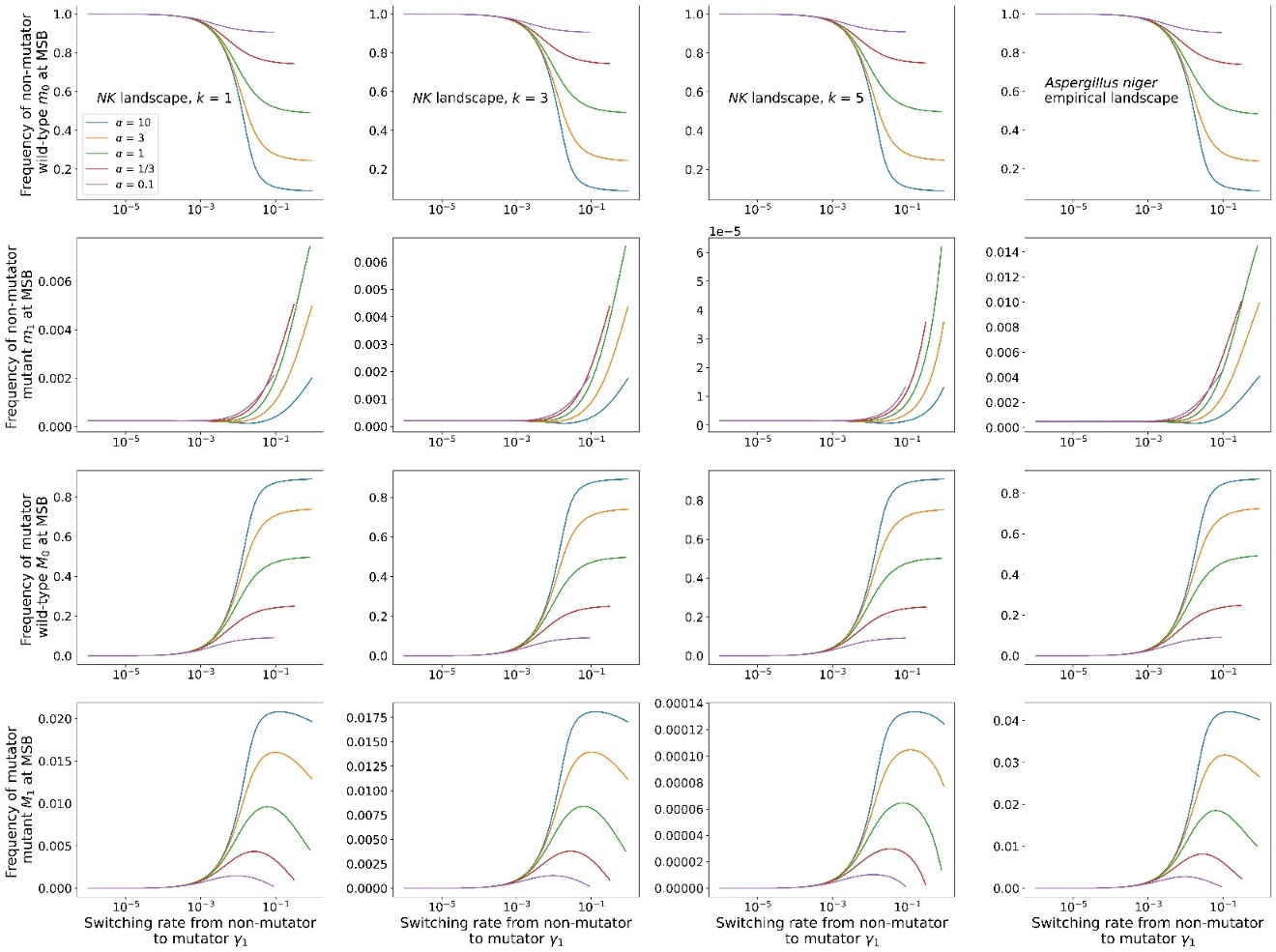


**Figure S12: Frequencies of non-mutator wild-type, non-mutator mutant, mutator wild-type, and mutator mutant at MSB for two values of the mutation rate** $\boldsymbol{U}$ **and two value of the fold-increase in mutator mutation rate** $\boldsymbol{\tau}$**, for the four considered complex landscapes: the *Aspergillus niger* empirical landscape, and three *NK* landscapes of varying ruggedness.** Parameters: $U=4\cdot{10}^{-5}$, $\tau=100$.

**
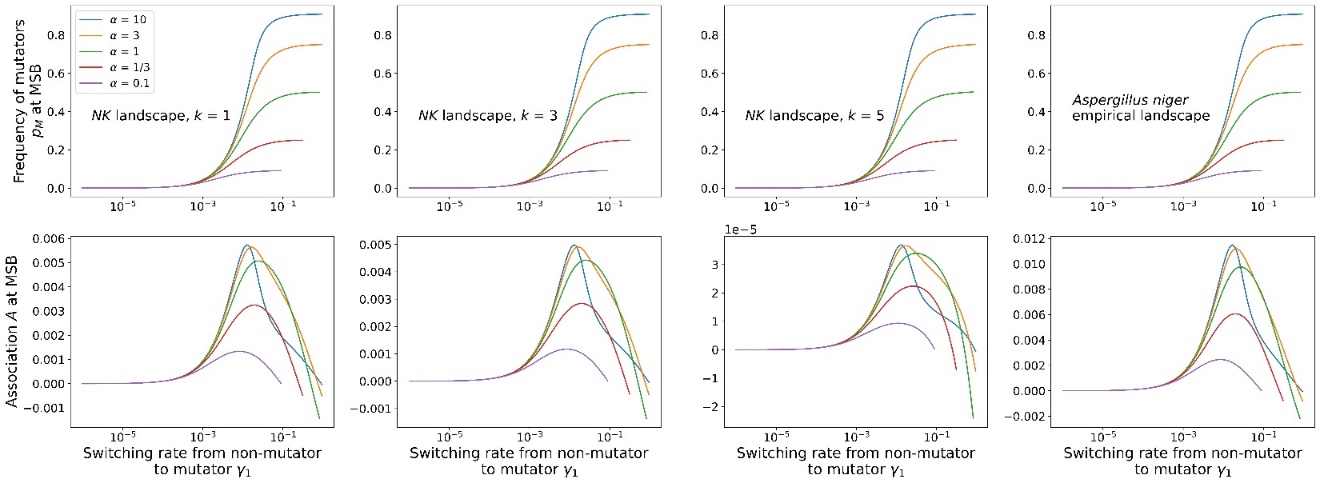
**

**Figure S13: Frequency of mutators** $\boldsymbol{p}_{\boldsymbol{M}}$ **at MSB and association** $\boldsymbol{A}$ **at MSB for the four considered complex landscapes: the *Aspergillus niger* empirical landscape, and three *NK* landscapes of varying ruggedness.** The proportion of mutators $p_{M}$ is obtained directly from the MSB frequencies. The association $A$ is calculated from the MSB frequencies according to Eq. 8. Parameters: $U=4\cdot{10}^{-5}$, $\tau=100$.


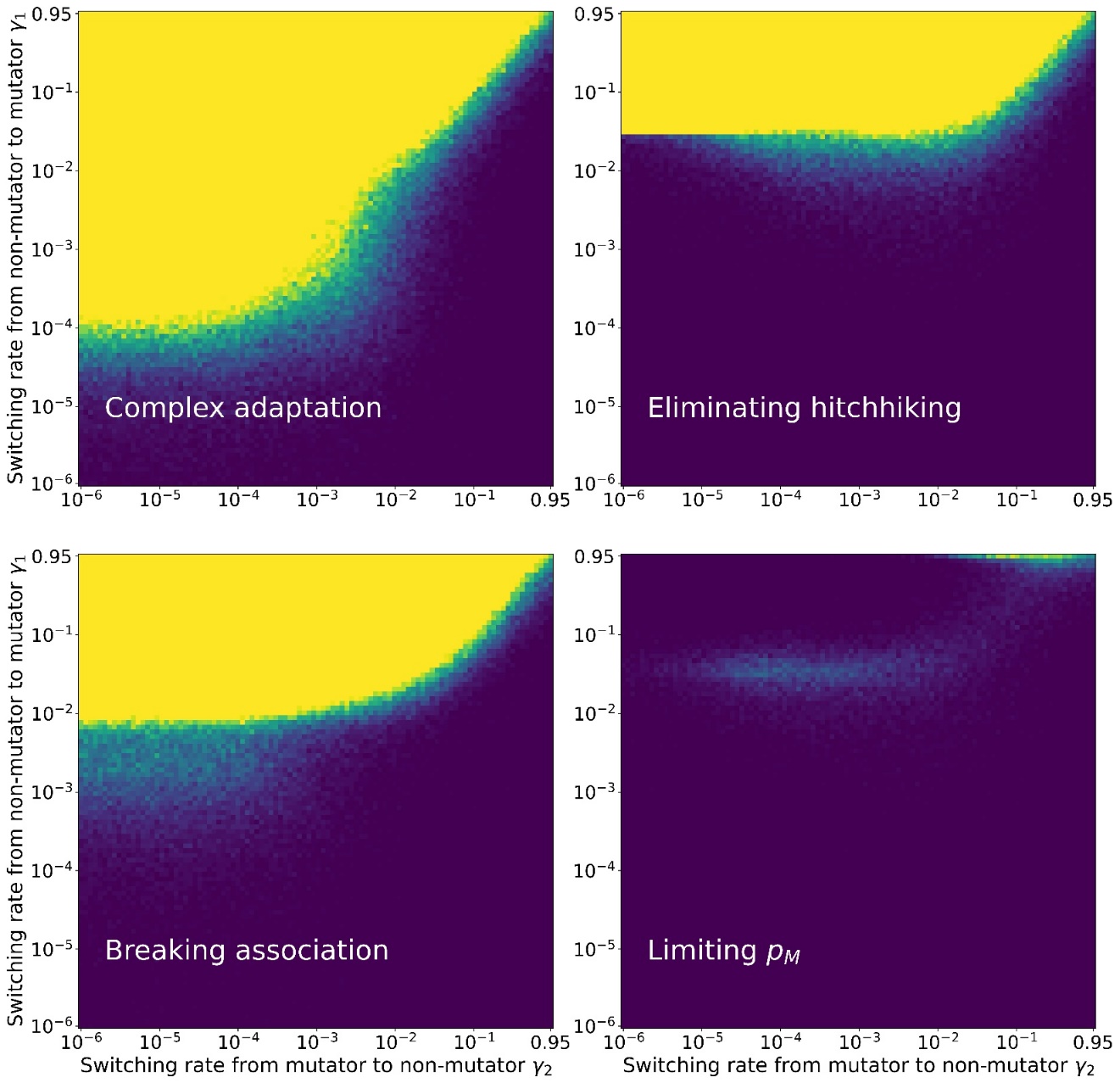


**Figure S14: Complex adaptation on *Aspergillus niger* landscape for all combinations of values of the two switching rates** $\boldsymbol{\gamma}_{\boldsymbol{1}}$ **and** $\boldsymbol{\gamma}_{\boldsymbol{2}}$**.** Same as Figure 6, but for all possible values of $\gamma_{1}$ and $\gamma_{2}$. Parameters: $U=4\cdot{10}^{-5}$, $\tau=100$, $N=1000$.


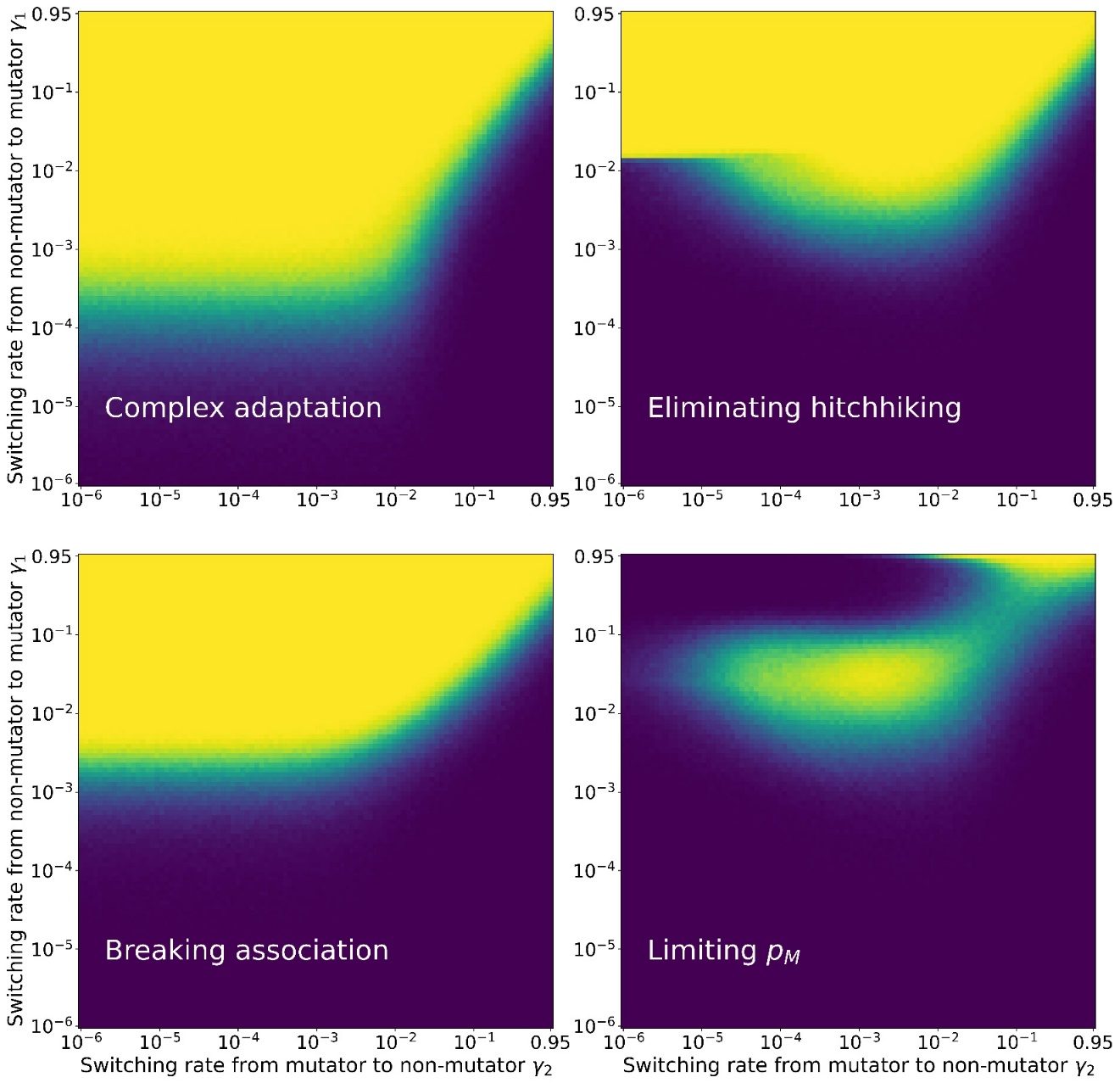


**Figure S15: Complex adaptation on NK landscape, *k=1*.** The proportion of runs out of 1000 that converged upon the fittest genotype in the landscape was recorded after 500 generations. In order to disentangle the different effects of the non-genetic inheritance of the mutation rate on the rate of adaptation, we then rerun the simulation while removing the association of mutator and mutant, limiting the frequency of mutators during the evolution, and eliminating hitchhiking. Note that hitchhiking is also eliminated when the association between mutator and mutant is broken, or the frequency of the mutator limited. We observe that the region with rates of adaptation for $\gamma_{1}<\tau U/2$ and $\gamma_{2}<\tau U/2$ disappears when hitchhiking is eliminated. Limiting the frequency $p_{M}$ reduces adaptation overall. Parameters: $U=4\cdot{10}^{-5}$, $\tau=100$, $N=1000$.


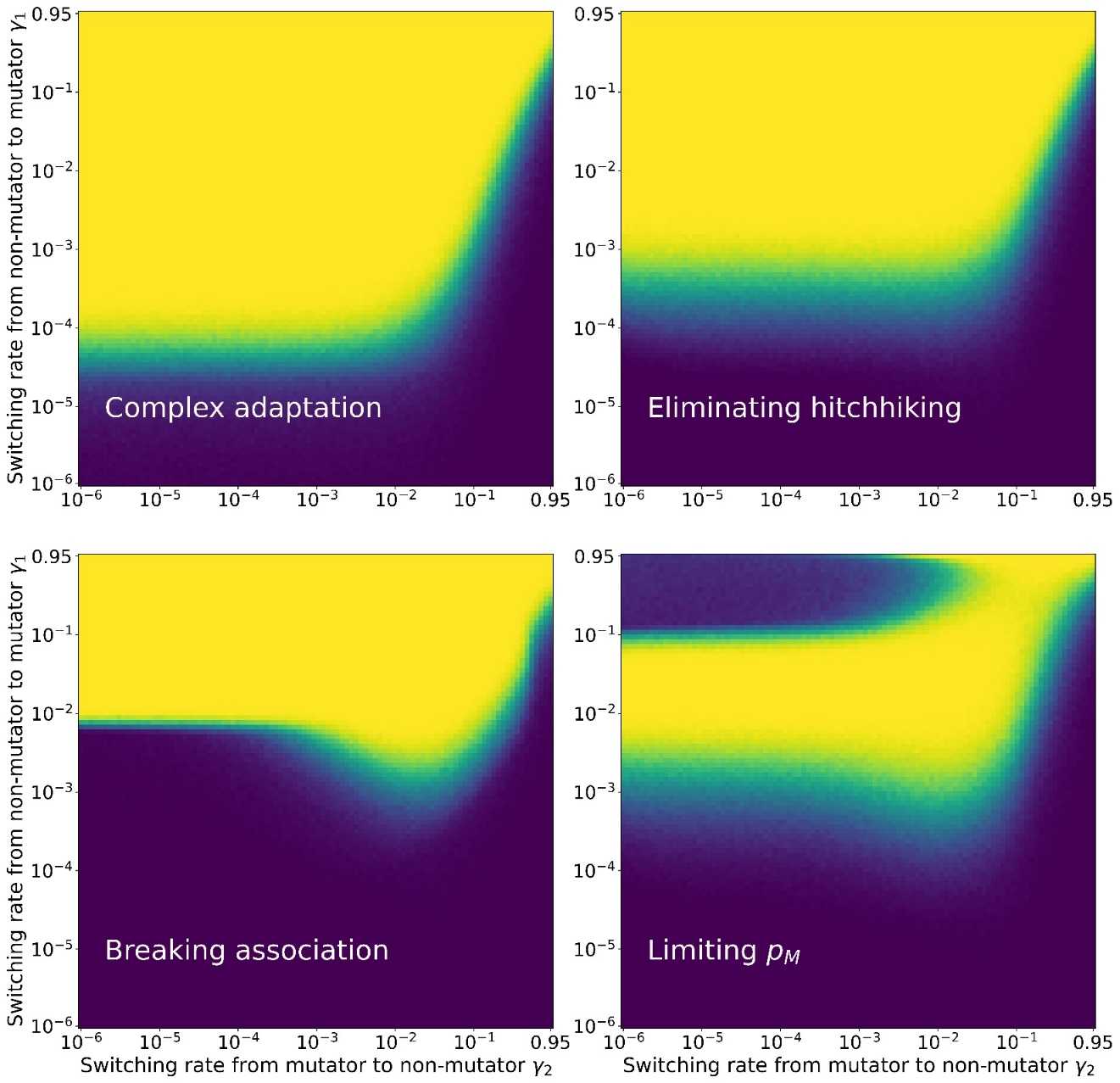


**Figure S16: Complex adaptation on NK landscape, *k=3*.** Same as Figure S15, but for *k=3*. Parameters: $U=4\cdot{10}^{-5}$, $\tau=100$, $N={10}^{7}$.


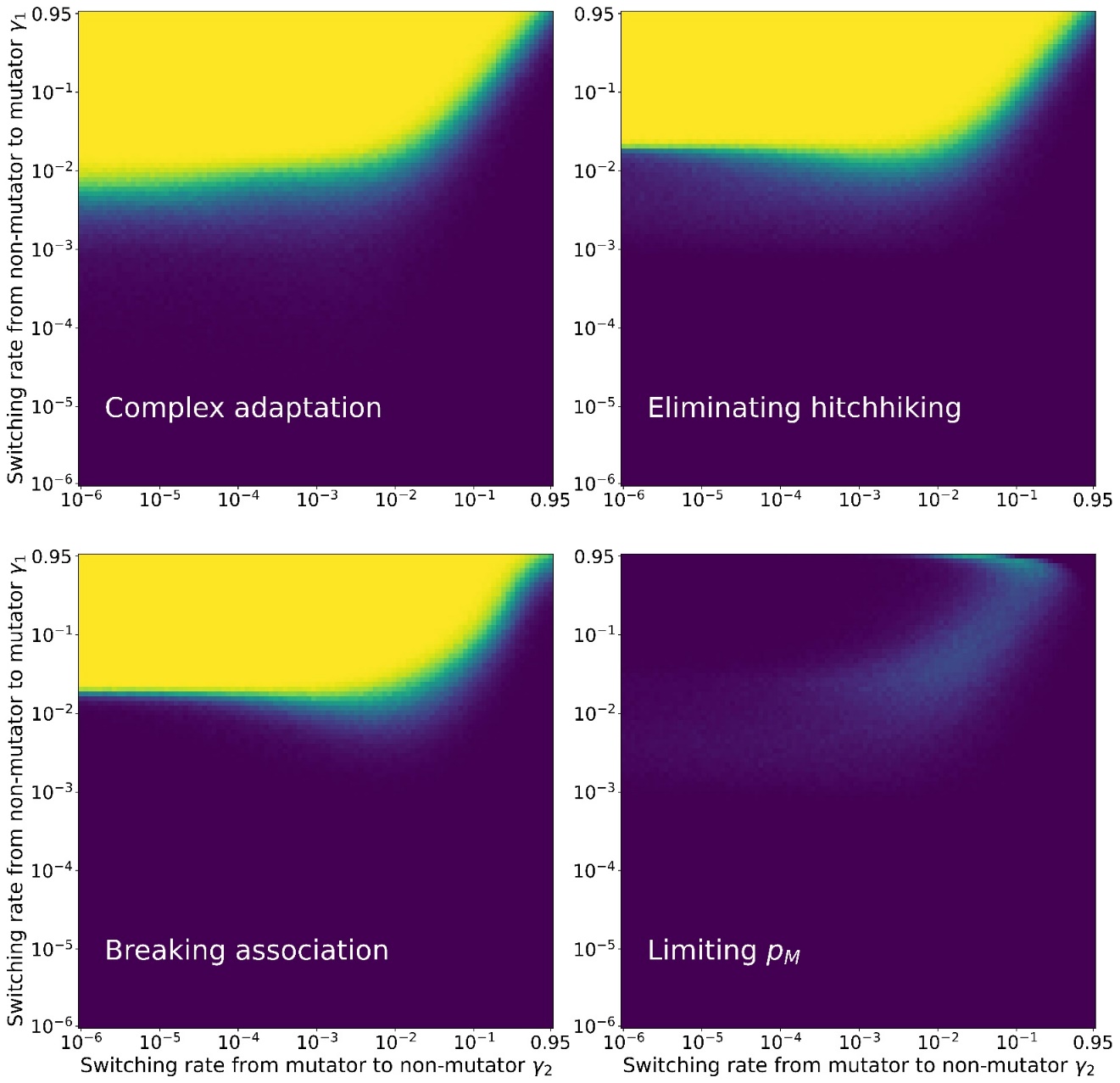


**Figure S17: Complex adaptation on NK landscape, *k=5*.** Same as Figure S15, but for *k=5*. Parameters: $U=4\cdot{10}^{-5}$, $\tau=100$, $N={10}^{7}$.


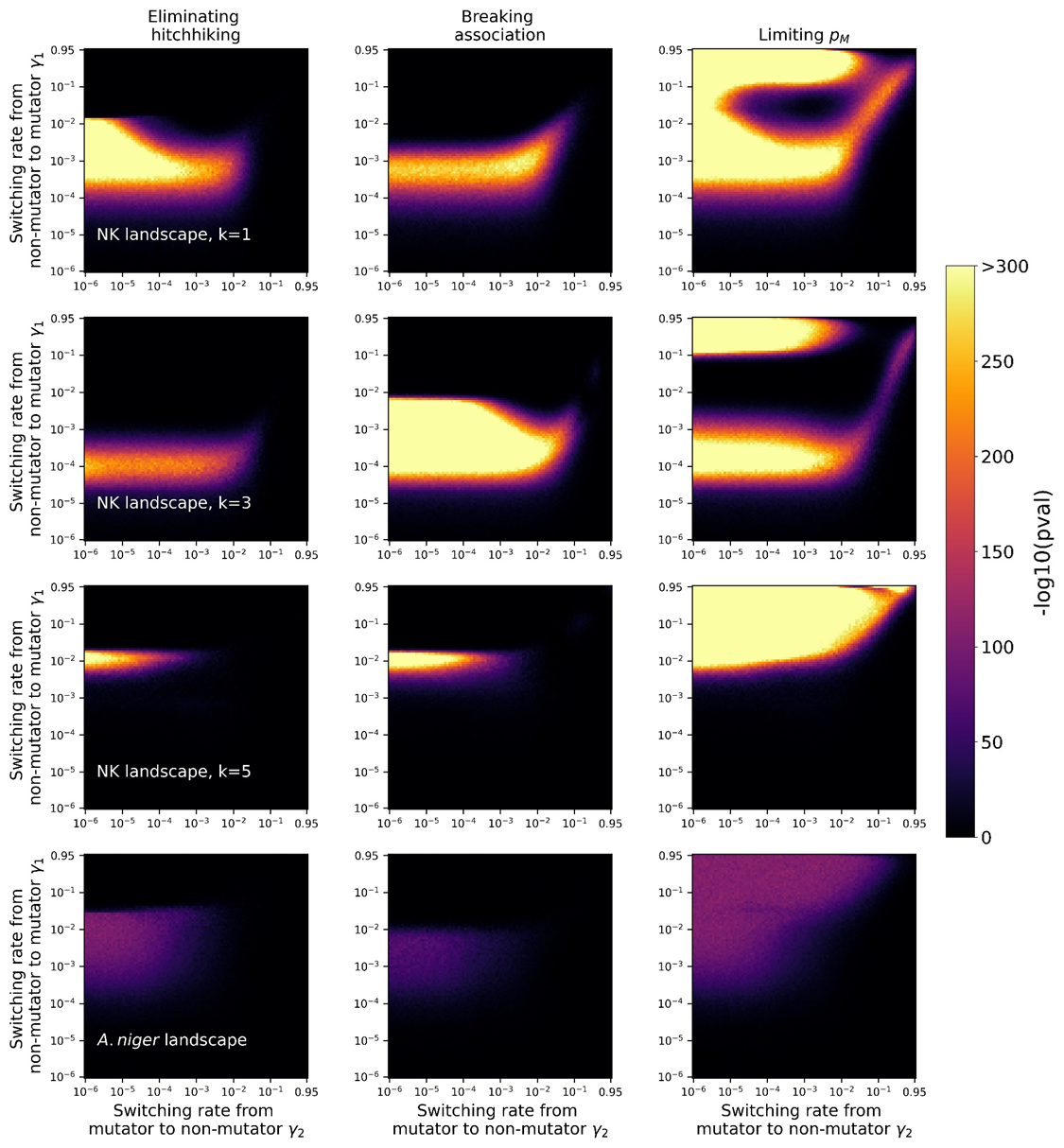


**Figure S18: Statistical analysis of the rate of adaptation in the complex adaptation simulation and its modifications: eliminating hitchhiking, reducing the proportion of mutators and breaking association between mutators and mutants.** A two-proportion Z-test was performed between the proportion of runs that converged on the adaptive genotype in the complex adaptation simulation and between the proportion of runs that converged on the adaptive genotype in a modification of the simulation. We report the –log10 of the obtained p-value.
