# Supplementary material for: Phenotype switching of the mutation rate facilitates adaptive evolution
Source: Genetics. 2023 Jun 9;225(1):iyad111. doi: 10.1093/genetics/iyad111 (PMC10471227; doi:10.1093/genetics/iyad111)
Supplement: iyad111_Supplementary_Data [file iyad111_supplementary_data.pdf]

Appendix to: Phenotype switching of the mutation rate facilitates adaptive evolution

Gabriela Lobinska[1], Yitzhak Pilpel[1*], Yoav Ram[2*]

[1] Department of Molecular Genetics, Weizmann Institute of Science, Rehovot 76100 Israel

[2] School of Zoology, Faculty of Life Sciences, Tel Aviv University, Tel Aviv 6997801 Israel

* Correspondence: pilpel@weizmann.ac.il, yoav@yoavram.com

A. The Wright-Fisher model

The Wright-Fisher model is commonly used to model evolutionary biology. In this model, a population is described by a probability vector that contains the frequency of each genotype that appears in the population. This population vector is modified by iterating mutation-selection-drift steps. In the mutation step, the population vector is multiplied by a transition matrix that gives the probability of mutation between each pair of genotypes. In the selection step, the frequency of each genotype is multiplied by its relative fitness, and then the population vector is normalized so that it sums to one by dividing with the population mean fitness. In the drift step, stochasticity is introduced by randomly sampling the population vector of the next generation from a multinomial distribution characterize by the population vector of the current generation after the mutation and selection steps. One mutation-selection-drift cycle corresponds to a single generation of the population [1].

B. Discussion about fitness landscapes.

Biological fitness landscapes are notoriously difficult to investigate due to their high dimensionality. Indeed, a DNA sequence of length N corresponds to 4^N genotypes, and a protein sequence of length N corresponds to 20^N possible proteins. Considering the average length of a gene, or that of a protein, it quickly becomes obvious that all possible sequences cannot be possibly surveyed empirically. Existing experimental studies of fitness landscapes have focused either on local landscapes around proteins [2], [3] or short DNA sequences [4]. Traditionally, fitness landscapes were described as containing many "valleys", corresponding to low fitness genotypes, and "peaks", corresponding to high fitness genotypes. Therefore, a central problem in evolutionary biology was to explain how fitness valleys can be crossed when natural selection acts to eliminate low-fitness intermediate genotypes [5]. A few examples of fitness valley crossing have been suggested [6], [7], along with strategies for their crossing such as capacitance [8], [9], partial robustness [10], stress-induced mutagenesis [11], or phenotypic variation [12]. Yet, due to the hyper-dimensionality of biological fitness landscapes, fitness valleys could actually rarely exist, and fitness landscapes may thus be more highly navigable than often appreciated [13]. Hence, evolutionary adaptation over rugged landscapes would be more akin to a diffusion problem in which adaptation is dependent on the time needed to find a mutational path that does not contain a fitness valley [14]. The empirical evidence for

either an abundance or lack of fitness valleys is scarce, as observing fitness valley crossings poses technical difficulties due to the very low frequency of the low fitness intermediate genotype.

C. Simple landscape

The mutation transition probability is $u_{j \to g}$, where *j* and *g* are genotypes, determines the effect of mutation on the genotype probabilities (Eq. 1). The construction of the mutation transition probability is described in the main text. Here, we provide a formal description. Note that we assume no back mutations occur. In the following we use the mutator phenotype mutation rate τU; for the non-mutator, we set $\tau = 1$. The per-locus mutation rate is $\mu = U/n$.

The number of pre-existing background mutations in the source genotype is denoted by k. The number of background mutations acquired during the mutation step is denoted by l. Hence, the probability of transition from genotype $0\backslash 1$ to genotype $0\backslash 3$ corresponds to $k = 1$ and $l = 2$.

Thus, the probability to transition from genotype *j* to genotype *g* for the mutator genotype, $u'_{j \to g}$, is described by **Table A1**, where the source genotype *j* is given in the row, and the target genotype *g* is given in the column.

$u'_{j \to g}$	$g = 0\backslash(k+l)$	$g = 1\backslash(k+l)$	$g = 2\backslash(k+l)$
$j = 0\backslash k$	$(1-\tau\mu)^2 \cdot \frac{e^{\tau U}(\tau U)^l}{l!}$	$2\tau\mu(1-\tau\mu) \cdot \frac{e^{\tau U}(\tau U)^l}{l!}$	$(\tau\mu)^2 \cdot \frac{e^{\tau U}(\tau U)^l}{l!}$
$j = 1\backslash k$	0	$(1-\tau\mu) \cdot \frac{e^{\tau U}(\tau U)^l}{l!}$	$\tau\mu \cdot \frac{e^{\tau U}(\tau U)^l}{l!}$
$j = 2\backslash k$	0	0	$\frac{e^{\tau U}(\tau U)^l}{l!}$

Table A1. Probabilities to transition from genotype *j* to genotype *g*, $u'_{j \to g}$.

D. Description of complex landscapes

NK landscapes

The NK landscape is commonly used in the study of epistatic interactions [15]. It has two parameters: n for the number of bi-allelic loci in the genotype and k for the number of loci each locus interacts with. The main advantage of the NK landscape is its biological interpretation and its tunable ruggedness [16]. Here, the genotype consists of $n = 6$ bi-allelic loci, which results in $2^6 = 64$ genotypes.

We chose $n = 6$ in order to allow for multidimensionality, while maintaining reasonable computation times.

To construct an NK landscape, we first generate all possible k-bit strings (bit strings of length k) and assign to each of them a random fitness value between 0 and 1, sampled from a continuous uniform distribution. Genotype g is a n-bit string, and its fitness w_g is the sum of the fitness effects of the k-bit strings that g contains. A locus thus influences the genotype fitness according to the number of k-bit strings that contain it, which increases with k. Hence, the ruggedness of the NK landscape increases with k [17]. We consider three NK landscapes with low, intermediate, and high ruggedness corresponding respectively to k = 1, k = 3, and k = 5. See **Figure S11** for properties of the constructed landscapes.

Empirical fitness landscape from *Aspergillus niger*

We examine the insights gained on two-peak and NK landscapes with an empirical fitness landscape [15] measured with mutants in 8 genes of *Aspergillus niger* [17]. We chose this landscape because: (i) it is complete with fitness measurements of all possible 256 genotype combinations, so we can avoid the interpolation of fitness values of missing genotypes; and (ii) fitness was measured as growth rate relative to the wild type, as opposed to other studies that quantify some proxy phenotype for fitness such as fluorescence or DNA binding [4], [18], [19].

The genotype consists of eight bi-allelic loci, each with either the wild type or the mutant allele. de Visser *et al.* [17] engineered 256 genotypes to bear all possible combinations of wild type/mutant alleles in these eight loci. The loci are: *fwnA1* (fawn-colored conidiospores); five auxotrophic markers, *argH12* (arginine deficiency), *pyrA5* (pyrimidine deficiency), *leuA1* (leucine deficiency), *pheA1* (phenyl-alanine deficiency),

and *lysD25* (lysine deficiency); and two resistances, *oliC2* (oligomycin resistance) and *crnB12* (chlorate resistance).

de Visser *et al.* [17] estimated fitness by measuring the growth rate of each strain relative to the growth rate of the wild type strain (i.e., with eight wild type alleles). Out of 256 genotypes, 70 genotypes were lethal (fitness is zero). The landscape is rugged: it contains 15 local maxima (including the global maximum). See **Figure S11** for properties of the landscape.

E. Calculating the switching rate from mutator to non-mutator γ_2 for the Ada protein

We define a Markov chain with two states: cells with zero Ada molecule denoted by *x* and cells with non-zero Ada molecule denoted by *y*.

The transition matrix T is given by

$$T = \begin{pmatrix} p_{x\to x} & p_{x\to y} \\ p_{y\to x} & p_{y\to y} \end{pmatrix}$$

where $p_{x\to x} = 1 - p_{x\to y}$ and $p_{y\to y} = 1 - p_{y\to x}$. In [20], the probability of transitioning from zero to one (or more) Ada molecules is fitted to a Poisson distribution with average equal to 1. Hence, we have $p_{x\to x} = 0.37$ and $p_{x\to y} = 1 - p_{x\to x} = 0.63$.

The stationary distribution of this Markov chain is denoted by (x^*, y^*) with $y^* = 1 - x^*$. Empirical evidence [20] suggests that $(x^*, y^*) \approx (0.25, 0.75)$. To estimate $p_{y\to y}$, we solve

$$x^* = p_{x\to x}\, x^* + p_{y\to x}\, y^*$$

$$y^* = p_{x\to y}\, x^* + p_{y\to y}\, y^*$$

to obtain

$$p_{y\to y} = 1 - \frac{p_{x\to y}\, x^*}{y^*}$$

Hence, the complete transition matrix is

$$T = \begin{pmatrix} 0.37 & 0.63 \\ 0.21 & 0.79 \end{pmatrix}$$

F. Bounds on the population size

We consider N, the population size, to be large enough so that individuals with a single mutation in the major loci are present, but small enough so that individuals with two mutations in the major loci (hence already adapted to the new environment) are absent.

Let us first consider the first condition. The lowest frequency of mutants is achieved when the whole population is non-mutator. Therefore, the upper bound for the first condition is set by considering a case where all individuals have the non-mutator phenotype. According to [21], the frequency of wild type individuals at mutation-selection balance is $e^{-U/s}$. We then multiply by the mutation-selection balance of single mutants at the major loci, $\frac{\mu}{s}$. Thus, we find that the expected number of single mutant individuals without deleterious mutations in the background loci is $N\frac{\mu}{s}e^{-U/s}$. Setting this to be less than one and rearranging, we obtain $\frac{s}{\mu}e^{U/s} < N$.

Now let us consider the second condition. The highest frequency of double mutants would be achieved when the whole population is mutator. Therefore, the lower bound for the second condition is set by considering a case where all individuals have the mutator phenotype. By the same argument as for the first condition, we find $N < \left(\frac{s}{\tau\mu}\right)^2 e^{\frac{\tau U}{s}}$ with $e^{\frac{-\tau U}{s}}$ the proportion of wildtype individuals in an all-mutator population and $\left(\frac{\tau\mu}{s}\right)^2$ the frequency of double mutants in the major loci, both at a mutation selection balance.

Combining these conditions, we have

$$\frac{s}{\mu}e^{U/s} < N < \left(\frac{s}{\tau\mu}\right)^2 e^{\frac{\tau U}{s}} \quad \text{(A1)}$$

G. Fixation of an adaptive genotype

We set the fitness of the double mutant to be $1 + sH$, where H is the adaptation coefficient. According to Eshel [22], in a large population with weak selection, the fixation probability ρ_F of a double mutant once it appears in a single copy depends only on the selection coefficient s and the double mutant advantage H, namely

$$\tilde{\rho}_F = \frac{2sH}{1 + sH} \quad \text{(A2a)}$$

$$\approx 2sH, \quad \text{(A2b)}$$

where Eq. A2b applies when sH is much smaller than 1.

Figure S6 shows a comparison of the two approximations in eq. A2 to results from stochastic simulations obtained by counting the number of fixation events after the appearance of an adaptive genotype**.**

We notice that Eq. A2b is independent from the switching rate γ. Hence, the adaptation rate will be proportional to the appearance rate (see **Figure S3**). We therefore consider the appearance rate as a proxy for the adaptation rate in the main text.

H. Complex landscapes: breaking the association between mutators and mutants

The stochastic model was run following the usual mutation – phenotypic switching – selection – drift scheme. Right before the drift step, the population vector was recalculated as follows:

$$M_1' = \max(p_S \cdot p_M, 0)$$

$$m_1' = \max(p_S \cdot (1 - p_M),0)$$

$$M_0' = \max\left((1 - p_S) \cdot p_M, 0\right)$$

$$m_0{}' = \max(1 - M_1' - m_1' - M_0', 0)$$

The genotype 0 is the most abundant genotype at that generation. All the mutants' (mutants are all genotypes that are not the genotype 0) frequencies are kept identical relative to each other, but their sum is set to M_1' (for mutator mutants) or m_1' (for non-mutator mutants).

I. Complex landscapes: eliminating hitchhiking

The stochastic model was run following the usual mutation – phenotypic switching – selection – drift scheme. Right before the drift step, the population vector was recalculated as follows:

$$M_1' = \max(A + p_M \cdot p_S, 0)$$

$$m_1' = \max(p_S - M_1{}', 0)$$

$$M_0' = \max(p_M - M_1{}', 0)$$

$$m_0{}' = \max(1 - M_1' - m_1' - M_0', 0)$$

The genotype 0 is the most abundant genotype at that generation. All the mutants' (mutants are all genotypes that are not the genotype 0) frequencies are kept identical relative to each other, but their sum is set to M_1' (for mutator mutants) or m_1' (for non-mutator mutants).

J. Complex landscapes: limiting p_M

The stochastic model was run following the usual mutation – phenotypic switching – selection – drift scheme. The desired frequency of mutators is equal to M. Right before the drift step, the population vector was recalculated as follows:

$$M_1' = \max(A + p_M \cdot p_S, 0)$$

$$m_1' = \max(p_S - M_1{}', 0)$$

$$M_0' = \max(M - M_1{}', 0)$$

$$m_0{}' = \max(1 - M_1' - m_1' - M_0', 0)$$

The genotype 0 is the most abundant genotype at that generation. All the mutants' (mutants are all genotypes that are not the genotype 0) frequencies are kept identical relative to each other, but their sum is set to M_1' (for mutator mutants) or m_1' (for non-mutator mutants).

References

[1] S. P. OTTO and T. DAY, “Mathematical Modeling in Biology,” in *A Biologist’s Guide to Mathematical Modeling in Ecology and Evolution*, 2019.

[2] K. S. Sarkisyan *et al.*, “Local fitness landscape of the green fluorescent protein,” *Nature*, 2016, doi: 10.1038/nature17995.

[3] C. Li, W. Qian, C. J. Maclean, and J. Zhang, “The fitness landscape of a tRNA gene,” *Science*, vol. 352, no. 6287, pp. 837–840, May 2016, doi: 10.1126/science.aae0568.

[4] J. Aguilar-Rodríguez, J. L. Payne, and A. Wagner, “A thousand empirical adaptive landscapes and their navigability,” *Nat. Ecol. Evol.*, 2017, doi: 10.1038/s41559-016-0045.

[5] S. Wright, “Evolution in Mendelian Populations,” *Genetics*, vol. 16, no. 2, pp. 97–159, Mar. 1931.

[6] M. Serrano, A. W. Lin, M. E. McCurrach, D. Beach, and S. W. Lowe, “Oncogenic ras provokes premature cell senescence associated with accumulation of p53 and p16INK4a,” *Cell*, vol. 88, no. 5, pp. 593–602, Mar. 1997, doi: 10.1016/S0092-8674(00)81902-9.

[7] B. R. Levin, V. Perrot, and N. Walker, “Compensatory Mutations, Antibiotic Resistance and the Population Genetics of Adaptive Evolution in Bacteria,” 2000.

[8] M. V Trotter, D. B. Weissman, G. I. Peterson, K. M. Peck, and J. Masel, “Cryptic genetic variation can make ‘irreducible complexity’ a common mode of adaptation in sexual populations,” *Evolution (N. Y).*, vol. 68, no. 12, pp. 3357–3367, Dec. 2014, doi: https://doi.org/10.1111/evo.12517.

[9] C. K. Griswold and J. Masel, “Complex adaptations can drive the evolution of the capacitor [PSI], even with realistic rates of yeast sex.,” *PLoS Genet.*, vol. 5, no. 6, p. e1000517, Jun. 2009, doi: 10.1371/journal.pgen.1000517.

[10] Y. Kim, “RATE OF ADAPTIVE PEAK SHIFTS WITH PARTIAL GENETIC ROBUSTNESS,” *Evolution (N. Y).*, vol. 61, no. 8, pp. 1847–1856, Aug. 2007, doi: https://doi.org/10.1111/j.1558-5646.2007.00166.x.

[11] Y. Ram and L. Hadany, “Stress-induced mutagenesis and complex adaptation,” *Proc. R. Soc. B Biol. Sci.*, 2014, doi: 10.1098/rspb.2014.1025.

[12] D. J. Whitehead, C. O. Wilke, D. Vernazobres, and E. Bornberg-Bauer, “The look-ahead effect of phenotypic mutations,” *Biol. Direct*, vol. 3, p. 18, May 2008, doi: 10.1186/1745-6150-3-18.

[13] H. Forrest, “Life finds a way,” *Nat. Ecol. Evol. 2022*, vol. 43, no. 2, pp. 1–2, Sep. 2022, doi: 10.1038/s41559-022-01877-x.

[14] E. van Nimwegen and J. P. Crutchfield, “Metastable evolutionary dynamics: crossing fitness barriers or escaping via neutral paths?,” *Bull. Math. Biol.*, vol. 62,

no. 5, pp. 799–848, Sep. 2000, doi: 10.1006/bulm.2000.0180.

[15] U. Obolski, Y. Ram, and L. Hadany, “Key issues review: Evolution on rugged adaptive landscapes,” *Reports on Progress in Physics*. 2018, doi: 10.1088/1361-6633/aa94d4.

[16] S. Kauffman and S. Levin, “Towards a general theory of adaptive walks on rugged landscapes,” *J. Theor. Biol.*, vol. 128, no. 1, pp. 11–45, 1987, doi: https://doi.org/10.1016/S0022-5193(87)80029-2.

[17] J. A. G. M. De Visser, S. C. Park, and J. Krug, “Exploring the effect of sex on empirical fitness landscapes,” *Am. Nat.*, 2009, doi: 10.1086/599081.

[18] J. Aguilar-Rodríguez, L. Peel, M. Stella, A. Wagner, and J. L. Payne, “The architecture of an empirical genotype-phenotype map,” *Evolution (N. Y).*, 2018, doi: 10.1111/evo.13487.

[19] S. Banerjee *et al.*, “Mispacking and the Fitness Landscape of the Green Fluorescent Protein Chromophore Milieu,” *Biochemistry*, 2017, doi: 10.1021/acs.biochem.6b00800.

[20] S. Uphoff, N. D. Lord, B. Okumus, L. Potvin-Trottier, D. J. Sherratt, and J. Paulsson, “Stochastic activation of a DNA damage response causes cell-to-cell mutation rate variation,” *Science (80-.).*, 2016, doi: 10.1126/science.aac9786.

[21] I. Gordo and F. Dionisio, “Nonequilibrium model for estimating parameters of deleterious mutations,” *Phys. Rev. E - Stat. Nonlinear, Soft Matter Phys.*, 2005, doi: 10.1103/PhysRevE.71.031907.

[22] I. Eshel, “On the survival probability of a slightly advantageous mutant gene with a general distribution of progeny size-A branching process model,” *J. Math. Biol.*, 1981, doi: 10.1007/BF00276922.

[23] M. M. Desai and D. S. Fisher, “The balance between mutators and nonmutators in asexual populations,” *Genetics*, 2011, doi: 10.1534/genetics.111.128116.

Supplementary Figures to: Phenotype switching of the mutation rate facilitates adaptive evolution

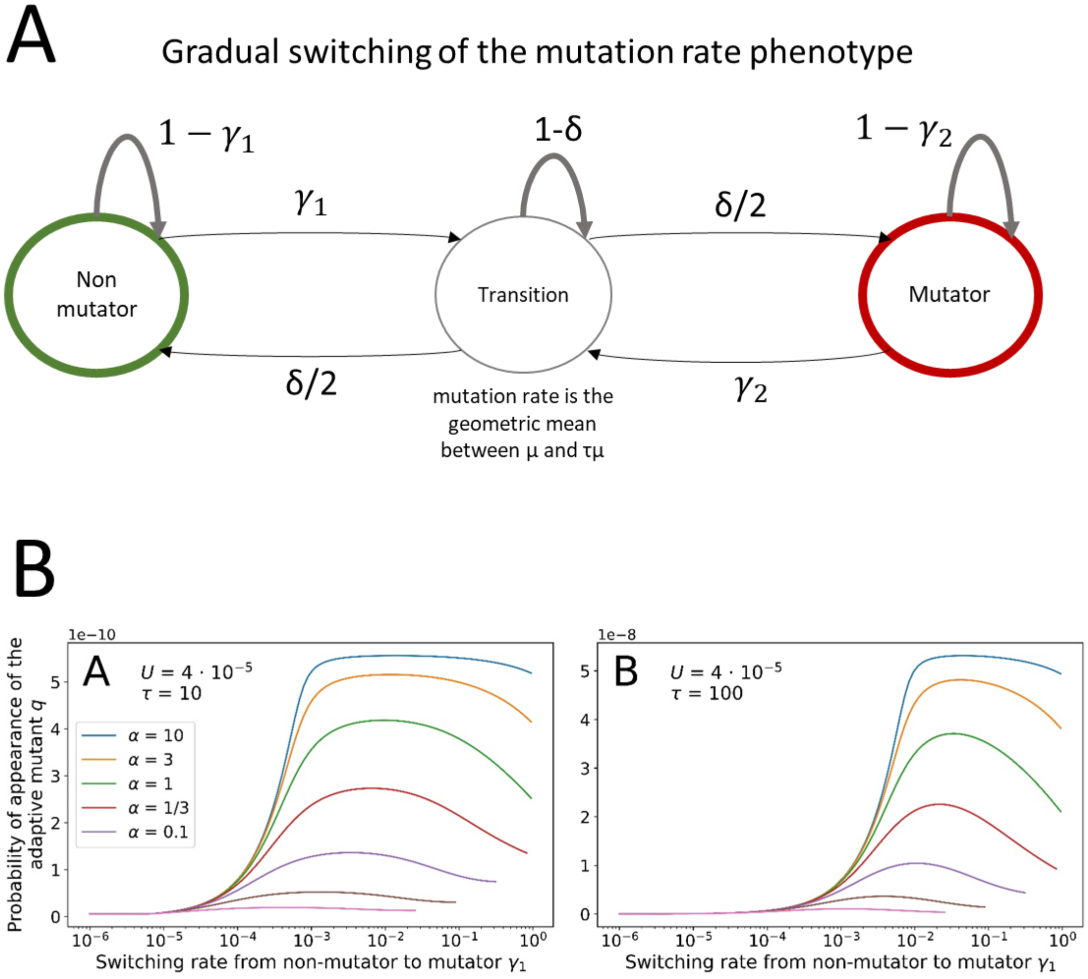

Figure S1. (A) Model extension for gradual switching of the mutation rate phenotype. In this model extension, we introduce an additional, transitional genotype with mutation rate equal to the geometric mean between the non-mutator and the mutator mutation rate. The switching rate γ_1 indicates the switching from the non-mutator to this transition phenotype; a new parameter, $\delta/2$, governs the switching from the transition to the mutator phenotype. By symmetry, we also have the switching rate γ_2 from the mutator to the transition phenotype and $\delta/2$ the probability of switching from the transition phenotype to the non-mutator phenotype. **(B) Predicted probability of appearance of adaptive genotype for model with gradual transition between the non-mutator to the mutator phenotype and vice-versa.** Parameters: $s = 0.1$.

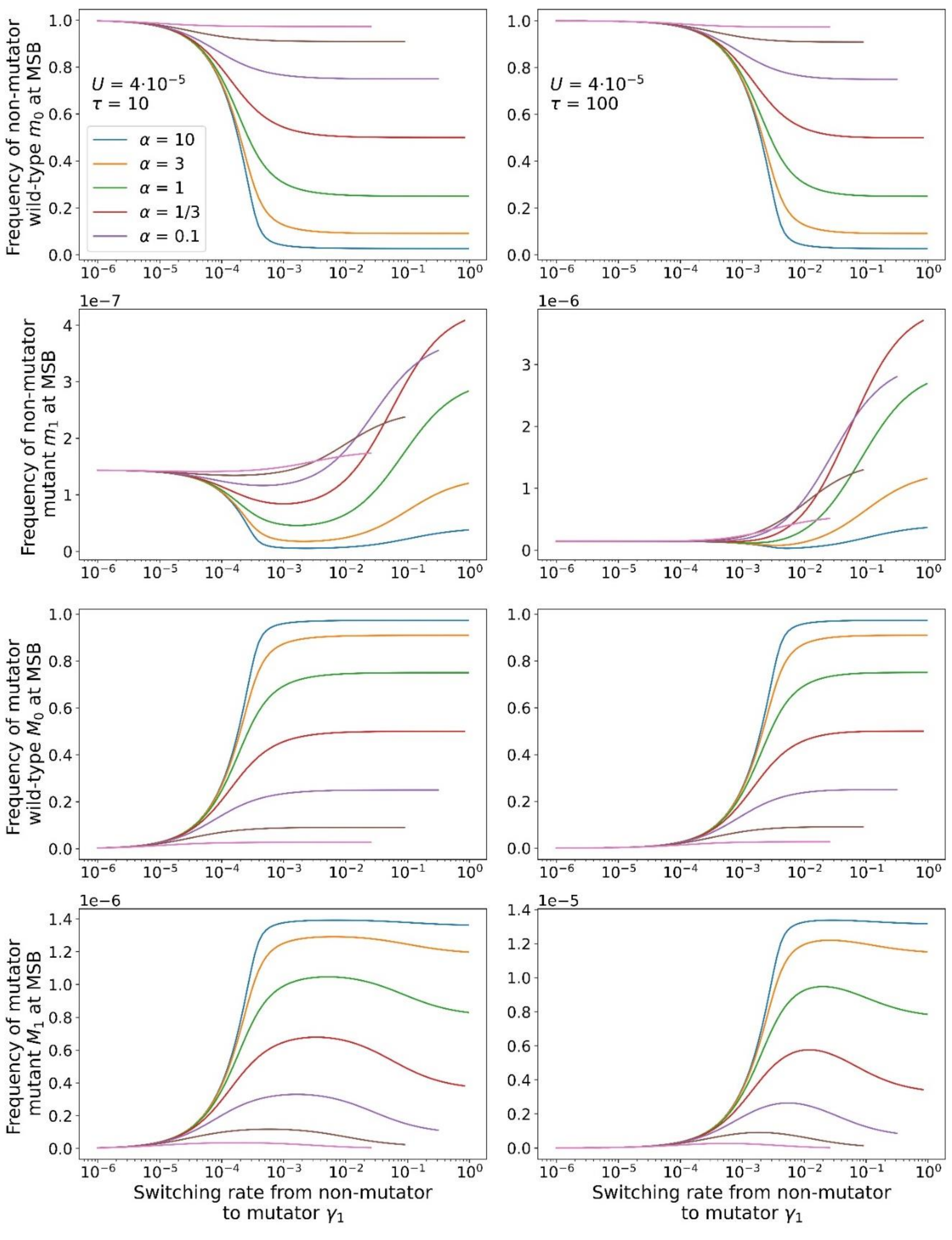

Figure S2. Frequencies of non-mutator wild-type, non-mutator mutant, mutator wild-type, and mutator mutant at MSB for two values of the mutation rate U and two value of the fold-increase in mutator mutation rate τ. Parameters: $s = 0.1$.

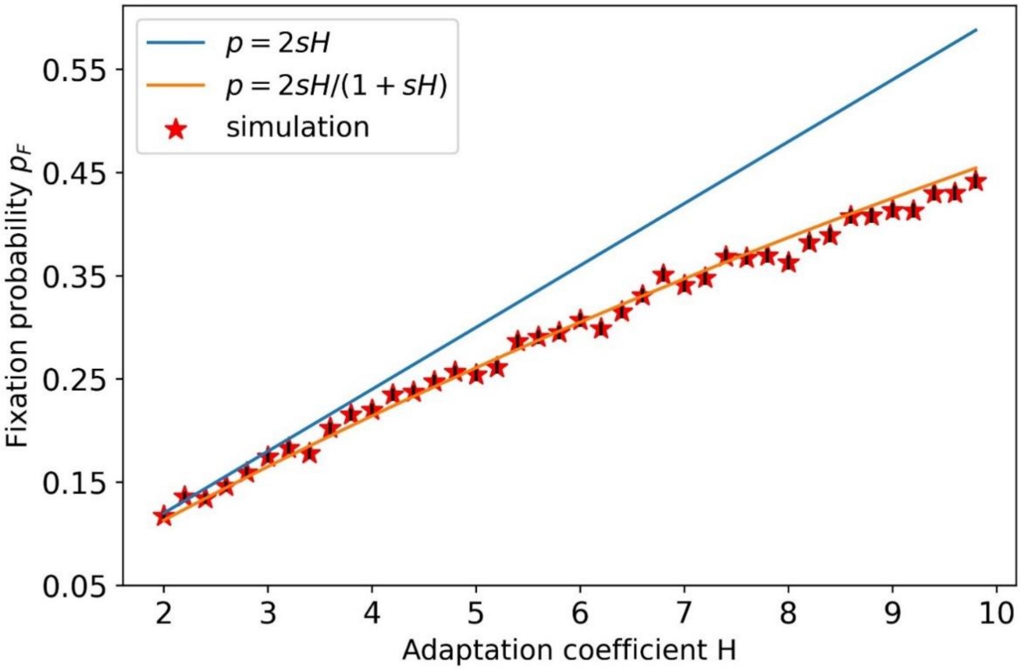

Figure S3. Fixation probability of a rare beneficial genotype. Red stars represent the frequency of fixations in n = 5000 events of adaptive genotype appearance. Error bars (too small to see) show the estimated error $\sqrt{\boldsymbol{p_F(1-p_F)/n}}$. The analytic approximation in Eq. A2a (orange) explains $\boldsymbol{R^2 = 0.9968}$ of the variance in the simulation results, while the approximation Eq. A2b (blue) explains $\boldsymbol{R^2 = 0.9919}$. Here, s = 0.03.

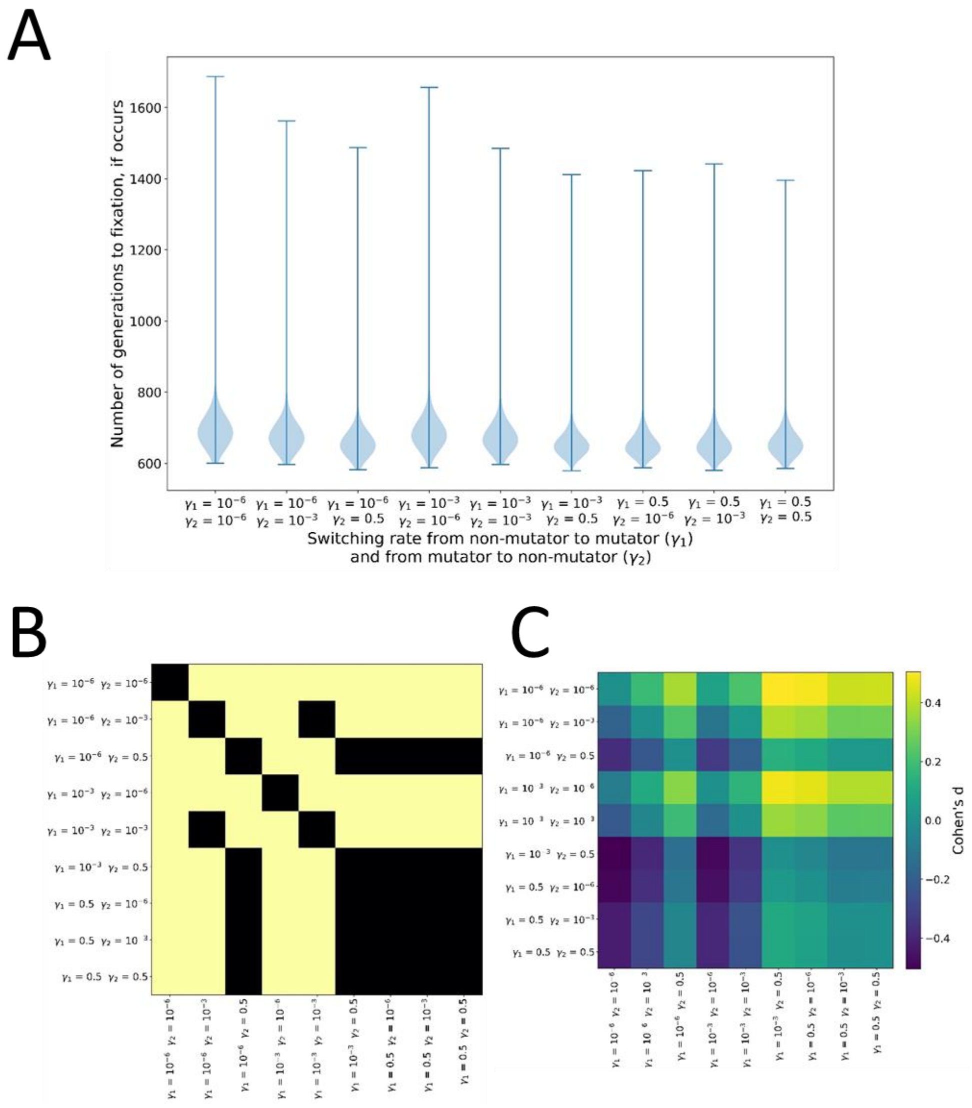

Figure S4. (A) Time to fixation for pairs of γ_1 and γ_2. The time to fixation follows a right-tailed distribution that is not strongly dependent on the values of γ_1 and γ_2. The adaptive mutant had a 6% advantage over the wild-type. **(B) Significance of all pairwise comparisons of time distributions.** A Mann-Whitney test was performed with significance threshold set at 0.01, and a Bonferonni correction was applied to correct for multiple comparisons. Distributions where one switching rate equals 0.5 seem to not be significantly different from one another. Yellow represents statistical significance, black represents lack of statistical significance. **(C) Effect size of the difference between the pairs of the time distributions**. Calculated with Cohen's d. Maximal effect size is less than 0.5, which is quite small.

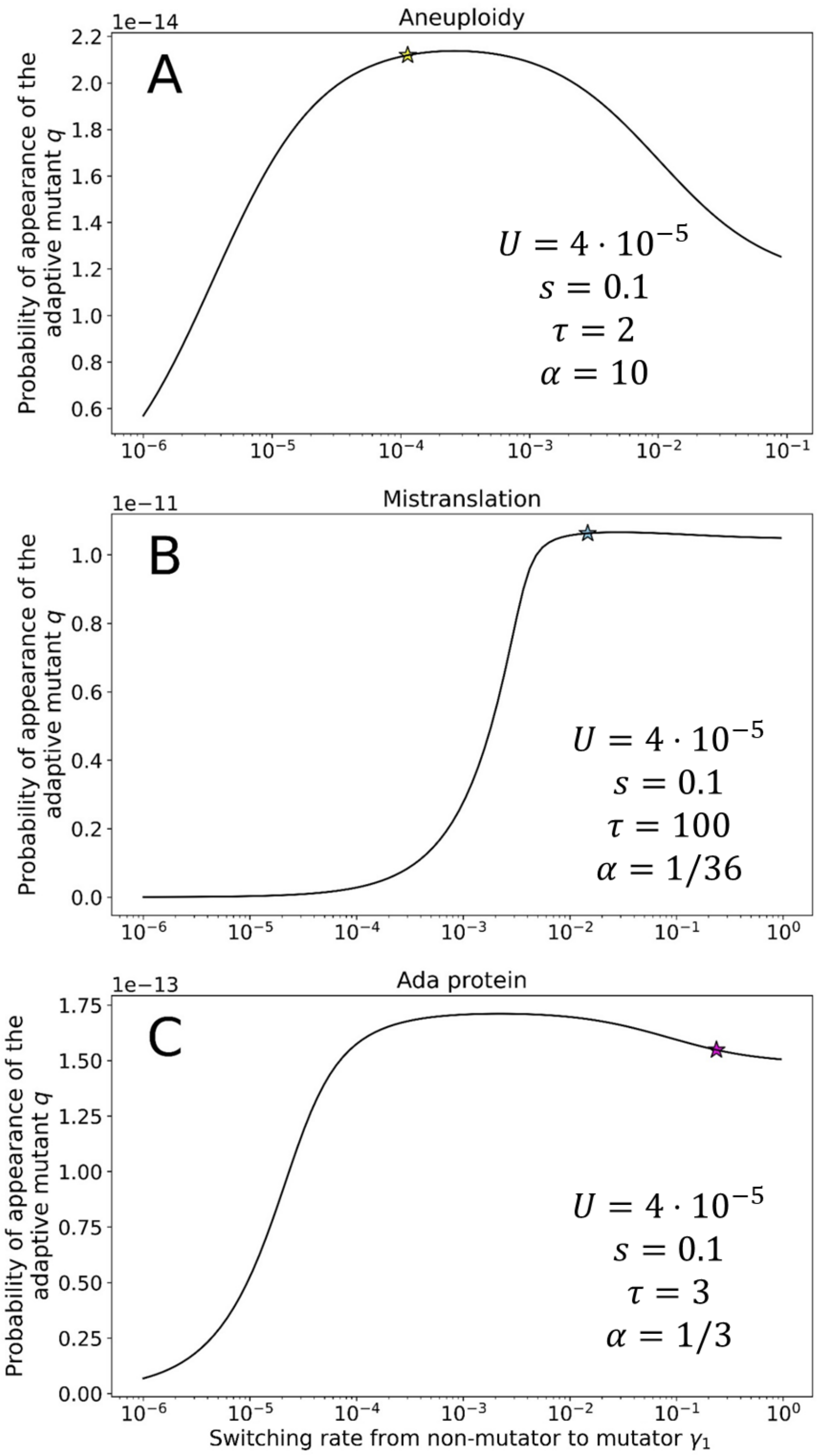

Figure S5. Probability of appearance of the adaptive mutant q for the three empirically described systems for non-genetic inheritance of the mutation rate. We estimated the specific values of the α and τ parameter for each of the three systems. The coloured star was plotted at its estimated value of γ_1 on the x-axis.

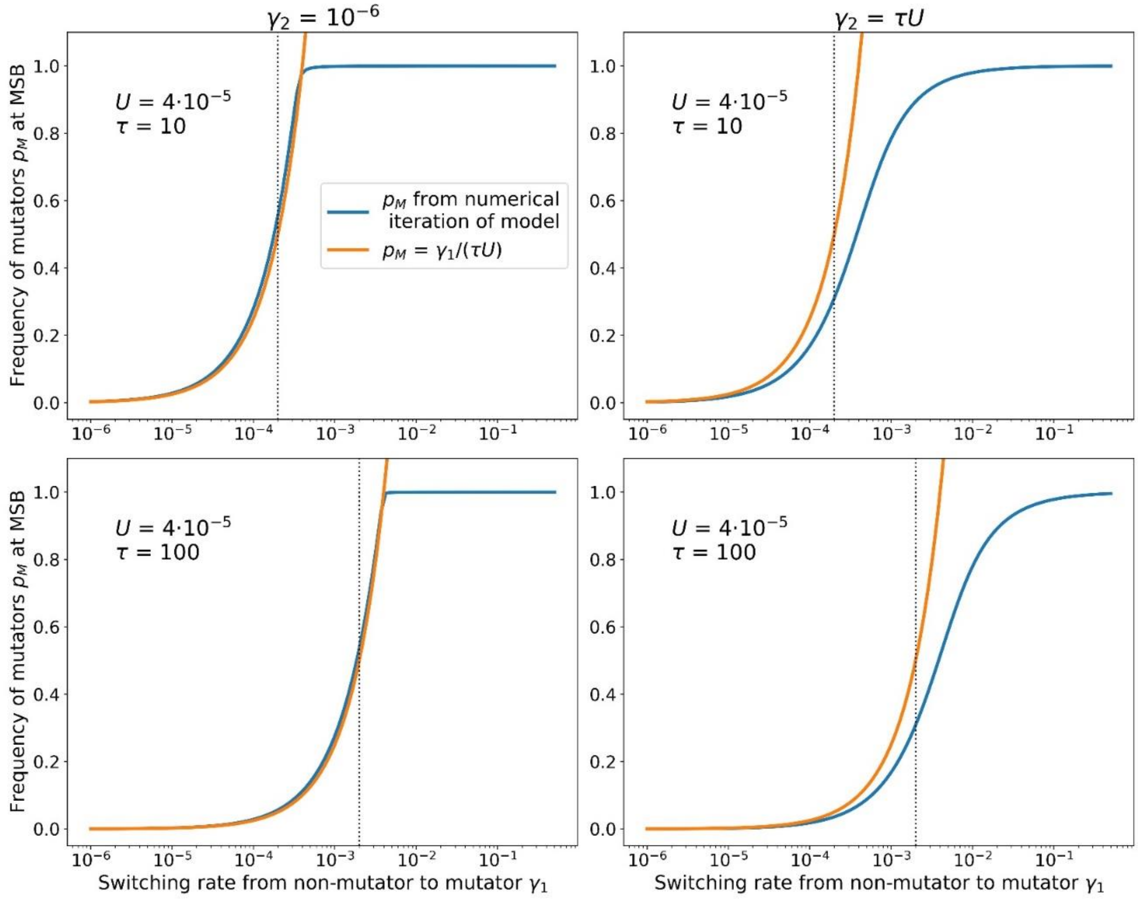

Figure S6. Comparison of the proportion of mutators from the numerical simulation with the analytical prediction derived by [23]**.** The two considered switching rates from mutator to non-mutator γ_2 correspond to genetic inheritance ($\gamma_2 = 10^{-6}$) and the highest value of γ_2 for which we use Eq. 4 ($\gamma_2 = \tau U/2$). The dashed line corresponds to $\gamma_1 = \tau U/2$. For $\gamma_2 = 10^{-6}$, we observe an excellent fit for all considered parameter sets. For $\gamma_2 = \tau U/2$, the fit worsens progressively with increasing switching rate from non-mutator to mutator γ_1. Parameters: $s = 0.03$, $n = 5000$.

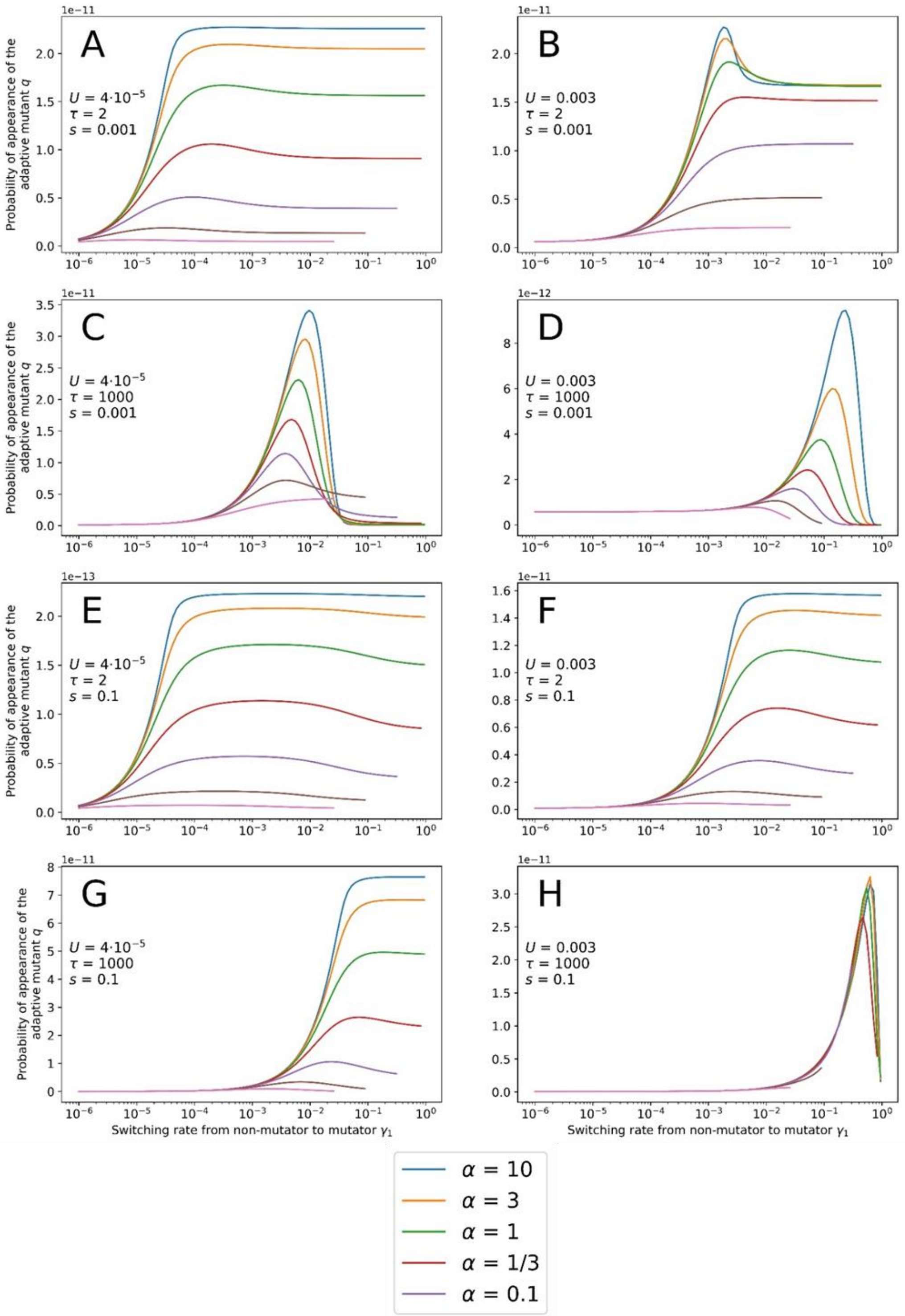

Figure S7. Probability of appearance of the adaptive genotype q for extreme values of U, τ, and s. The probability of appearance was calculated with Eq, 5 and the MSB frequencies obtained for each parameter set.

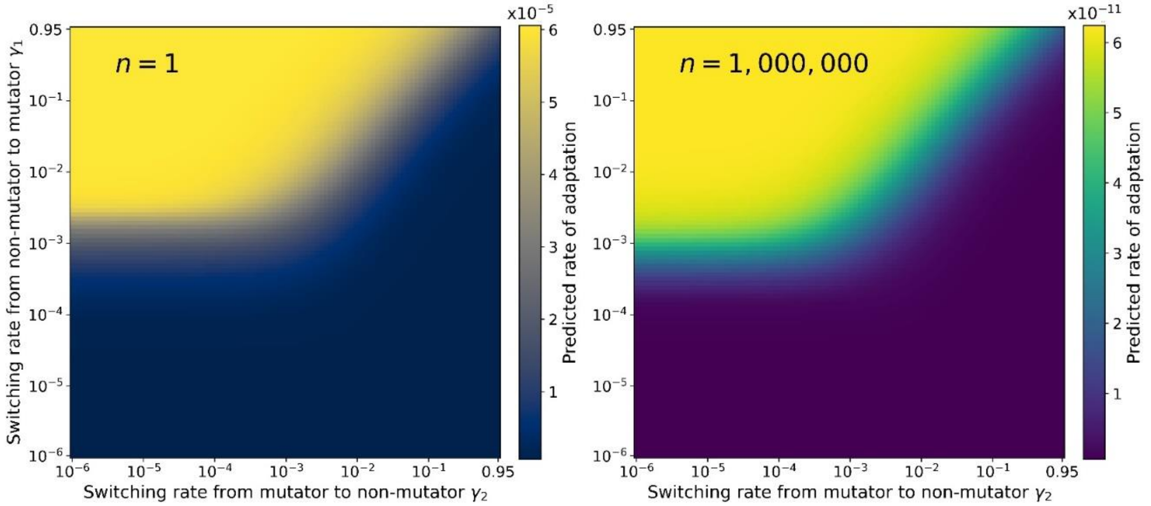

Figure S8. Sensitivity analysis for the number of loci *n*. Although the rate of adaptation is 6 orders of magnitude lower for $n = 1{,}000{,}000$, the relative rate of adaptation is similar for the two values of n. Indeed, the highest rates of adaptation are observed for $\gamma_1 > \frac{\tau U}{2}$ when $\gamma_2 < \frac{\tau U}{2}$ and for $\gamma_1 > \gamma_2$ when $\gamma_2 > \frac{\tau U}{2}$. $U = 0.0001$, $s = 0.03$, $\tau = 10$.

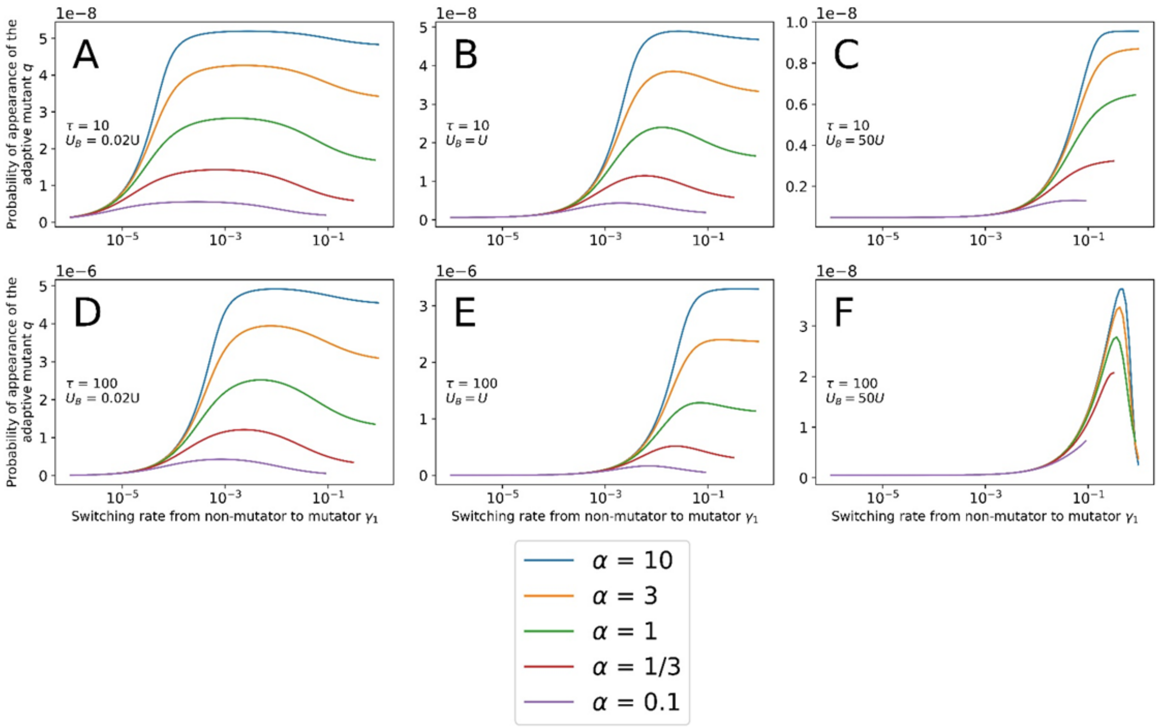

Figure S9. The adaptation-optimal switching rate increases with the deleterious mutation rate, but drops sharply above a critical threshold. Each plot shows the probability of appearance rate of the adaptive mutant q along the switching rate from non-mutator to mutator γ_1. Parameters: $U = 4 \cdot 10^{-4}$, $s = 0.1$, $n = 5{,}000$.

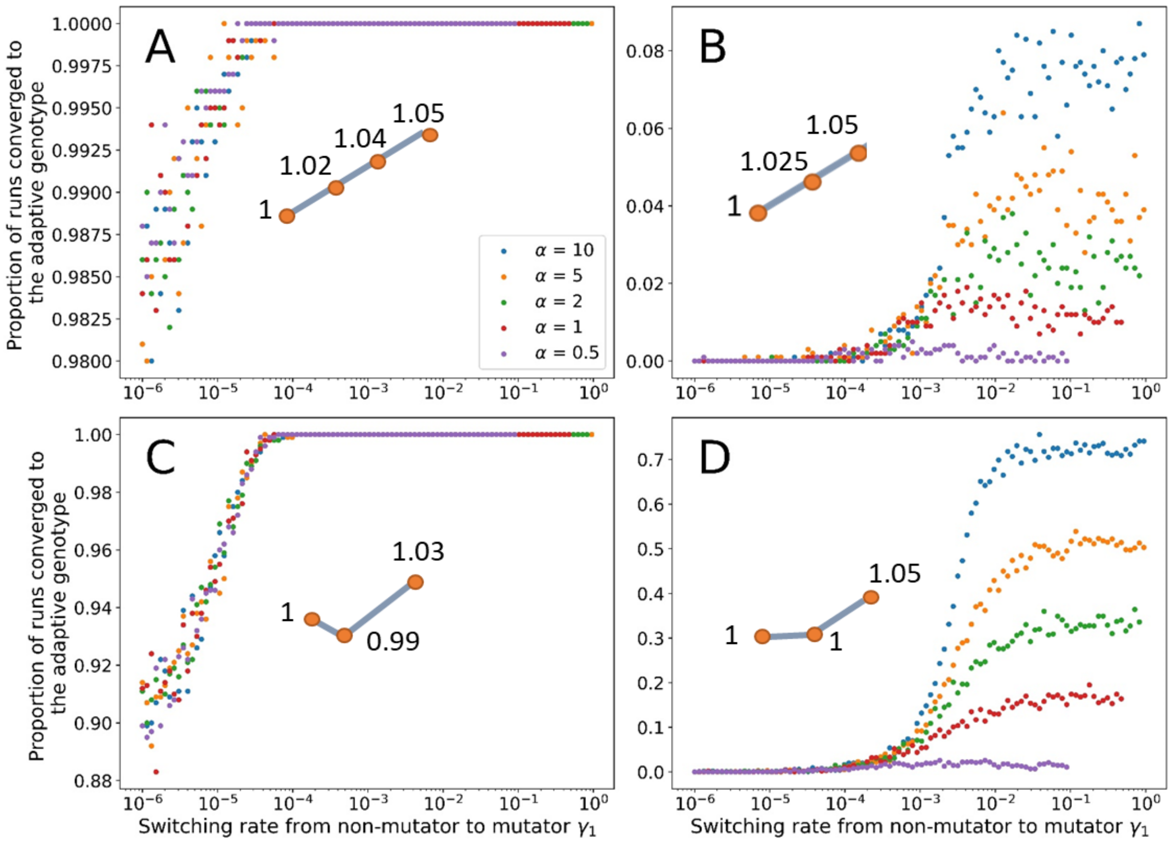

Figure S10: Adaptation rate for several fitness motifs. Same as Figure 5, but for additional fitness landscape motifs.

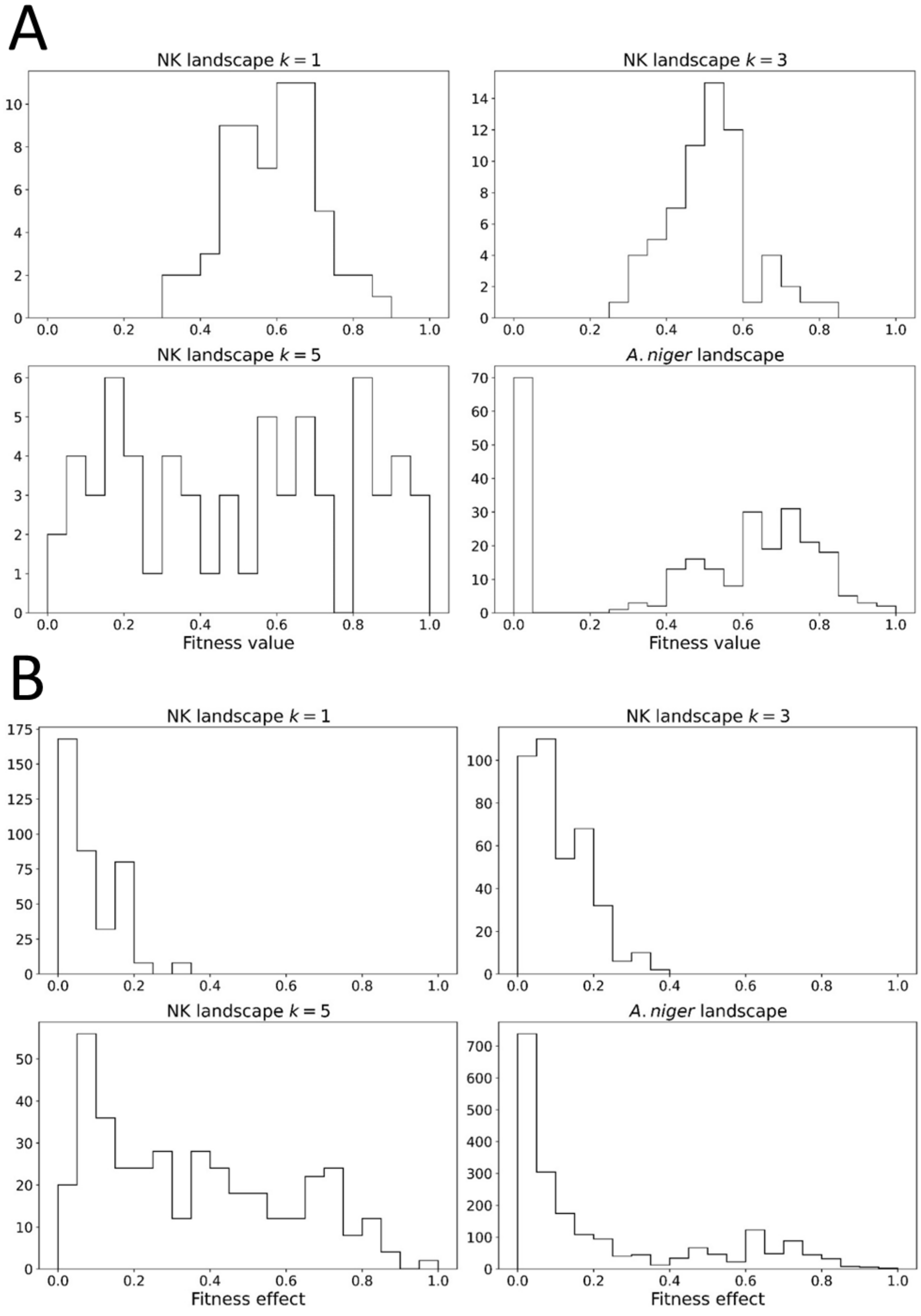

Figure S11: (A) Distribution of fitness values in the NK and *Aspergillus niger* landscapes. In the NK landscapes, the higher the $\boldsymbol{k}$ (representing the ruggedness), the wider the distribution of fitness values. **(B) Distribution of fitness effects in the NK and *Aspergillus niger* landscapes.** A histogram of the mean difference between each genotype and its single mutants for different values of the ruggedness parameter $\boldsymbol{k}$. As expected, as $\boldsymbol{k}$ increases, the distribution is wider.

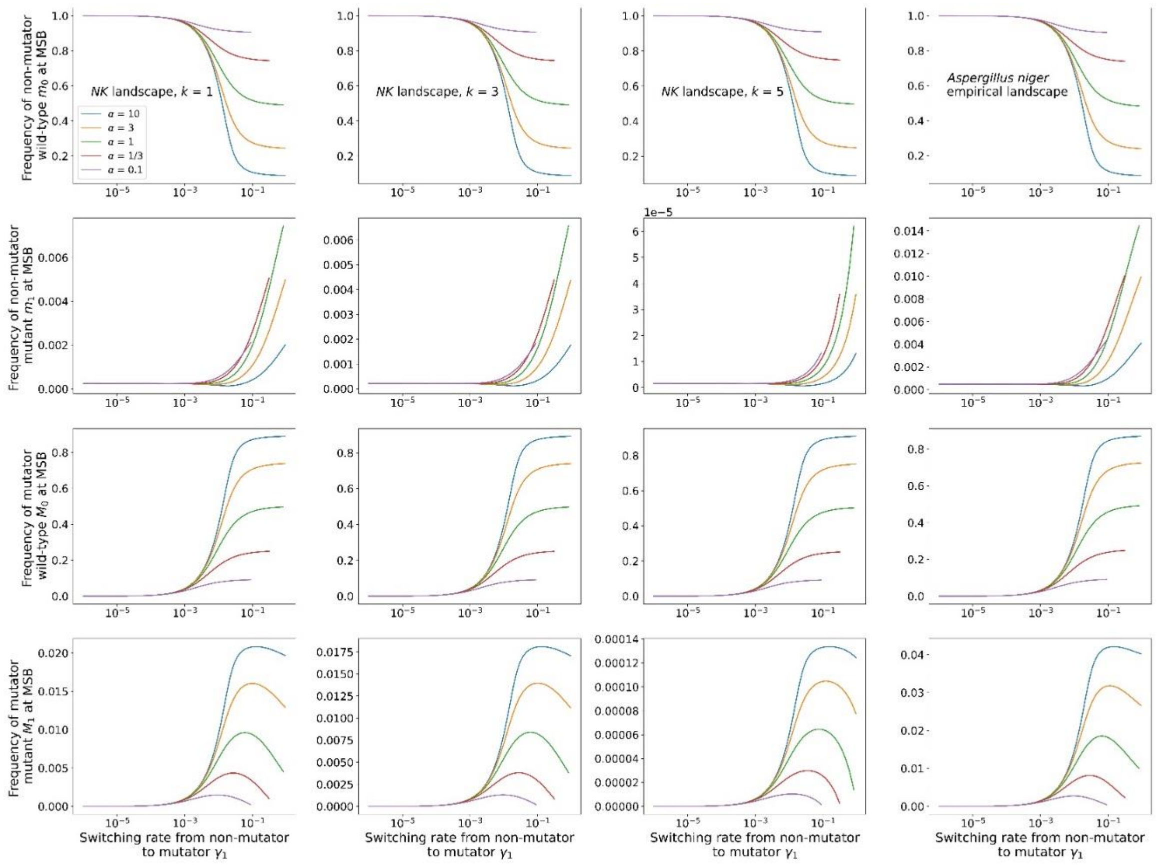

Figure S12: Frequencies of non-mutator wild-type, non-mutator mutant, mutator wild-type, and mutator mutant at MSB for two values of the mutation rate *U* and two value of the fold-increase in mutator mutation rate τ, for the four considered complex landscapes: the *Aspergillus niger* empirical landscape, and three *NK* landscapes of varying ruggedness. Parameters: $U = 4 \cdot 10^{-5}$, $\tau = 100$.

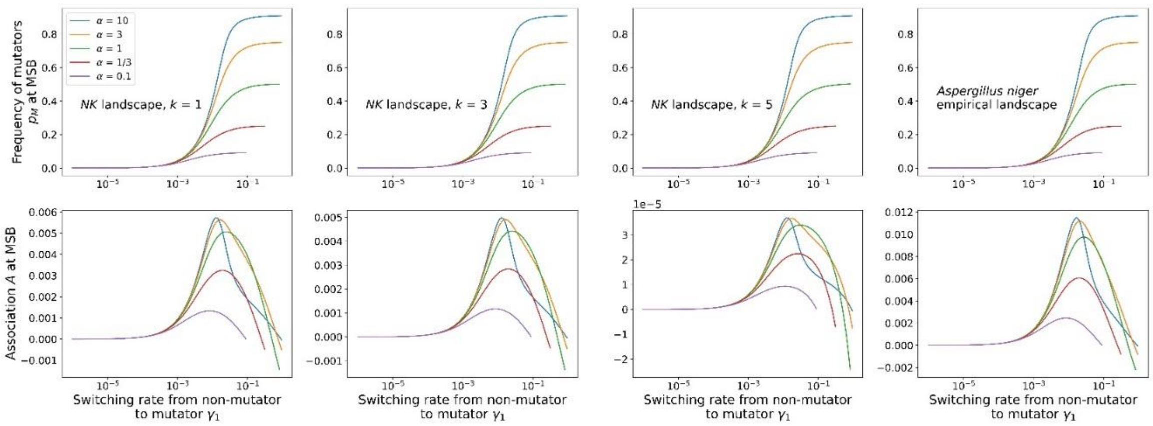

Figure S13: Frequency of mutators p_M at MSB and association A at MSB for the four considered complex landscapes: the *Aspergillus niger* empirical landscape, and three *NK* landscapes of varying ruggedness. The proportion of mutators p_M is obtained directly from the MSB frequencies. The association A is calculated from the MSB frequencies according to Eq. 8. Parameters: $U = 4 \cdot 10^{-5}$, $\tau = 100$.

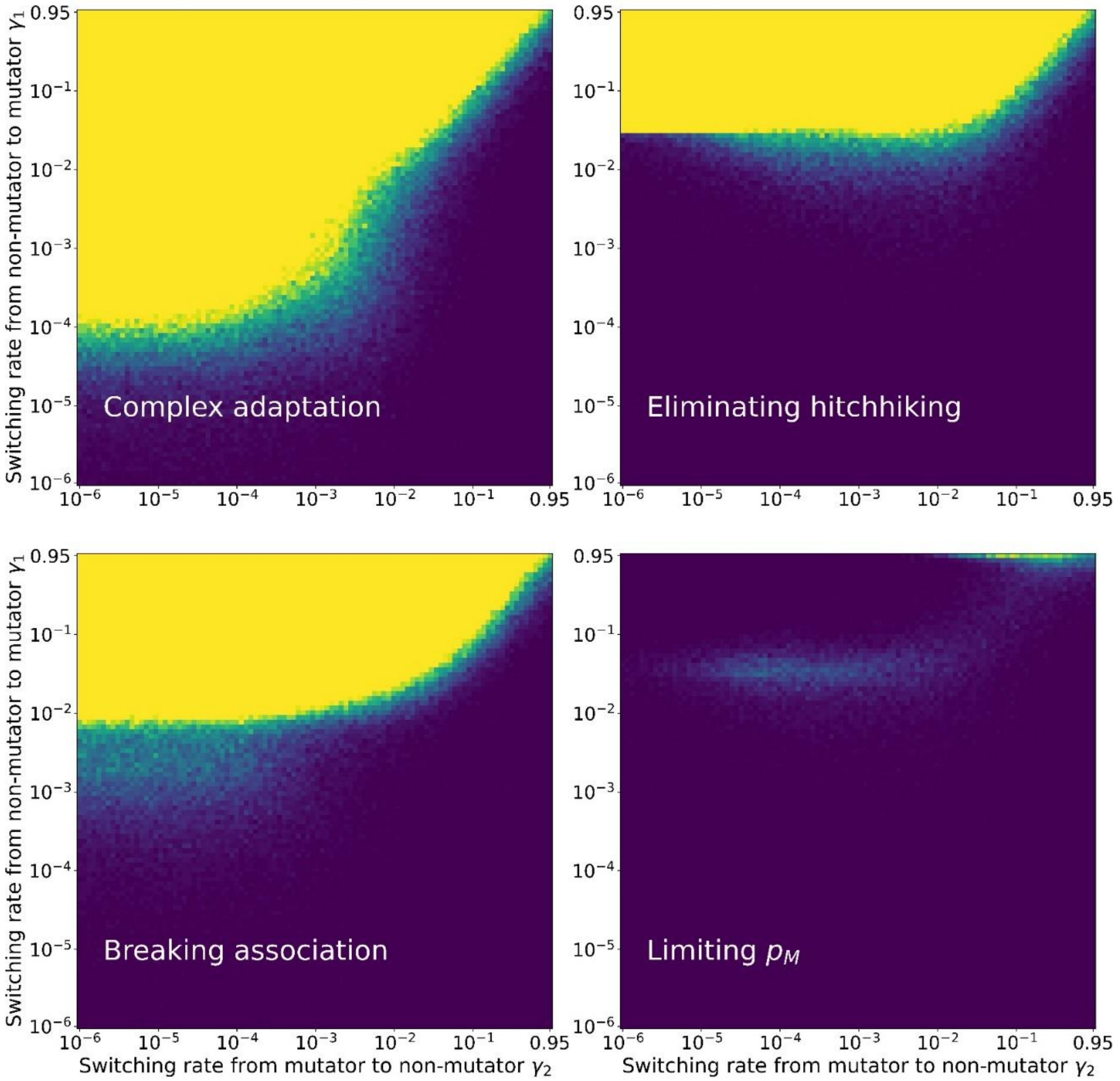

Figure S14: Complex adaptation on *Aspergillus niger* landscape for all combinations of values of the two switching rates γ_1 and γ_2. Same as Figure 6, but for all possible values of γ_1 and γ_2. Parameters: $U = 4 \cdot 10^{-5}$, $\tau = 100$, $N = 1000$.

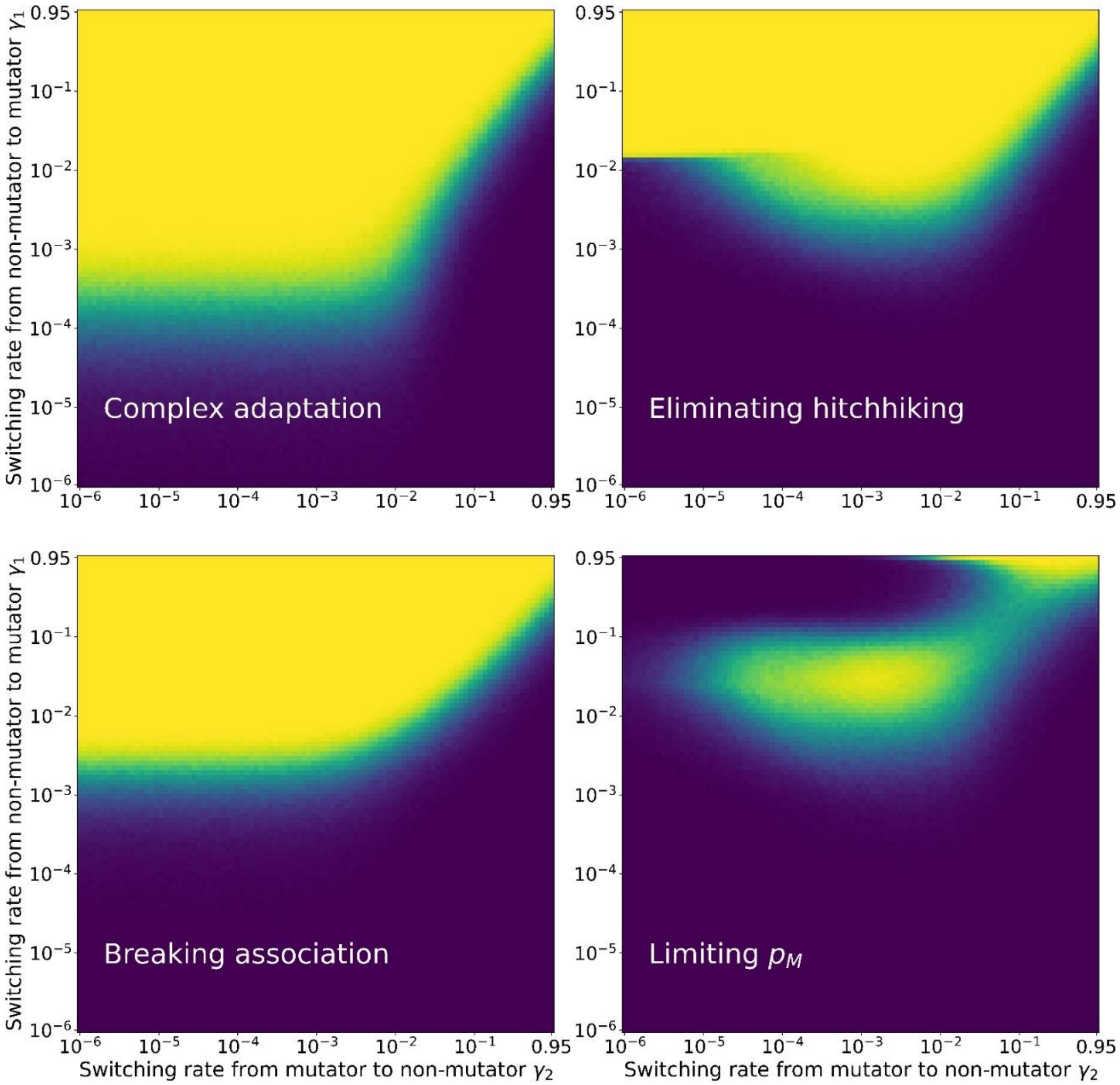

Figure S15: Complex adaptation on NK landscape, *k*=*1*. The proportion of runs out of 1000 that converged upon the fittest genotype in the landscape was recorded after 500 generations. In order to disentangle the different effects of the non-genetic inheritance of the mutation rate on the rate of adaptation, we then rerun the simulation while removing the association of mutator and mutant, limiting the frequency of mutators during the evolution, and eliminating hitchhiking. Note that hitchhiking is also eliminated when the association between mutator and mutant is broken, or the frequency of the mutator limited. We observe that the region with rates of adaptation for $\gamma_1 < \tau U/2$ and $\gamma_2 < \tau U/2$ disappears when hitchhiking is eliminated. Limiting the frequency p_M reduces adaptation overall. Parameters: $U = 4 \cdot 10^{-5}$, $\tau = 100$, $N = 1000$.

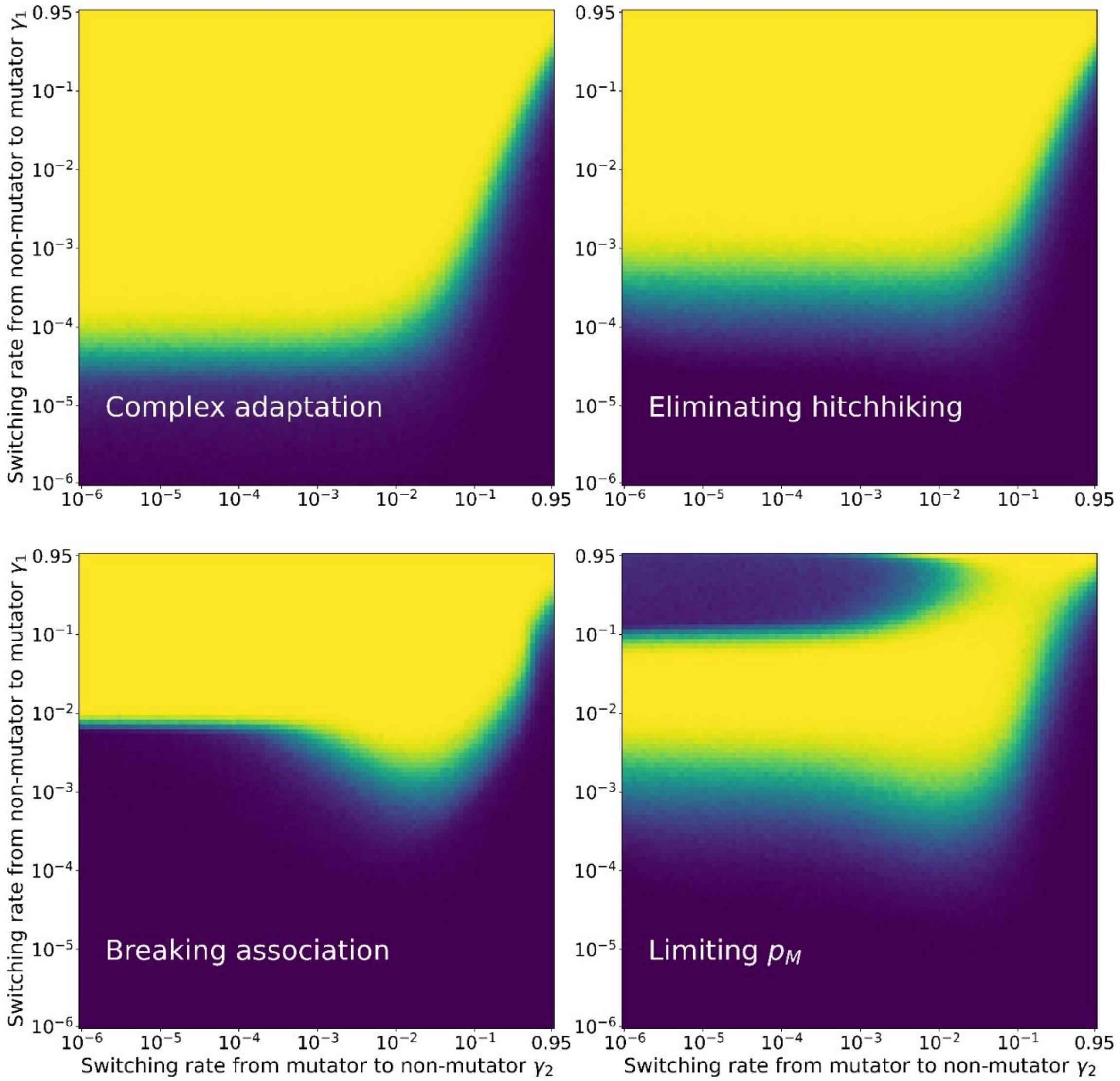

Figure S16: Complex adaptation on NK landscape, *k*=3. Same as Figure S15, but for k=3. Parameters: $U = 4 \cdot 10^{-5}$, $\tau = 100$, $N = 10^7$.

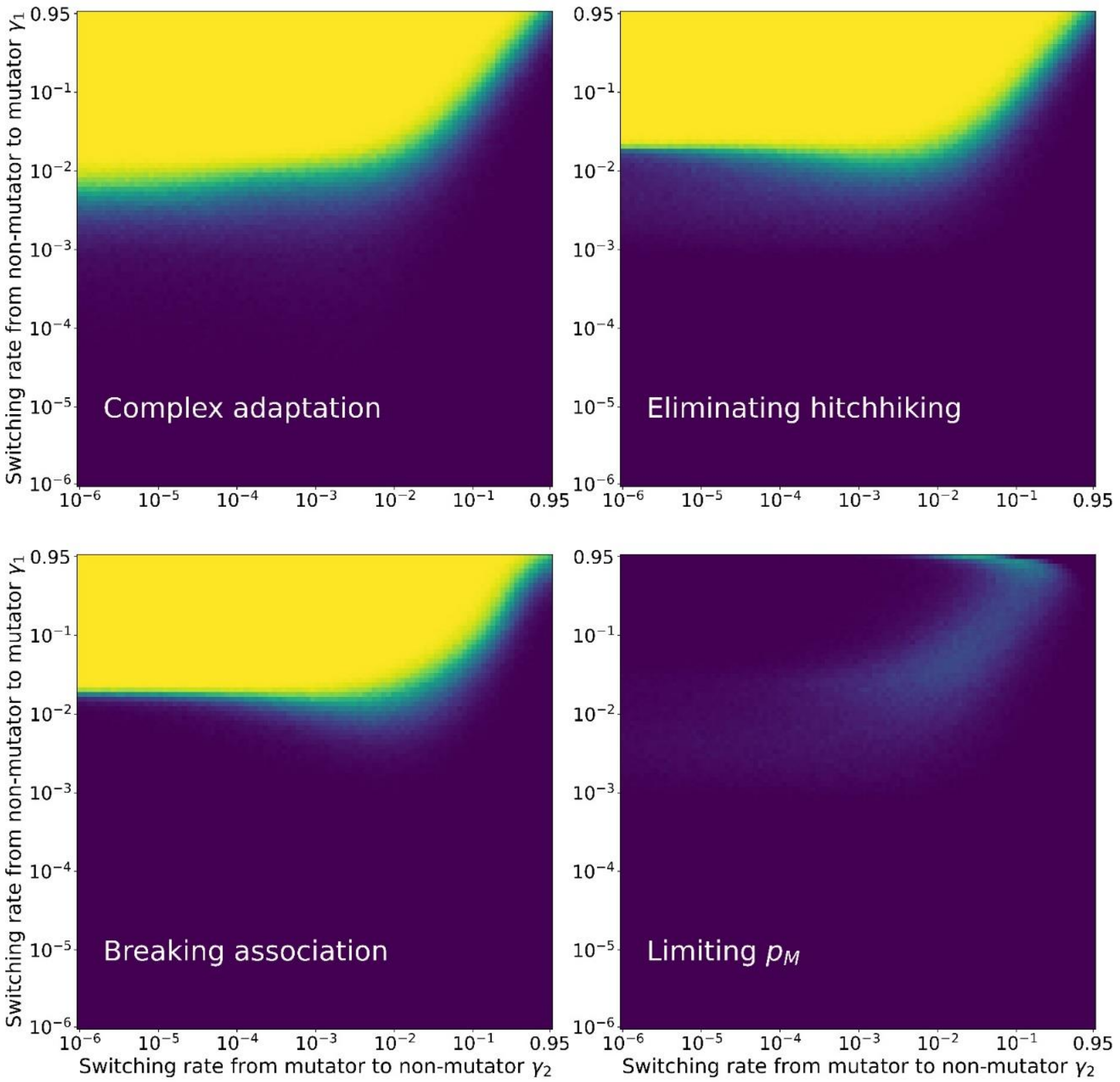

Figure S17: Complex adaptation on NK landscape, *k*=*5*. Same as Figure S15, but for k=*5*. Parameters: $U = 4 \cdot 10^{-5}$, $\tau = 100$, $N = 10^7$.

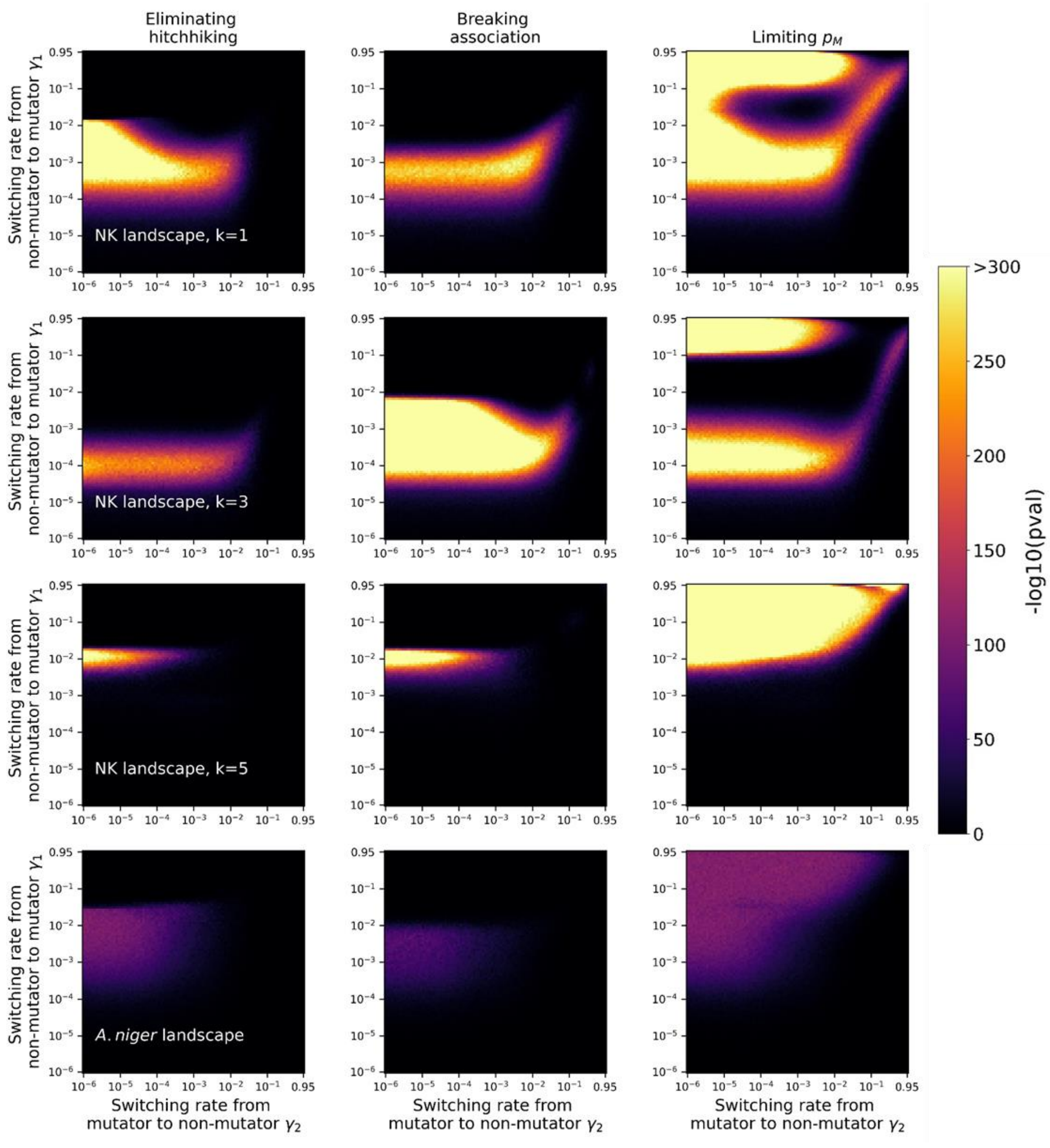

Figure S18: Statistical analysis of the rate of adaptation in the complex adaptation simulation and its modifications: eliminating hitchhiking, reducing the proportion of mutators and breaking association between mutators and mutants. A two-proportion Z-test was performed between the proportion of runs that converged on the adaptive genotype in the complex adaptation simulation and between the proportion of runs that converged on the adaptive genotype in a modification of the simulation. We report the –log10 of the obtained p-value.
